# Characterizing localized nitrogen sensitivity of tree species and the associated influences of mediating factors

**DOI:** 10.1002/ecs2.4925

**Published:** 2024-07-03

**Authors:** Justin G. Coughlin, Shih Ying Chang, Kenneth Craig, Charles Scarborough, Charles T. Driscoll, Christopher M. Clark, Nathan R. Pavlovic

**Affiliations:** 1 Sonoma Technology, Inc., Petaluma, California, USA; 2 Department of Civil and Environmental Engineering, Syracuse University, Syracuse, New York, USA; 3 U.S. Environmental Protection Agency, Office of Research and Development, Washington, DC, USA

**Keywords:** atmospheric deposition, critical loads, machine learning, mediating factors, nitrogen, plant nutrient cycling, terrestrial ecosystem, tree growth, tree mortality

## Abstract

Critical loads (CLs) are frequently used to quantify terrestrial ecosystem impacts from nitrogen (N) deposition using ecological responses such as the growth and mortality of tree species. Typically, CLs are reported as a single value, with uncertainty, for an indicator across a species’ entire range. Mediating factors such as climate and soil conditions can influence species’ sensitivity to N, but the magnitudes of these effects are rarely calculated explicitly. Here, we quantify the spatial variability and estimation error in N CLs for the growth and survival of 10 different tree species while accounting for key environmental factors that mediate species sensitivity to N (e.g., soil characteristics). We used a bootstrapped machine learning approach to determine the level of N deposition at which a 1% decrease occurs in growth rate or survival probability at forest plot locations across the United States. We found minimal differences (<5 kg N ha^−1^ year^−1^) when comparing a single species’ CLs across climatic regimes but found considerable variability in species’ local N CLs (>8.5 kg N ha^−1^ year^−1^) within these regimes. We also evaluated the most important factors for predicting tree growth rates and mortality and found that climate, competition, and air pollution generally have the greatest influence on growth rates and survival probability. Lastly, we developed a new probability of exceedance metric for each species and found high likelihoods of exceedance across large portions (46%) of some species’ ranges. Our analysis demonstrates that machine learning approaches provide a unique capability to: (1) quantify mediating factor influences on N sensitivity of trees, (2) estimate the error in local N CL estimates, and (3) generate localized N CLs with probabilities of exceedance for tree species.

## INTRODUCTION

Global anthropogenic nitrogen (N) fluxes have outpaced natural fluxes since the mid-19th century, causing a 10-fold increase in global N deposition from pre-industrial levels (Galloway et al., 2004). Reactive nitrogen (Nr), which includes all biologically and chemically reactive N species, is an essential nutrient within terrestrial ecosystems that is critical for the primary production of various plant species (LeBauer & Treseder, 2008). There are three primary sources of Nr to terrestrial ecosystems: biological nitrogen fixation, soil mineralization, and atmospheric deposition. While atmospheric deposition continues to be an important anthropogenic Nr source globally (Galloway et al., 2008), regulatory approaches to reduce emissions of nitrogen oxides (${\text{NO}}_{\text{x}}$) have led to decreased Nr deposition in many regions of the United States (Du et al., 2014; Gilliam et al., 2019). For example, decreases in ${\text{NO}}_{\text{x}}$ emissions have directly led to reductions of oxidized N deposition in the form of wet and dry nitrate (${{\text{NO}}_{3}}^{-}$) throughout the northeastern United States (Butler et al., 2003, 2005, 2011). Conversely, deposition of reduced N (${\text{NH}}_{\text{x}}$) forms and gas phase ammonia (${\text{NH}}_{3}$) have remained steady or even increased over the last 20 years due to (1) agricultural and on-road emission sources (Butler et al., 2016; Fenn et al., 2018; Sabo et al., 2019), (2) successes of policies regulating ${\text{NO}}_{\text{x}}$ and sulfur oxide emissions leading to less partitioning of gaseous ${\text{NH}}_{\text{x}}$ to the particulate phase (Li et al., 2016; Saylor et al., 2015; Zhang et al., 2018), and (3) increased temperature and precipitation in some regions of the United States (Benish et al., 2022). Owing to the inverse national trends of oxidized N (35% lower) and ${\text{NH}}_{\text{x}}$ (30% higher) deposition, average total Nr deposition has only modestly decreased (~12.5%) over the last 20 years, and thus remains an important environmental stressor within ecosystems (Beachley et al., 2019).

While plant species can experience enhanced growth from Nr deposition, there are also unintentional negative consequences within terrestrial ecosystems that result from excessive Nr deposition—otherwise called terrestrial eutrophication. These detrimental effects can include biodiversity loss through opportunistic species outcompeting native herbaceous or understory species (Clark et al., 2019; Simkin et al., 2016; Stevens et al., 2010); changes in forest carbon stocks (Clark, Thomas, & Horn, 2023; Thomas et al., 2010), shifts in forest community composition, and/or differential impacts across species due to variable N sensitivity (Clark, Phelan, et al., 2023; Coughlin et al., 2023); ${{\text{NO}}_{3}}^{-}$ leaching losses leading to shifts toward more acid-tolerant and nitrophilic species within terrestrial ecosystem (Bobbink et al., 2010; Högberg et al., 2006; Matson et al., 2002) and increases in ${{\text{NO}}_{3}}^{-}$ export to fresh and coastal waters (Driscoll et al., 2003; Gilliam et al., 2019); soil acidification potentially leading to decreased buffering capacity and nutrient imbalances in tree foliage or phloem (Bowman et al., 2008; Likens et al., 1996; Sullivan et al., 2013); increased tree mortality in areas of N saturation because of compounding factors such as drought (Dietze & Moorcroft, 2011; Wallace et al., 2007); and consequences to ecosystem services (Clark et al., 2017). To protect terrestrial ecosystems, regulatory agencies and ecological researchers have established a framework to evaluate the threshold (i.e., critical load [CL]) below which harmful ecological impacts are not expected to occur given current knowledge (Nilsson, 1988).

Significant advances have been made in quantifying CLs for terrestrial ecosystem components over the last decade (Blett et al., 2014). CLs have been established for herbaceous and shrub species (Clark et al., 2019; Simkin et al., 2016; Wilkins et al., 2022), lichen species and functional groups (Geiser et al., 2019, 2021), tree species (Horn et al., 2018; Pavlovic et al., 2023), and regional areas (Pardo et al., 2011). Notably, recent research established dose–response relationships for tree species, which evaluated N deposition as an environmental stressor on the growth and mortality of trees (Canham & Murphy, 2016, 2017; Fenn et al., 2020; Horn et al., 2018; Pavlovic et al., 2023; Thomas et al., 2010). Some dose–response evaluations have utilized a maximum-likelihood approach and included a variety of environmental conditions or stressors (temperature, precipitation, competition, size, and sulfur deposition) to estimate the growth rate and survival probability of different tree species as a function of N deposition (Fenn et al., 2020; Horn et al., 2018; Thomas et al., 2010). A key strength of these approaches is their adherence to a predetermined functional form guided by ecological principles (Canham & Murphy, 2016, 2017). A key limitation is their computational burden and their current inability to account for spatial variation in sensitivity to N deposition—which is expected and known to exist for other taxonomic groups (Clark et al., 2019; Pardo et al., 2011). An alternative approach was developed using the eXtreme Gradient Boosting (XGBoost) machine learning (ML) algorithm, a non-parametric approach that retains all predictors, and statistical predictive capability was improved (i.e., increased coefficient of determination) when estimating tree growth rates (Pavlovic et al., 2023). The ML method also quantified CL uncertainties for tree species across the United States (Pavlovic et al., 2023), suggesting that spatially variable CLs could be quantifiable for single species while accounting for variable environmental conditions (i.e., mediating factors).

The effects of N deposition on trees are thought to be primarily mediated by soil conditions (Carter et al., 2017). However, other factors, such as species’ physiological traits, atmospheric interactions, local competition, mycorrhizal associations, secondary stressors, and ozone, can also modify the sensitivity of tree species to N deposition (Bobbink et al., 2010; Fenn et al., 2020; Horn et al., 2018; Thomas et al., 2010). Soil conditions can be widely variable, even within localized areas (Hynicka et al., 2016). These variations may be due to soil age, topography, climate, water imbalances, or underlying geology—all of which have substantial influences on soil development, nutrient availability, and buffering capacity (Fenn et al., 1998; Jenny, 1994). In addition to soil conditions, trees of the same species but in different locations may vary in their sensitivity to N due to stressors such as temperature extremes, drought, community composition, competition with other species, climate change, invasive species, and other pollutants (e.g., ozone, sulfur dioxide) (Carter et al., 2017). It is therefore expected that N CLs for a given tree species will be spatially variable due to the heterogeneity of mediating factors, an effect which has previously been demonstrated for herbaceous species (Clark et al., 2019).

Here, we build upon research from Pavlovic et al. (2023) to quantify spatially variable N CLs for tree species using the XGBoost ML software library with independent conditional expectation (ICE) (Chen & Guestrin, 2016; Goldstein et al., 2015). XGBoost implements a supervised ML algorithm which uses gradient boosting decision trees to predict outcomes by sequentially adding decision trees to the model. A major innovation of this work is the use of ICE curves to quantify the sensitivity of individual tress based on mediating environmental factors. We selected 10 tree species that are of ecological or economic importance to develop and test the ICE approach. For these 10 species, we quantified the spatial variability of N CLs and compared our results to previously determined, species-wide CLs (additional species results are contained in Appendix S1). To investigate the specific influence of individual mediating factors, we used SHapley Additive exPlanations (SHAP) values to identify the most influential predictors in growth rate and survival probability outcomes.

Our study includes a wider array of predictors compared to prior empirical work that quantified N CLs. These include drought indices, soil pH, percent organic matter, percent clay, and ozone concentrations. We also developed a new metric for determining the probability that an area is experiencing detrimental levels of N deposition at the individual tree level. This approach extends our prior work by quantifying CL uncertainties at an individual tree level. N CLs have traditionally been evaluated at the national level across a species’ distributional range. In contrast, our study provides a locality-based N CL approach that incorporates statistical uncertainty. These results can be leveraged by resource managers and policymakers to evaluate whether additional air pollution measures are needed in regional, or even local, areas, and to appropriately account for this fine-scale variability in national-level decision making.

## METHODS

### Forest inventory data

We compiled forest inventory data from the 2021 United States Forest Service (USFS) Forest and Inventory Analysis (FIA) program database, which covered years between 2000 and 2021; these data were fuzzed and swapped to protect private landowner information. The forest inventory dataset was compiled for 140 tree species using procedures that have been previously applied (Horn et al., 2018; Pavlovic et al., 2023). Briefly, the FIA program collects tree characteristic data including tree height, diameter, and survival status for all trees with a dbh of >12.7 cm (Woudenberg et al., 2010). Here, we only used trees that were larger than seedlings, so our results will be reflective of stand-level effects across local areas. The FIA program is operated using a nationally standardized plot design where a plot consists of macroplots (18 m radius), subplots (7.3 m radius), and microplots (2.1 m radius) where subplot centers are distanced 36.6 m horizontally from adjacent subplots. Within each plot, there are a total of four macro- and subplots. Only inventory trees with remeasurements in the national plot design were used to minimize any replication and/or measurement error. Additionally, only species with >500 individuals were included in the analysis after the filters described above were applied. The final, compiled FIA dataset included tree height, basal area, diameter, and aboveground biomass.

The growth rate and survival probability of tree species were calculated using the first and most recent inventory observation for each tree in the database. Aboveground tree growth was calculated by using the allometric carbon estimate (kilograms of carbon [C], kg C) (Jenkins et al., 2003) from the dbh of the tree and species-specific parameters. Changes in growth are determined using the first and last estimates of kg C which are then divided by the elapsed time between the measurements. The growth analysis included trees that were on private, state, and federal lands and did not take disturbance history into account during data filtering. To minimize additional error, we removed trees with growth rates beyond the 95% quantile and any trees with negative growth rates. Tree survival probability was estimated by using a binary live or dead result between the first and the last observation for each inventory tree. Trees that were harvested or were recorded as dead in both the first and the last measurements were excluded from the analysis.

Overall, 2.03 million trees were used to evaluate tree growth rates, and 2.49 million trees were used to evaluate the probability of tree mortality. Tree height, basal area, aboveground biomass, and the number of years between the first and the last FIA observation were used as predictors for model development. We evaluated tree diameter, annualized tree growth, height ratios between the inventory tree and the tallest tree, and trees per hectare (Pavlovic et al., 2023), and determined that these predictors were confounding factors with one of the following predictors: tree height, basal area, and aboveground biomass. The state of Wyoming was excluded in our model because repeat measurements were not available at the time that the database was compiled. While we only focused on 10 species here, all other local N CLs for 127 species (growth) and 140 species (survival) results were calculated using a simplified method and are detailed in Appendix S1. We selected these 10 species because (1) they had relatively large sample sizes (>2000), (2) the species represented a geographical range across multiple climatic regimes in both the eastern and western United States, and (3) the species are considered important ecologically (e.g., mammal/bird uses), culturally (e.g., sightseeing), and/or economically (e.g., used for wood products).

### Environmental conditions

In addition to environmental covariates that have been used previously (Horn et al., 2018; Pavlovic et al., 2023), we compiled additional covariates to predict tree growth rate and decadal survival probability responses. These variables were selected to reflect environmental stressors that are likely to impact these ecological responses. The new covariates included drought stress, soil characteristics, and ozone. Both previous and newly compiled factors were used as predictors within model development and iteration.

Environmental conditions at each FIA plot were assembled and included (1) monthly mean temperature and precipitation data from the Parameter-elevation Regressions on Independent Slopes Model (PRISM) (PRISM Climate Group, 2021) and (2) annual atmospheric N and sulfur deposition (TDep version 2018.01) from the U.S. National Atmospheric Deposition Program’s Total Deposition Science Committee (TDep) (NADP Program Office, 2022). In brief, PRISM is an interpolation method that reflects current spatial climate patterns at 800-m to 4-km spatial resolutions, and a monthly temporal resolution by using a weighted regression scheme to account for complex climate regimes (Daly et al., 2008). Additionally, annual N deposition (in kilograms of nitrogen per hectare per year) is modeled at an ~4-km resolution (spatial resolution differs among model versions) using a measurement-model fusion approach, which combines measured air concentrations and wet deposition with modeled deposition velocity and dry deposition data from the Community Multiscale Air Quality (CMAQ) model (Schwede & Lear, 2014). In our study, annual N and sulfur deposition was obtained for 2000 through 2019 for the ML model. We used the average N and sulfur deposition between 2000 and 2019 as a predictor within the model to estimate the annualized growth rate and survival probability of each inventory tree. Model estimates were compared with true values of growth rate and survival probability from the FIA data.

Drought severity can directly and indirectly affect forests at the individual and stand levels as well as ecosystem services such as timber production (Woodall et al., 2013) and carbon storage (Gonzalez et al., 2015). In the eastern United States, drought effects have mostly been observed at the individual tree level, while in the western United States, stand-level changes have already been observed (Clark et al., 2016; Klos et al., 2009). The Palmer Drought Severity Index (PDSI) is a standardized index that is used to estimate relative dryness on a scale of −10 (dry) to +10 (wet). Five-day PDSI values (unitless) are provided in a gridded format at 4-km spatial and 5-day temporal resolution, and were obtained for 1997 through 2020 from the GridMET program (Abatzoglou, 2013) for the conterminous United States. We obtained all available 5-day data for June, July, and August and then aggregated these values to summer (JJA) mean PDSI values, which were compiled at each FIA plot location for each year. For each inventory tree, the mean, median, maximum, and minimum JJA PDSI values were calculated across all years (1997–2020) from the first to last observation. Due to missing data in coastal locations, some trees lacked PDSI data. In these cases, missing data values were replaced by the PDSI value from the FIA plot’s nearest cell neighbor.

Tropospheric ozone concentrations have been known to impact plant species with variable outcomes, such as decreased crop yields, for the last 60 years (Karnosky et al., 2007). Tree species can experience reduced stomatal conductance and photosynthetic rates (Novak et al., 2005), reduced biomass (Lee et al., 2022), and alterations to biomass allocations in their root-shoot systems (Grantz et al., 2006). While it has been demonstrated that accumulated stomatal fluxes (e.g., phytotoxic ozone dose) are better indicators for understanding impacts to biomass and visible leaf injury than exposure concentration metrics (e.g., AOT40, W126) (Karlsson et al., 2007), the datasets needed to calculate stomatal flux accumulation are extensive, species-specific, and limited within the United States. Here, we use the annual W126 exposure concentration metric as a predictor within the ML model. Annual ozone concentrations, measured at monitoring locations across the United States, were obtained from Environmental Protection Agency (EPA)’s Air Quality System (AQS) to calculate annual W126. Annual W126 ozone values are a metric of cumulative exposure to ozone (in parts per million per hour) for the daylight hours during the summer growing season and is commonly used to evaluate ozone exposure impacts to ecosystems (Lefohn & Runeckles, 1987; U.S. Environmental Protection Agency, 2007). Briefly, the W126 value is expressed as a sum of weighted hourly concentrations over the 12-h daylight period (8:00 a.m. to 8:00 p.m.) during rolling 3-month periods across the ozone monitoring season (U.S. Environmental Protection Agency, 2022). Only air quality monitors with at least 75% data completeness were used. Interpolated ozone rasters were created using output from the Software for the Modeled Attainment Test-Community Edition (SMAT-CE). SMAT-CE interpolates annual ozone monitor data at a 4-km grid cell resolution to create surfaces of annual W126 ozone using a Voronoi Neighbor Averaging (VNA) technique at a 4-km resolution (Chen et al., 2004; Gold et al., 1997). SMAT-CE also generates files of nearest neighbor data for the VNA interpolation scheme. The nearest neighbor data include inverse distance weights squared of the monitor values at each grid cell. The inverse distance weights squared files were used to create annual ozone rasters from 2000 to 2018. The annual W126 ozone was compiled at each FIA plot, and statistics were generated over the observation period in an approach similar to that used for PDSI (i.e., mean, median, maximum, and minimum).

Soil microbial communities and characteristics are a primary driver of N assimilation in trees (Carter et al., 2017). For example, the interactive effects between N deposition and humus accumulation have been shown to result in the removal of other critical nutrients in soils which also leads to reduced decomposition of organic matter, depending on the stage of humus development (Berg & Matzner, 1997). As previously mentioned, excessive N deposition can acidify soils through the leaching of base cations (${\text{Ca}}^{2+},{\text{K}}^{+}$, and ${\text{Mg}}^{+}$). Nitrogen enrichment and acidified soils can have cascading effects on carbon accumulation in aboveground and belowground biomass pools (De Vries et al., 2006; Liu & Greaver, 2010). Notably, soil properties, such as higher clay content and the presence of Al and Fe, will lead to greater retention of carbon and other key nutrients (e.g., phosphorus) (Soong et al., 2020). Here, we compiled datasets that would have interactive effects with N deposition which could subsequently affect above-ground biomass accumulation rates and/or tree mortality. We obtained soils’ organic matter percentage, pH, and clay percentage rasters for soil layers (0–30 cm depth) from the Gridded National Soil Survey Geographic Database (gNATSGO) ArcMap toolbox (United States Department of Agriculture, 2020). These data are provided at a 30-m resolution. The time-invariant values were compiled at each FIA plot location and used as environmental predictors within the ML model.

### ML model

We developed growth and survival models for 127 and 140 tree species, respectively, using the XGBoost ML algorithm (Chen & Guestrin, 2016). In this work, we only focus on the 10 selected species for which bootstrapping was conducted. We present limited results for all tree species, using the individual condition expectation approach without bootstrapping, in Appendix S1. Since bootstrap results were omitted to limit computational requirements, these other species’ CL estimates do not provide the probabilistic results that are reported here for the 10 selected species. Briefly, we developed XGBoost models to predict annual growth rate and decadal survival probability. For each species and outcome/process (i.e., growth or survival), 600 bootstrap models were trained using a resampled dataset that was sampled randomly with replacement (Fawcett, 2006); 600 bootstraps were chosen to ensure enough replicates were modeled to minimize noise. The bootstrapped training data were used to evaluate each inventory tree for each species 600 times. Thus, each inventory tree had 600 CLs calculated to determine the error around the N CL estimate for individual trees.

Modeling was conducted using the Scikit-learn 0.23.2 and XGBoost 1.2.0 libraries in Python 3.6. We assessed growth model performance using the coefficient of determination ($R^{2}$) and survival model performance using the receiver operating characteristic’s area under the curve (AUC) (Fawcett, 2006). The full modeling approach, including mathematical description of the models, can be found in Pavlovic et al. (2023).

### CL determination

Our previous modeling approach (Pavlovic et al., 2023) used partial dependence plots to develop static, species-wide dose–response curves for N deposition impacts on tree growth and survival probability. To develop spatially explicit CLs at the individual tree level, we use a modified approach by calculating the ICE for growth and survival probability. We generated ICE results, which describe the functional relationship between a predictor variable (i.e., N deposition) and the predicted result for individual observations (Goldstein et al., 2015), for each tree in an FIA plot. To calculate the error around individual tree CLs derived from the ICE results, conditional expectation curves are plotted for each bootstrap ICE result. Each ICE curve is assessed across the range of N deposition experienced by a tree species across its entire range, at 20 equidistant bins, while holding all other predictors constant. This method allows us to understand the predicted response of growth or survival probability as a function of N deposition, while including all mediating factors for a single inventory tree. Thus, we can evaluate the error around the N CL by evaluating the 600 bootstrapped ICE responses. Hereafter, error is described as the uncertainty in the N CL estimate for a single inventory tree, based on the application of the 600 models to derive the point estimate.

Using each ICE result for each inventory tree response, we evaluated the N deposition at which a 1% decrease from the maximum growth or survival probability occurred to be consistent with methods in Pavlovic et al. (2023). While the conventional definition of the CL is the condition where no detrimental effects to an ecological factor are known to occur below that stressor amount (NADP CLAD Committee, 2017; Nilsson, 1988), there is no commonly accepted level of effect (e.g., 1% vs. 5%) on annual tree growth rates or decadal survival probability that have been defined as harmful within the CL community. We established a 1% decrease as the N CL (${\hat{\text{CL}}}_{\text{np}}$) for the bootstrap result of each individual tree using Equation (1):

(1) $$x_{s-1}+(\hat{\text{ICE}}(x_{s-1})-R\times \hat{\text{ICE}}(x_{S{\hat{\text{ICE}}}_{\text{max}}}))\;\times \frac{x_{s-1}-x_{s}}{\hat{\text{ICE}}(x_{s-1})-\hat{\text{ICE}}(x_{s})}$$

where R is the percent decrease of interest (e.g., 1% decrease is 0.99) in annual growth rate or decadal survival probability, ${\hat{\text{ICE}}}_{\text{max}}$ is the value that maximizes the ICE value, and $x_{s-1}$ is the first ICE less than $x_{s}$. The CL for each inventory tree (${\hat{\text{CL}}}_{\text{np},b}$) was calculated as the median value for ${\hat{\text{CL}}}_{\text{np}}$ across all bootstraps (i.e., local median CL) and the 95% CI width was estimated using the bootstrap results (i.e., local lower and upper 95% CI CL). We use these results to assess the spatial variability of the local median CL value and the width (range) of the local 95% CI for each inventory tree.

We also performed a sensitivity test to understand how the selection of a different decrease threshold (e.g., 5%) from the maximum affects the ${\hat{\text{CL}}}_{\text{np},b}$. Due to the lack of widely accepted target levels, we briefly report the sensitivity test results in Appendix S1 to demonstrate how an effect threshold selection can change resulting ${\hat{\text{CL}}}_{\text{np},b}$ values. We focus on the 1% decrease threshold here as an ecologically conservative approach (i.e., the most protective for tree species) and to be consistent with the methodology of Pavlovic et al. (2023).

### Mediating factor importance evaluation

SHAP analysis evaluates how influential the contributions of each predictor are to the outcome (e.g., growth rate of a tree) (Shapley, 1953; Štrumbelj & Kononenko, 2014). We used an approximation method (i.e., SHAP) to estimate Shapley values (Lundberg & Lee, 2017) for each of our models to understand variable importance in each model. In plain terms, SHAP values (unitless) provide a quantification of how much the inclusion of a mediating factor (e.g., soil pH) in the model contributes to the model prediction (e.g., difference in kilograms of carbon per year, Δ kg C year^−1^). Positive SHAP values indicate that the mediating factor resulted in increased growth rates or survival probability, while negative SHAP values mean a decreased value was predicted. Additionally, SHAP values can be compared to the value of the mediating factor on a relative scale (0–1) to understand if a higher mediating factor value (e.g., increased ozone) or lower mediating factor value leads to higher or lower outcomes (e.g., higher or lower growth rates). SHAP is similar to feature-importance analyses, and generates additive feature attributions using Equation (2):

(2) $$y_{i}={\text{shap}}_{0}+\ldots +\text{shap}(X_{ji})$$

where $y_{i}$ is the model prediction value for observation I, ${\text{shap}}_{0}$ is the mean prediction across all observations, and $\text{shap}(X_{ji})$ is the SHAP value of the jth feature for observation i, which is a quantification of the marginal contribution of the feature to the prediction. We conducted a SHAP value analysis on the test (25%) data used in the bootstrapping to understand the importance of mediating factors on the outcome of the ML model.

### Probability of CL exceedance

We obtained the most recent 3-year average (2019–2021, TDep version 2022.01) total N deposition to evaluate CL exceedances under recent air pollution conditions. To leverage the bootstrapping method of calculating N CLs, we calculated a probability of exceedance metric which used each bootstrapped CL estimation for each inventory tree, and determined whether the 2019–2021 N deposition was greater than the bootstrapped CL using simple subtraction (i.e., N deposition minus bootstrapped CL). To then calculate the probability that an inventory tree is experiencing an exceedance in its CL, we used Equation (3):

(3) $$P(A)=\frac{\sum_{i=1}^{n}E}{600}$$

where P(A) is the probability that the N CL is being exceeded, E is a binary where 1 = CL is exceeded and 0 = CL is not exceeded; this term is summed to the total number (n) of bootstraps where a CL was established. Last, 600 reflects the total number of possible CL estimations from the bootstraps. Once probabilities of exceedance were calculated for each inventory tree, probabilities were averaged at the FIA plot level, by species, to result in an average probability of exceedance at the plot level.

## RESULTS

### Model performance, species-wide N CLs, and comparisons with previous N CLs

Using the ICE ML approach, we were able to estimate the local median N CL (i.e., ${\hat{\text{CL}}}_{\text{np},b}$) for each inventory tree from the 10 selected species. The growth model performance had $R^{2}$ values ranging from 0.46 to 0.73. On average, the growth $R^{2}$ was 0.05 higher relative to prior ML modeling efforts (Pavlovic et al., 2023). The survival models resulted in AUC values ranging from 0.71 to 0.85, with modest improvement over prior ML-modeling performance (average AUC increase of 0.048). Full model performance results for each species are provided in Appendix S1: Tables S1 and S2.

N CLs were highly variable among species and had wide 95% CI ranges (typically >5 kg N ha^−1^ year^−1^) within species (i.e., species-wide; Table 1). These wide CIs represent both spatial variation in the CL and error in the CL estimation. Western hemlock (*Tsuga heterophylla*) has the lowest, species-wide median N CL for growth (4.5 kg N ha^−1^ year^−1^), while yellow-poplar (*Liriodendron tulipifera*) has the highest (13.0 kg N ha^−1^ year^−1^). Similar to growth, western hemlock is the least resilient to N in terms of survival probability (species-wide median CL = 2.8 kg N ha^−1^ year^−1^), while yellow poplar has the highest median survival N CL (11.5 kg N ha^−1^ year^−1^) (Table 1).

**FIGURE 1:**
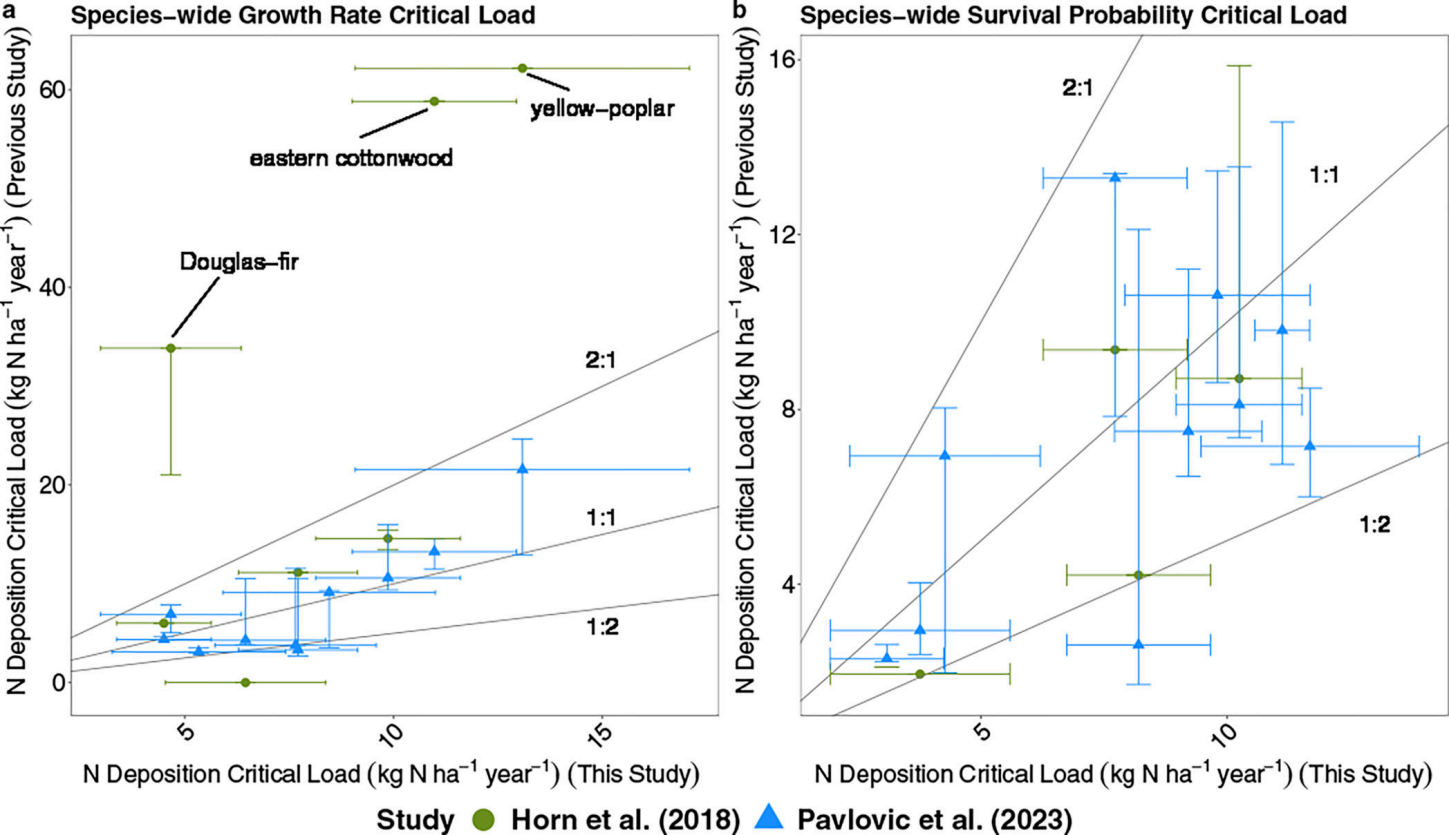
The species-wide mean nitrogen (N) critical load (CL) comparisons from our study (*x*-axis) compared to previous studies’ N CL for (a) growth rate and (b) survival probability. Our species-wide N CLs are averaged, with standard deviations, across local median N CLs. Ranges for the N CL are shown as uncertainty bounds along either the *x*- or *y*-axis for the relevant study. 1:2, 1:1, and 2:1 lines are also shown.

We also compared our growth and survival results to previous studies, which determined static, species-wide N CL nationally in the United States (Figure 1). We used our species-wide average CLs for comparisons, which were summarized from local median CLs across the bootstrap results. Our results were within ±35%, on average, when compared with the static, species-wide N CLs found in Pavlovic et al. (2023). Differences can likely be attributed to the updated FIA dataset, additional predictors, and differences between the individual ICE curve approach and the partial dependence method used in previous work (additional details are contained in Appendix S1). The estimates were also within ±34%, on average, when compared with the maximum-likelihood-derived, species-wide estimates from Horn et al. (2018) for survival probability. Most species fell between the 1:2 and the 2:1 line between our study and the aforementioned studies (Horn et al., 2018; Pavlovic et al., 2023) (Figure 1). However, there were large disparities in growth N CLs for three species (yellow poplar, eastern cottonwood [*Populus deltoides*], and Douglas-fir [*Pseudotsuga menziesii*]). For these species, Horn et al. (2018) predicted much higher N resiliency (Figure 1a), and CLs differed by as much as 47.7 kg N ha^−1^ year^−1^ from our results. Tabular comparisons of N CLs between studies are contained in Appendix S1: Table S3.

### Spatially variable N CLs

**FIGURE 2:**
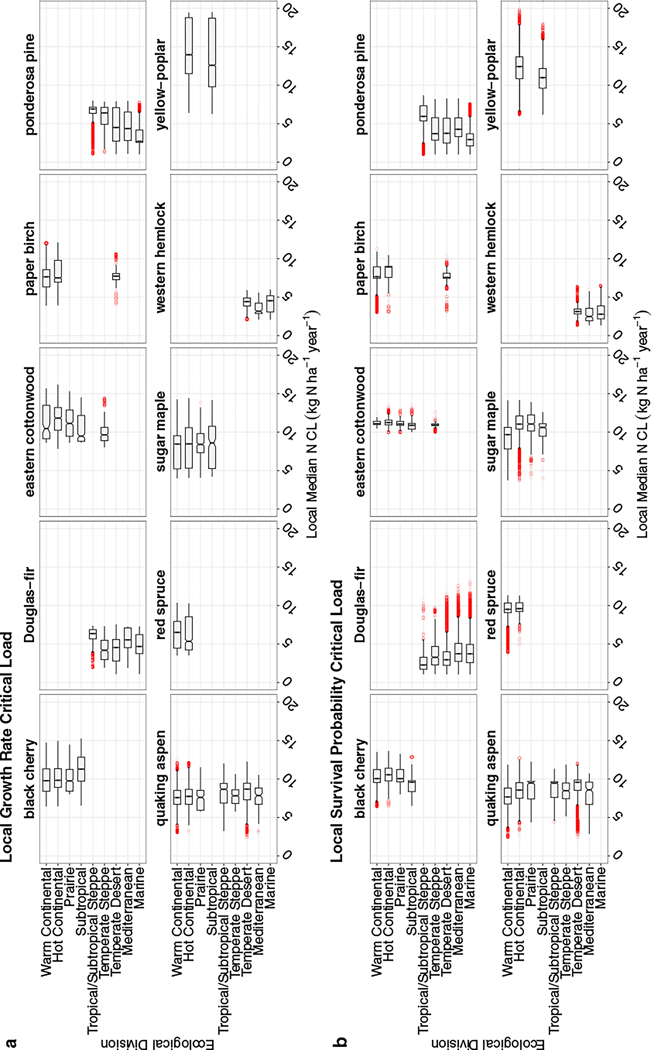
Box-and-whisker plots for each tree species’ local median nitrogen (N) critical load (CL) (in kilograms of nitrogen per hectare per year), separated by Ecological Division for (a) growth rate and (b) survival probability. The median N CL, across local median N CLs, for a given Ecological Division is denoted by the middle black line within each box, notches are used to demonstrate whether box and whiskers are significantly different, and red points are outliers.

**FIGURE 3:**
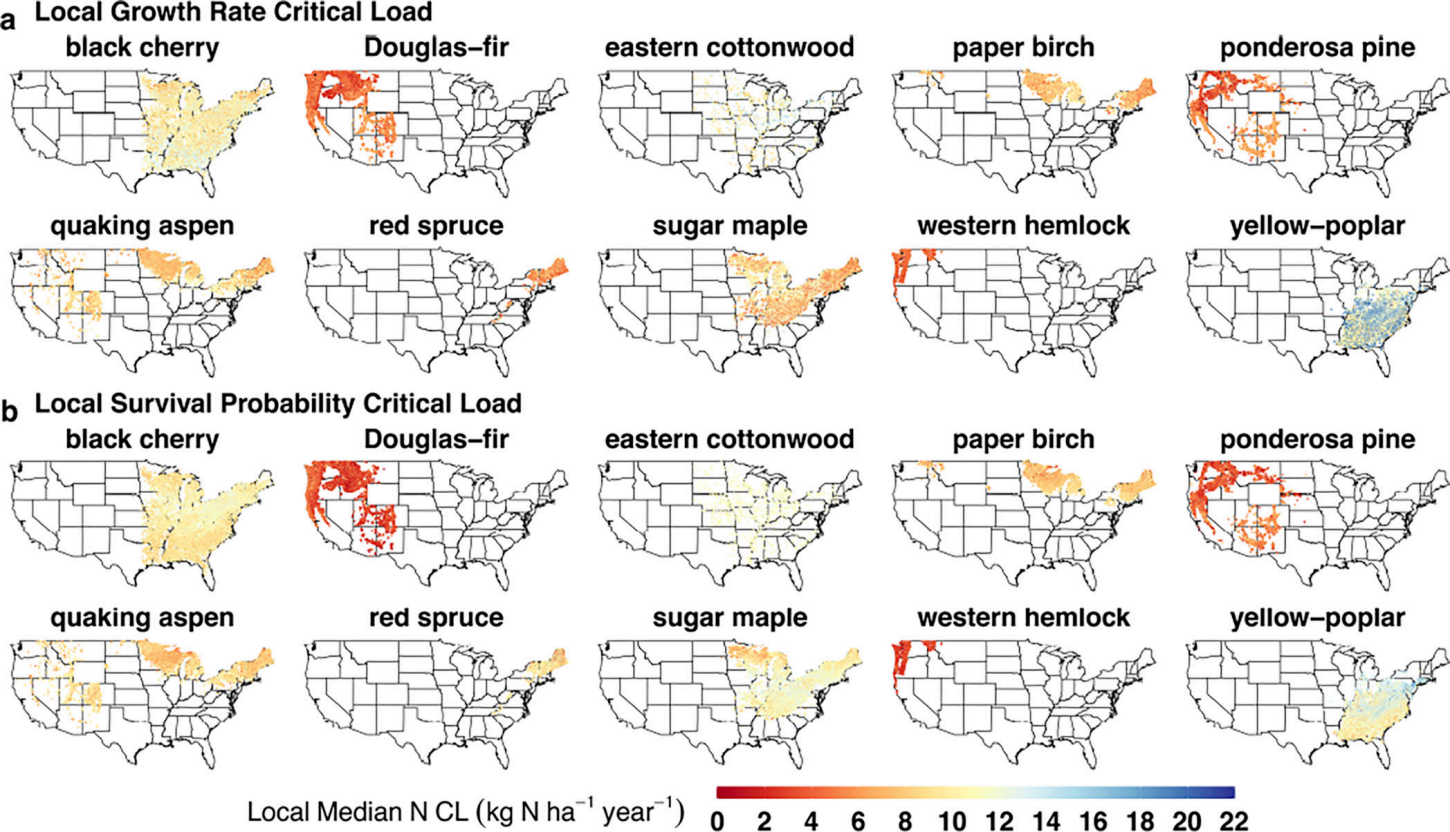
Map of local median nitrogen (N) critical loads (CLs) for (a) growth rate and (b) survival probability for the 10 selected tree species. Black cherry and ponderosa pine are discussed in the text.

We evaluated the spatial variability in N CLs for each tree species. Due to the bootstrapping method used in the XGBoost model—where 75% of training data were used with replacement—a single inventory tree will have variable N CLs produced at a single location. We refer to the variation around the CL as the error for a single inventory tree (i.e., 95% CI width) and evaluate the 600 results at the localized N CLs at the lower (2.5 percentile), median, and upper (97.5 percentile) levels. Subsequently, these local median CLs, with error, can then be summarized across larger geographic regions. We referred to the geographic range of N CLs as variability. N CLs were evaluated in two ways: (1) as distributions of local median N CLs across U.S. Forest Service Ecological Divisions (Figure 2), and (2) the CI width in N CLs (i.e., error) at an FIA plot level (Figure 3). For the first analysis, we used geographic boundaries of Ecological Divisions to evaluate regional-scale distributions. These divisions are differentiated by patterns in precipitation and temperature within ecoregion domains—thus, they can provide coarse insight into how climatic regimes may be affecting the N CL estimation. At an FIA plot level, we investigated spatial patterns for a given species to understand whether geographic patterns exist that aren’t apparent in climatic regimes (i.e., Ecological Division).

At the Ecological Division level, we find that species’ local median N CLs only slightly differ when comparing across climatic regimes, but there is considerable variation across FIA plots within Divisions (Figure 2). This suggests that variation in the CL is either driven by smaller-scale, non-climatic factors within a Division, or that the climatic variation within Divisions is sufficiently wide to drive this variation. For example, Division-level median growth rate N CLs for sugar maple (*Acer saccharum*) ranged from 8.4 to 8.6 kg N ha^−1^ year^−1^ across Divisions, but local median CLs within a single Division varied widely—such as in the Warm Continental Division where local CLs range from 4.0 to 14.3 kg N ha^−1^ year^−1^. Yellow-poplar and sugar maple tend to have the most variable local median N CLs for growth rate within Divisions (ranges of >8.5 kg N ha^−1^ year^−1^) while western hemlock and Douglas-fir are slightly less variable within Divisions (local median ranges generally <5 kg N ha^−1^ year^−1^). Additionally, some species, like ponderosa pine (*Pinus ponderosa*), show greater variability in some Divisions (e.g., Temperate Desert) but not in others (e.g., Tropical/Subtropical Steppe). For survival probability, yellow-poplar and Douglas-fir have the most variable local CLs within Divisions (ranges generally >10 kg N ha^−1^ year^−1^), while eastern cottonwood ranges are less variable (≤3.5 kg N ha^−1^ year^−1^). Although Ecological Divisions are generally separated by climatic differences, there is still considerable variation in the environmental conditions that can occur within a given Division. Thus, a more granular approach, such as comparisons at the plot level, is necessary to further understand spatial variability in N CLs.

At the FIA plot level, significant N CL variability was found for some of the species, depending on the ecological response (e.g., growth rate). For example, much of the black cherry (*Prunus serotina*) range in the southeastern United States had local median N CLs for growth between 10 and 15 kg N ha^−1^ year^−1^, while much of the northern and northeastern portion of its range had a local median CL between 5 and 10 kg N ha^−1^ year^−1^ (Figure 3a). The inverse is true for the survival N CLs of black cherry, where its southeastern range is more sensitive, and northern inventory trees are more resilient to N deposition (Figure 3b). For a western species, the local growth rate CLs of ponderosa pine are more N-tolerant in southwestern states (5–7.5 kg N ha^−1^ year^−1^), while trees in the Pacific Northwest have more sensitive local N CLs (<5 kg N ha^−1^ year^−1^). The survival N CLs for ponderosa pine have a similar geographic pattern that falls within the same N deposition ranges as well. Generally, spatial patterns represent gradual gradients in local N CLs rather than abrupt transitions. While there are no discernible, systematic patterns across species, there are geographic patterns within species that indicate local mediating factors may be influencing N sensitivity for trees of the same species.

**FIGURE 4:**
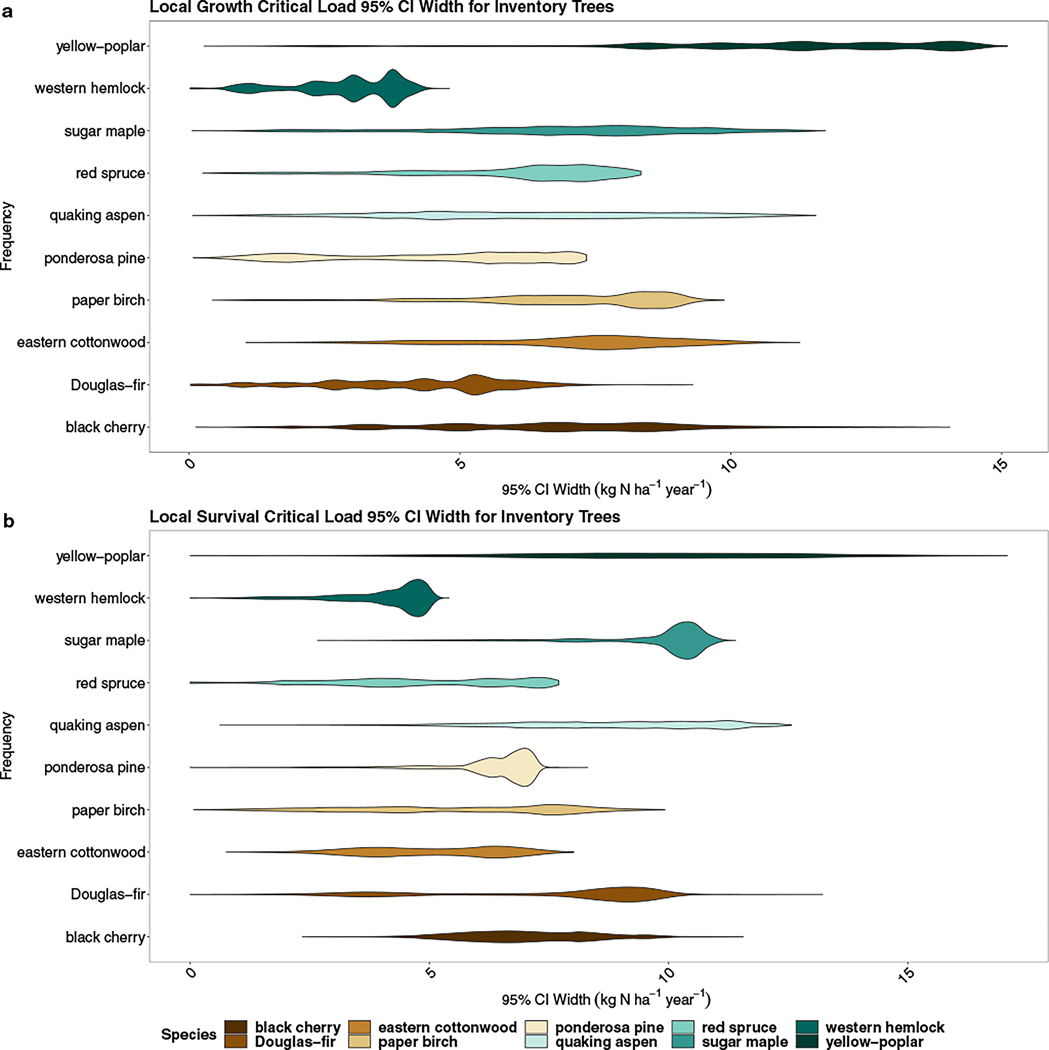
Distribution of the 95% CI width for the 10 selected species across all inventory trees for (a) growth rate nitrogen (N) critical loads (CLs) and (b) survival probability N CLs. The 95% CI width for each inventory tree is estimated by evaluating the 600 bootstrap estimates from the independent conditional expectation and determining the range between the 2.5% and 97.5% percentiles. The width of the violin relays the density of points where the wider a violin is at a given point, the higher number of points that fall in that value range.

To understand the potential significance of error observed in our results, we assessed the 95% CI width of the individual tree predictions for each species. We find considerable error in the calculated N CL for individual trees for both growth and survival across bootstraps (Figure 4). For the 10 species we evaluated, the growth rate CL 95% CI ranged from less than 0.02 to as high as 15.1 kg N ha^−1^ year^−1^ for individual trees. Some species have a normally distributed 95% CI range distribution (black cherry, quaking aspen [*Populus tremuloides*]), others are slightly left-skewed (eastern cottonwood, sugar maple), and a few were heavily left-tailed (paper birch [*Betula papyrifera*], red spruce [*Picea rubens*], yellow-poplar, western hemlock) (Figure 4). The distribution of 95% CI growth rate CL ranges for ponderosa pine is bimodal. Yellow-poplar has the highest 95% CI range at 11.3 kg N ha^−1^ year^−1^. For survival probability, the 95% CI width spans from 0.0 to 17.1 kg N ha^−1^ year^−1^. Like growth rates, there are variable distributions across species with two species having normal distributions (black cherry, yellow-poplar) while the remainder are left-skewed (Figure 4). Ponderosa pine, sugar maple, and western hemlock are heavily left-tailed.

### Mediating factor influences on growth rate and survival probability predictions

To better understand the magnitude of mediating factor influences on tree sensitivity to N deposition, we used SHAP analyses to quantify the relative importance of a factor on growth and survival. Note that these mediating factors should not be interpreted as the most important features directly affecting the actual determination of a CL because the ICE approach uses a 1% decrease to establish the CL. Rather, the results of the SHAP analyses provide insight into the determination of which mediating factor used in the model was the most important for both higher and lower predictions of growth or survival probability. In other words, these results reflect what mediating factor had the greatest impact on improving the XGBoost model and are therefore a correlative proxy for which physical or environmental mechanisms may be the most important for determining CLs, but they may not always directly align with ecological principles. Moreover, caution should be taken in interpreting the SHAP results as direct causalities since multi-collinearity issues are present for some species such as red spruce and ponderosa pine (Appendix S1: Table S6), but a correlative understanding can be gleaned.

Across the 10 species, growth rate predictions were most heavily influenced (i.e., high and low SHAP values) by the initial aboveground biomass estimation of the inventory tree and other competition terms (i.e., basal area within the FIA plot and basal area of trees larger than the tree of interest in the plot). When high SHAP values were found (e.g., higher growth rates), there was typically a higher mediating factor value as well (i.e., higher initial aboveground biomass). This translates to inventory trees which initially had a higher above-ground biomass tended to have higher growth rates between the first and last observations. This pattern was evident across all 10 species. Beyond aboveground biomass and competition terms, SHAP analysis results for growth rate predictions were variable among species. For example, higher precipitation tended to lead to higher predictions in growth for most species but not for yellow-poplar, which experienced lower growth rates (i.e., negative SHAP values) with increased precipitation. Notably, soil conditions tended to be the least impactful feature—absolute median SHAP values were within 0.07 across all species (Table 2)—on the growth rate predictions. Correlations between soil conditions and other mediating factors were generally significant but weakly correlated (<±0.4) for most species (Appendix S1: Tables S4 and S5). This lack of impact from soil conditions was evident for almost all species except for the growth rate predictions of eastern cottonwood, which tended to have higher influences from percent organic matter than other species. Additionally, some ozone-sensitive species (e.g., quaking aspen; Karnosky et al., 2003) experienced decreased growth rates (SHAP < 0) with increased W126 values, while others (e.g., black cherry) experienced increased growth rates with higher W126.

**FIGURE 5:**
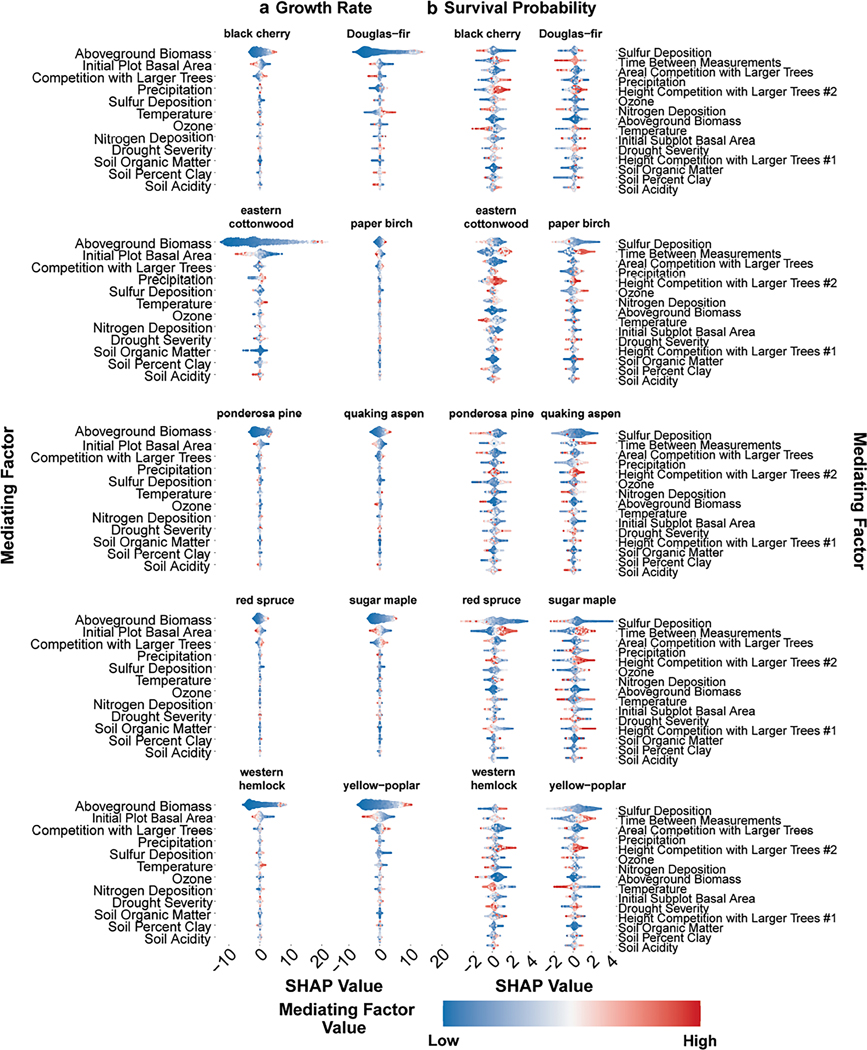
Shapley approximation (SHapley Additive exPlanations [SHAP]) value results for all 10 species for both (a) growth rate and (b) survival probability XGBoost model predictions. Each mediating factor used in the modeling is shown as a separate *y*-axis bin. Positive SHAP values mean the mediating factor resulted in an increased growth rate or survival probability while negative SHAP values mean a lower value was predicted. The color gradient for the legend (i.e., mediating factor value) illustrates whether the mediating factor value was relatively low or high as compared against all values of the respective mediating factor for each tree species. Each point represents a single inventory tree. Mediating factors are ordered by their absolute median SHAP value. Individual SHAP figures for all species are included in Appendix S1.

Competition terms, climate conditions, and sulfur deposition were the most important mediating factors of survival probability (Figure 5; Appendix S1: Figures S32–S41). If competition term values were higher, this tended to lead to positive SHAP values, or increased survival probability. For example, if the initial height ratio of the inventory tree of interest to the tallest tree in the subplot was high (i.e., Height Competition with Larger Trees no. 2 in Figure 5), then a higher predicted survival probability was generally observed across most species, likely reflecting the inventory tree’s ability to compete for light. Increased sulfur deposition mostly led to decreased survival probability, except for western hemlock and Douglas-fir which generally had higher survival probability with increasing sulfur deposition. For most species, temperature and precipitation levels had a significant negative impact on the model predictions in both directions (i.e., positive or negative, depending on species). SHAP value magnitudes and directionality for these climatic mediating factors were variable though. Some species, such as yellow-poplar, eastern cottonwood, western hemlock, and black cherry, have negative SHAP values with increasing temperature, while the remaining species have higher predicted survival probability with elevated temperatures. Generally, precipitation tended to be more impactful to growth rates and survival probability (i.e., higher absolute median SHAP values) than temperature (Table 2). Ozone had moderate impacts to modeled mortality predictions with bifurcating effects (both positive and negative SHAP values with increasing ozone). Soil conditions generally tended to have the least amount of impact—absolute median SHAP values were within 0.17 across all species—on the model predictions of survival probability (Figure 5, Table 2; Appendix S1: Figures S32–S41). Individual SHAP value results are contained in Appendix S1: Figures S32–S41.

## DISCUSSION

### What drives the spatial variability in tree growth and survival?

While we are not able to directly quantify the influences from mediating factors on the predicted N CL, we are able to understand which mediating factors are the most influential for predicting growth rate and survival probability of inventory trees. The resulting SHAP values across all species for each mediating factor are detailed in Table 2. Here, absolute median SHAP values inform which mediating factors had the largest impact on the ML model’s predictions in both the positive and negative directions but should only be compared in relation to other mediating factors. Mediating factor values reflect the relative value of the mediating factor at an FIA plot (e.g., ozone concentration) against all other values for that species across FIA plots.

Mechanistically, soil conditions, including pH, organic matter, and permeability (percent clay), are some of the best understood mediating factors in relation to how N deposition affects tree species. In theory, soil pH serves as a proxy for soil acidification in our study and should be reflective of the cascading effects from potentially toxic cation mobilization such as aluminum and manganese (Carter et al., 2017). Our results indicate that soil acidity (as represented by soil pH) was generally one of the least impactful factors for predicted growth rate and survival probability of tree species; this is demonstrated by the fact that soil pH had the lowest absolute median SHAP value (within ±0.05 for growth rate and within ±0.12 for survival probability) across all mediating factors (Table 2). Similarly, percent clay and percent organic matter were also minimally impactful (Table 2). While this finding is somewhat counter-intuitive from a physiological perspective, it could be due to limitations of the soil characteristic data. For example, soil measurements are taken at a shallow measurement depth (30 cm) and mature stand tree roots will be much deeper (>60 cm); this could result in lower effects to the modeled predictions. Additionally, other soil metrics (e.g., soil base saturation, exchangeable calcium), which are not directly available across the full FIA panel, could provide more predictive capability for pH as these will have a direct effect on the acid–base status or calcium status of soils (Robin-Abbott & Pardo, 2017; Sullivan et al., 2013). Generally, soil conditions were minimally correlated (<±0.4) with other mediating factors so the limitation of our selected soil metrics is likely attributable to dataset constraints rather than multi-collinearity issues with other predictors.

Conversely, we find that competition, air pollution (i.e., ozone), atmospheric deposition (e.g., sulfur deposition), and climatic variables are some of the most impactful predictors for growth rate and survival probability, although the impact of these conditions varies depending on the species. Across all species’ SHAP results, we find that aboveground biomass has the larger influence on growth rate predictions (absolute median SHAP value of 1.81). Notably, N deposition tends to be moderately impactful to both growth rate and survival probability predictions when compared to other environmental and competition terms. This pattern suggests that while N deposition is not the most impactful predictor of tree outcomes, it is an important exogenous factor that can influence growth rate and survival probability for tree species. While the SHAP analysis provides a unique capability to understand what influences the modeled predictions, it cannot disentangle multi-collinearity issues. For example, the SHAP results for growth of red spruce suggest that increased ozone concentrations positively impact expected growth. However, N deposition and mean ozone concentrations are positively correlated for red spruce (R=0.77, p<0.001; Appendix S1: Table S4), indicating that increased growth rates may be due to N deposition rather than ozone. However, it is notable that previous seedling studies with experimentally applied ozone concentrations found negligible ozone effects on the growth of red spruce, so detrimental impacts are not necessarily expected for this species (Kohut et al., 1990; Laurence et al., 1989). Correlation tables for all predictors are contained in Appendix S1: Tables S4 and S5.

One advantage of our modeling approach is the ability to quantify predictor importance on model results. Previous studies have used Akaike information criterion (AIC) to determine which predictor inclusions within models will lead to the most accurate outcome in a maximum-likelihood approach (Horn et al., 2018; Thomas et al., 2010). AIC is used to compare different model outcomes to determine the best fit to the observed data. Here, we provide an alternative framework (SHAP analysis) for evaluating the importance of different environmental stressors that can be used within ML frameworks while retaining all predictors. Each method has its own strengths and limitations for determining N CLs and could be used together as ensembles when evaluating whether areas are in exceedance.

### Spatially varying exceedances in N CLs

Typically, CLs are evaluated in the form of exceedances where the threshold is subtracted from the stressor value—in this case, N deposition. Previous studies have used percentiles in the context of CLs to evaluate 95% CI widths of the CL across species (Pavlovic et al., 2023; Simkin et al., 2016), as well as exceedances across ecosystem components, such as trees or lichens (Clark et al., 2018). Here, we evaluate the most recently available 3-year average N deposition (2019–2021) across species’ ranges using a probability of exceedance metric. This metric allows us to evaluate all 600 bootstrapped N CL estimates at the inventory tree level, resulting in a probability ranging from 0% to 100%. If an inventory tree experiences a probability higher than 50%, then we assume that the N CL is exceeded at that location. We averaged probabilities, by species, at the plot level to aggregate our results. Although we use 50% here as the probability threshold, different thresholds may be more suitable depending on the resource management need. We use 50% here as a conservative demonstration of the approach, but a previous effort used the 95% CI width to determine whether an exceedance was occurring or not (Pavlovic et al., 2023).

**FIGURE 6:**
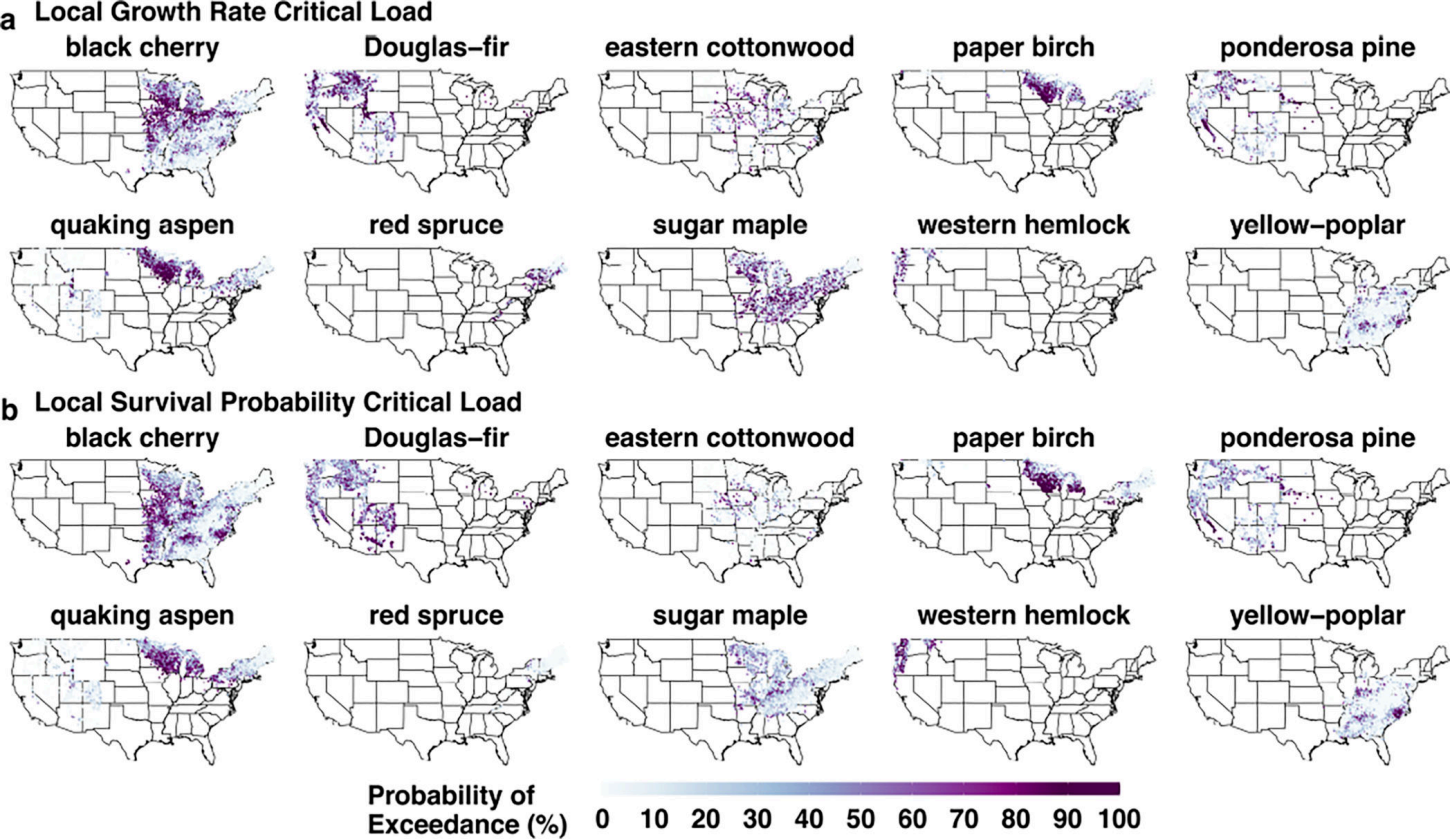
Maps of the probability of exceedance (in percentage) of the nitrogen (N) critical load (CL) for (a) growth rate and (b) survival probability when compared to 2019–2021 average N deposition for all 10 species. If the probability is higher than 50%, then there is a high likelihood that an N CL exceedance is occurring at the Forest Inventory and Analysis plot.

**FIGURE 7:**
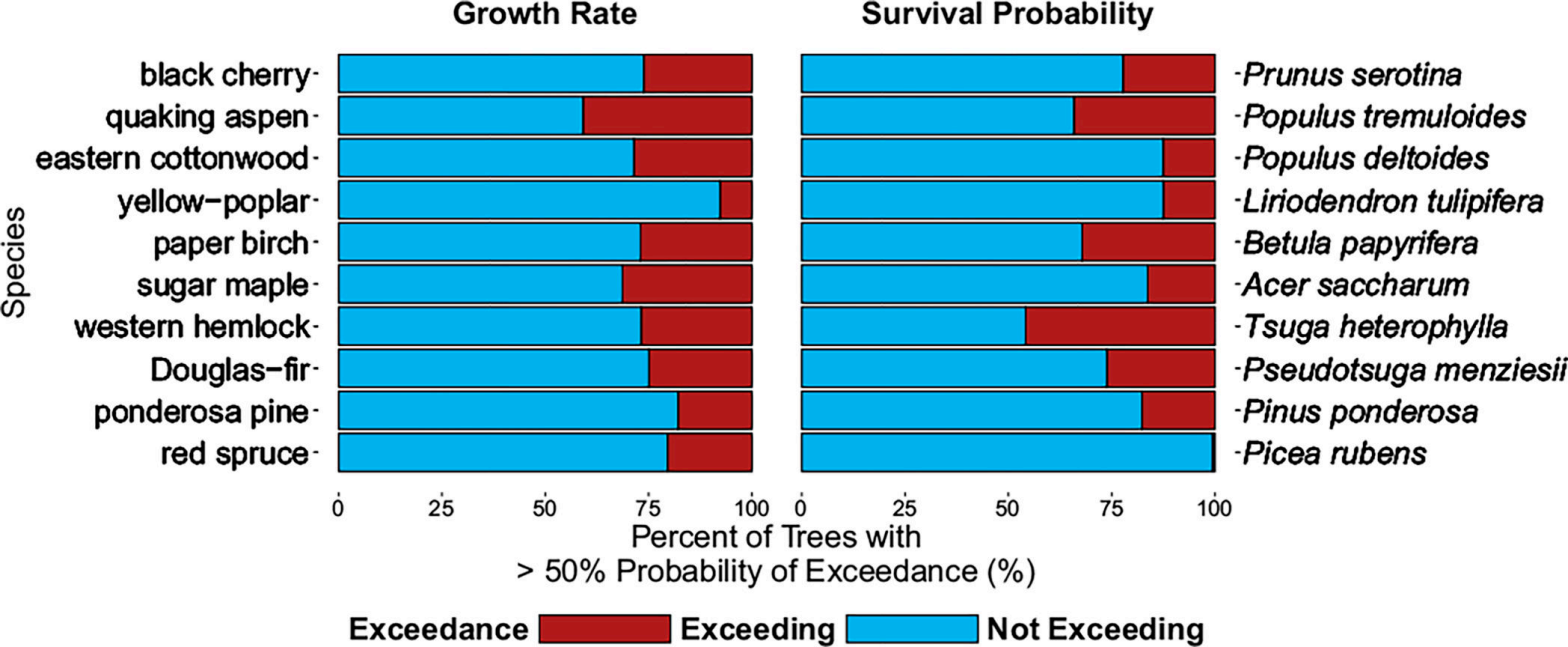
The percentage of Forest Inventory and Analysis plots in exceedance of the growth rate or survival nitrogen (N) critical loads (CLs) based on 2019–2021 N deposition levels for the 10 different species. An inventory tree was determined to be in exceedance of its N CL if there was a >50% probability of exceedance.

Using the probability of exceedance metric, we find that there are large areas of species’ ranges that have a >50% probability that their N CL is being exceeded, when compared to 2019–2021 N deposition levels. For example, when evaluating exceedances of the growth rate N CL for black cherry, 26.2% of FIA plots are experiencing exceedances across much of the Midwestern United States and portions of the Eastern Seaboard (Figure 6). Additionally, 22.3% of black cherry trees in FIA plots are experiencing exceedances of their survival probability N CL (Figure 6). Many of these exceedances occur along the eastern side of the Mississippi River Basin where agricultural activities result in higher Nr deposition and in the North Carolina region where Nr deposition is high due to concentrated animal feeding operations (Chen et al., 2020). Some species (e.g., paper birch) have similar spatial distributions in probabilities of exceedance for growth rate and survival probability, while others (e.g., sugar maple) have a greater number of high probability exceedance locations for one ecological response (e.g., survival probability) but not the other. These differences tend to reflect that species have higher survival probability N CLs than growth N CLs. Across all evaluated species, the percentage of trees among species with probabilities of exceedance >50% ranged from 0.6% to 45.8% (Table 3, Figure 7). Across its distributional range, red spruce had the lowest percentage of N CL exceedances for survival probability (0.6%), but it also has higher percentages (20.4%) for exceedances for growth. Western hemlock had the highest percentage of trees exceeding its local N CL for survival probability (45.8%) and a fairly high percentage of growth rate CL exceedances as well (26.8%). For growth, the median across all species was 26.5% of trees experiencing an exceedance; for survival probability, there was a median of 20% across all species. All 10 species and their respective percentages of N CL exceedances across FIA plots are detailed in Table 3 and Figure 7.

We also compared the probability of exceedance maps against exceedance maps from static, species-wide CL exceedances in Pavlovic et al. (2023). The purpose of this analysis was to understand whether more granular information from spatially varying N CLs results in greater utility in the context of CL exceedances. Our spatially variable results show local areas where high probabilities of exceedances occur which were not apparent in static, species-wide CL exceedance maps. For some species, there are also broad regional or species’ range-wide differences. For example, previous static results for yellow-poplar growth rate CLs showed limited exceedance across its entire range. In contrast, our spatially variable results show higher probabilities of exceedance in areas such as central Tennessee. These differences further illustrate the advantages of a spatially variable approach by providing locally tuned information on the sensitivity of tree species.

Overall, our probability of exceedance metric can provide additional context to federal land managers about which areas of the United States may need more focused protective measures. For instance, if a particular species of interest has a high probability of exceedance (>75%), then there is more confidence that an exceedance is occurring. This pattern suggests that additional pollution reduction measures may be warranted in these areas to decrease N deposition. Our results indicate that ML approaches with bootstrapping provide a unique capability to determine spatially varying N CLs for these types of efforts.

### Future evaluations of tree species’ N CLs

While our study expanded upon previous work on N CLs for tree species, several mediating factors were not incorporated into our model. Mycorrhizal associations are known to be crucially important for N uptake at tree root systems where arbuscular mycorrhizal-associated trees tend to outcompete ectomycorrhizal-associated trees as a result of differences in N acquisition process (Averill et al., 2018; Jo et al., 2019; Liese et al., 2018). While our study does not account for these associations, future inclusion of this mediating factor could have substantial impacts on growth. Similarly, carbon dioxide (CO_2_) has long been demonstrated to have an important influence on the primary productivity of trees (Idso & Kimball, 1993; Rogers et al., 1983), notably for younger stands (Kallarackal & Roby, 2012), although equivocal results have been found for mature stands (Girardin et al., 2016). Lastly, newly developed aboveground biomass estimation techniques by the U.S. Forest Service (Westfall et al., 2024) could provide more robust estimations of annual growth rates.

For survival probability, pests and wildfires are two critical stressors that have not been extensively incorporated into the modeling of tree mortality, at least in relation to air pollution. The inclusion of these mediating factors could have a large effect on the empirical determination of survival probability CLs. Additionally, with increasing repeat measurements since the FIA program became standardized in 1998, future research could also investigate time-series responsiveness of growth and survival rather than just the first and last observation. Furthermore, incorporation of other important factors, such as recruitment of new trees, should be further evaluated to understand the net gain or loss of tree species in forested areas, such as shifts in species occurrence and introductions of invasive species. As paradigms of CLs shift toward climatic stressors, inclusion of this key factor will be paramount. In addition to these ecological factors for improvement, statistical factors may also be improved. In particular, the ML approach allows for flexible relationships, which may not adhere to ecological expectations. Incorporating constraints of monotonicity or higher-order relationships could improve this area.

## CONCLUSION

Our study demonstrates how ML methods can be expanded to predict spatially varying N CLs of growth rate and survival probability for individual tree species within the contiguous United States. This enhancement offers a methodology for quantifying N CLs at more localized scales. Previous methodologies have calculated the range and uncertainty in tree species’ N CLs at national scales, but our modified XGBoost approach provides a method to empirically determine CLs at an FIA plot level. In this study, we found large error in N CLs for single inventory trees using a 95% CI width, although some of the observed error is inherent to our methodology. We also found significant spatial variability in N CLs across species’ ranges, demonstrating that environmental conditions influence species’ sensitivity to N deposition. Competition, climatic, and other air pollutant mediating factors tended to be the most influential predictors for growth rate models. The soil conditions used here (pH, percent organic matter, and percent clay) had the least amount of influence on the CL predictions for growth rates. Similar results were found for tree mortality predictions, although different competition terms were used. The impact, or lack thereof, of these mediating factors on our ML modeling could be due to differences in measurement frequency and root depth. Further investigation into how to better utilize soil datasets (e.g., base cation weathering) for these dose–response evaluations is warranted. When we examined probabilities of exceedance across the species of interest, a substantial number of plots in the United States remain in exceedance (>50% probability) for their local N CLs. This pattern suggests that uncertainties around local N CLs can be robustly incorporated into ML models evaluating deposition exceedances. Furthermore, this metric can provide useful information to local resource managers addressing local and regional air pollution emissions sources.

The Clean Air Act directs the U.S. EPA to promulgate secondary National Ambient Air Quality Standards to protect the public welfare from any known or anticipated adverse effects from the presence of air pollutants. While national air pollutant standards are necessary for the enactment of the Clean Air Act, individual trees of a given species have variable sensitivity to air pollution due to mediating factors. The use of spatially variable results can provide a more holistic understanding of ecosystem sensitivity when developing regulatory policy.

## Supplementary Material

Supplement1

## Figures and Tables

**TABLE 1:** Species-level summary of nitrogen (N) critical loads (CLs) for the 10 selected species for growth rate and survival probability

			Species-wide growth N CL (kg N ha^−1^ year^−1^)				Species-wide survival probability N CL (kg N ha^−1^ year^−1^)			
				
FIA code	Common name	Scientific name	Median	Minimum local 95% CI	Minimum local 95% CI	*n*	Median	Minimum lower 95% CI	Maximum upper 95% CI	*n*
97	Red spruce	*Picea rubens*	6.4	3.6	10.6	16,101	9.5	6.1	11.1	19,130
122	Ponderosa pine	*Pinus ponderosa*	5.4	1.9	7.7	51,208	3.8	1.3	8.2	65,594
202	Douglas-fir	*Pseudotsuga menziesii*	4.7	2.1	7.2	99,281	3.3	1.7	10.4	125,712
263	Western	*Tsuga heterophylla hemlock*	4.5	2.3	5.8	21,963	2.8	1.6	6.3	27,352
318	Sugar maple	*Acer saccharum*	8.5	4.1	11.9	75,524	10.2	4.1	14.5	90,639
375	Paper birch	*Betula papyrifera*	7.6	4.1	12.2	23,943	7.7	3.3	10.4	33,016
621	Yellow-poplar	*Liriodendron tulipifera*	13.0	9.1	20.6	35,051	11.5	8.4	19.1	41,754
742	Eastern	*Populus deltoides cottonwood*	11.0	8.4	15.9	2439	11.1	8.0	13.8	2926
746	Quaking aspen	*Populus tremuloides*	7.7	5.3	11.3	64,253	8.4	3.1	13.1	83,843
762	Black cherry	*Prunus serotina*	9.9	8.0	14.7	27,217	10.1	7.2	14.6	33,942

*Note*: The species-wide median, minimum lower 95% CI, and maximum upper 95% CI, and sample size (*n*) are shown.

Abbreviation: FIA, Forest Inventory and Analysis.

**TABLE 2:** Species-level summary of SHapley Additive exPlanations (SHAP) values across all 10 species for growth rate and survival probability.

Predictor	Description	Minimum SHAP value	Median SHAP value	Absolute median SHAP value	Average SHAP value	Max SHAP value
Growth rate						
Aboveground biomass	Jenkins calculated above ground biomass (kg C)	−12.61	−0.28	1.81	0.08	21.77
Initial plot basal area	The initial subplot basal area (m^2^ ha^−1^)	−7.77	−0.01	0.48	0.01	7.04
Competition with larger trees	The initial basal area of the subplot for trees that were larger than the tree of interest	−3.62	−0.05	0.29	−0.05	3.38
Precipitation	Mean annual precipitation (dm)	−3.70	0.00	0.12	0.02	2.31
Sulfur deposition	Mean annual sulfur deposition (kg S ha^−1^ year^−1^)	−2.25	−0.04	0.12	−0.01	3.67
Temperature	Mean annual temperature (K)	−4.92	0.00	0.12	−0.02	4.95
Ozone	Ozone W126 exposure concentration (ppm h^−1^)	−2.80	0.01	0.11	−0.01	2.40
Nitrogen deposition	Mean annual nitrogen deposition (kg N ha^−1^ year^−1^)	−1.92	0.00	0.07	0.00	1.72
Drought severity	Mean JJA PDSI	−2.45	0.00	0.07	0.00	2.47
Soil organic matter	Percent organic matter	−5.33	0.01	0.06	0.00	2.23
Soil percent clay	Percent clay	−1.93	0.00	0.05	0.00	2.18
Soil acidity	pH	−2.26	0.00	0.05	0.00	1.10
Survival probability Sulfur deposition						
Sulfur deposition	Mean annual sulfur deposition (kg S ha^−1^ year^−1^)	−3.33	0.26	0.38	0.29	4.21
Time between measurements	Elapsed time to the day (years)	−2.34	0.22	0.31	0.27	2.57
Areal competition with larger trees	The ratio of the basal area of the tree of interest to the basal area of the plot	−1.70	0.28	0.31	0.27	1.89
Precipitation	Mean annual precipitation (dm)	−1.95	0.23	0.28	0.21	1.89
Height competition with larger trees no. 2	The initial ratio of the height of the tree to the tallest tree in the subplot	−2.12	0.20	0.27	0.23	2.45
Ozone	Ozone W126 exposure concentration (ppm h^−1^)	−2.14	0.20	0.25	0.20	1.94
Nitrogen deposition	Mean annual nitrogen deposition (kg N ha^−1^ year^−1^)	−1.71	0.20	0.24	0.19	1.95
Aboveground biomass	The initial Jenkins calculated aboveground biomass (kg C)	−1.81	0.20	0.23	0.21	1.65
Temperature	Mean annual temperature (K)	−2.11	0.18	0.23	0.21	2.83
Initial subplot basal area	The initial subplot basal area (m^2^ ha^−1^)	−1.17	0.19	0.21	0.21	2.01
Drought severity	Mean JJA PDSI	−1.84	0.18	0.20	0.19	1.89
Height competition with larger trees no. 1	The height of the tallest tree in the subplot (m)	−1.19	0.14	0.17	0.16	2.37
Soil organic matter	Percent organic matter	−1.21	0.14	0.16	0.14	1.17
Soil percent clay	Percent clay	−2.01	0.13	0.15	0.14	1.41
Soil acidity	pH	−0.96	0.09	0.11	0.09	1.59

*Note*: The species-wide minimum, median, absolute median, average, and maximum are shown. SHAP values are unitless and reflect the impact on predictions of growth rate and survival probability where a larger absolute SHAP value reflects a larger impact, either positive or negative, on the resulting prediction.

Mediating factors are ordered by the absolute median SHAP value, aggregated across all species, in a descending order.

Abbreviations: JJA, June, July, and August; PDSI, Palmer Drought Severity Index.

**TABLE 3:** Species-level summary of the probability of exceedance metric across all trees for each species in Forest Inventory and Analysis (FIA) plots.

		Growth		Survival probability	
			
FIA code	Common name	Total count	Percentage of trees with high N CL exceedance probability (%)	Total count	Percentage of trees with high N CL exceedance probability (%)
97	Red spruce	16,101	20.4	19,130	0.6
122	Ponderosa pine	51,208	17.9	65,594	17.7
202	Douglas-fir	99,281	24.9	125,712	26.2
263	Western hemlock	21,963	26.8	27,352	45.8
318	Sugar maple	75,524	31.3	90,639	16.3
375	Paper birch	23,943	27.0	33,016	32.2
621	Yellow-poplar	35,051	7.7	41,754	12.5
742	Eastern cottonwood	2439	28.6	2926	12.6
746	Quaking aspen	64,253	40.9	83,843	34.1
762	Black cherry	27,217	26.2	33,942	22.3

*Note*: The total count reflects the sample size of the tree species in FIA plots that were used in the growth rate and survival probability modeling. The percentage of trees with high N CL exceedance probability (in percentage) reflects the tally of trees, for each species, that had a >50% probability that the N CL was being exceeded, when compared to 2019–2021 average N deposition levels. Scientific names are provided in Table 1.

Abbreviations: CLs, critical loads; N, nitrogen.

## Data Availability

Data (Coughlin, 2024a, 2024b, 2024c, 2024d, 2024e) are available from Figshare data repository: https://doi.org/10.6084/m9.figshare.25976689.v1; https://doi.org/10.6084/m9.figshare.25976215.v1; https://doi.org/10.6084/m9.figshare.25976182.v1; https://doi.org/10.6084/m9.figshare.25976155.v1; https://doi.org/10.6084/m9.figshare.25976074.v1. Plot-level critical load results for all evaluated species (Appendix S1: Table S7; All Species N Critical Loads; Coughlin, 2023), without bootstrap modeling, are available from Figshare: https://doi.org/10.6084/m9.figshare.22692964.v1. Other data sets and results used in this research are as follows: Previous nitrogen critical load data for tree species (Horn et al., 2018) are available at https://doi.org/10.1371/journal.pone.0205296 and (Pavlovic et al., 2023) at https://doi.org/10.1016/j.scitotenv.2022.159252 (Appendix S1).

## References

[R1] Abatzoglou JT 2013. “Development of Gridded Surface Meteorological Data for Ecological Applications and Modelling.” International Journal of Climatology 33: 121–131.

[R2] Averill C, Dietze MC, and Bhatnagar JM. 2018. “Continental-Scale Nitrogen Pollution Is Shifting Forest Mycorrhizal Associations and Soil Carbon Stocks.” Global Change Biology 24: 4544–53. 30051940 10.1111/gcb.14368

[R3] Beachley GM, Rogers CM, Lavery TF, Walker JT, and Puchalski MA. 2019. “Long-Term Trends in Reactive Nitrogen Deposition in the United States.” Environmental Manager: 1–8. https://airandwmapa.sharepoint.com/sites/AWMA_Website/Shared%20Documents/Forms/AllItems.aspx?id=%2Fsites%2FAWMA%5FWebsite%2FShared%20Documents%2Fem%2Ddo%20not%20delete%2F2019%2F7%2Femjuly19%2Epdf&parent=%2Fsites%2FAWMA%5FWebsite%2FShared%20Documents%2Fem%2Ddo%20not%20delete%2F2019%2F7&p=true&ga=1. PMC7784191 33408454

[R4] Benish SE, Bash JO, Foley KM, Appel KW, Hogrefe C, Gilliam R, and Pouliot G. 2022. “Long-Term Regional Trends of Nitrogen and Sulfur Deposition in the United States from 2002 to 2017.” Atmospheric Chemistry and Physics 22: 12749–67. 10.5194/acp-22-12749-2022 PMC11864274 40012769

[R5] Berg B, and Matzner E. 1997. “Effect of N Deposition on Decomposition of Plant Litter and Soil Organic Matter in Forest Systems.” Environmental Reviews 5: 1–25.

[R6] Blett TF, Lynch JA, Pardo LH, Huber C, Haeuber R, and Pouyat R. 2014. “FOCUS: A Pilot Study for National-Scale Critical Loads Development in the United States.” Environmental Science and Policy 38: 225–236.

[R7] Bobbink R., Hicks K, Galloway J, Spranger T, Alkemade R, Ashmore M, Bustamante M, . 2010. “Global Assessment of Nitrogen Deposition Effects on Terrestrial Plant Diversity: A Synthesis.” Ecological Applications 20: 30–59. 20349829 10.1890/08-1140.1

[R8] Bowman WD, Cleveland CC, Halada Ĺ, Hreško J, and Baron JS. 2008. “Negative Impact of Nitrogen Deposition on Soil Buffering Capacity.” Nature Geoscience 1: 767–770.

[R9] Butler TJ, Likens GE, Vermeylen FM, and Stunder BJB. 2003. “The Relation between NOx Emissions and Precipitation ${{\text{NO}}_{3}}^{-}$ in the Eastern USA.” Atmospheric Environment 37: 2093–2104.

[R10] Butler TJ, Likens GE, Vermeylen FM, and Stunder BJB. 2005. “The Impact of Changing Nitrogen Oxide Emissions on Wet and Dry Nitrogen Deposition in the Northeastern USA.” Atmospheric Environment 39: 4851–62.

[R11] Butler TJ, Vermeylen F, Lehmann CM, Likens GE, and Puchalski M. 2016. “Increasing Ammonia Concentration Trends in Large Regions of the USA Derived from the NADP/AMoN Network.” Atmospheric Environment 146: 132–140.

[R12] Butler TJ, Vermeylen FM, Rury M, Likens GE, Lee B, Bowker GE, and McCluney L. 2011. “Response of Ozone and Nitrate to Stationary Source NOx Emission Reductions in the Eastern USA.” Atmospheric Environment 45: 1084–94.

[R13] Canham CD, and Murphy L. 2016. “The Demography of Tree Species Response to Climate: Sapling and Canopy Tree Growth.” Ecosphere 7: e01474.

[R14] Canham CD, and Murphy L. 2017. “The Demography of Tree Species Response to Climate: Sapling and Canopy Tree Survival.” Ecosphere 8: e01701.

[R15] Carter TS, Clark CM, Fenn ME, Jovan S, Perakis SS, Riddell J, Schaberg PG, Greaver TL, and Hastings MG. 2017. “Mechanisms of Nitrogen Deposition Effects on Temperate Forest Lichens and Trees.” Ecosphere 8: e01717. 10.1002/ecs2.1717 PMC8318115 34327038

[R16] Chen J, Zhao R, and Li Z. 2004. “Voronoi-Based k-Order Neighbour Relations for Spatial Analysis.” ISPRS Journal of Photogrammetry and Remote Sensing 59: 60–72.

[R17] Chen T, and Guestrin C. 2016. “XGBoost: A Scalable Tree Boosting System.” In Proceedings of the ACM SIGKDD International Conference on Knowledge Discovery and Data Mining, New York, NY: Association for Computing Machinery. 785–794.

[R18] Chen Y, Shen H, Shih JS, Russell AG, Shao S, Hu Y, Odman MT, 2020. “Greater Contribution from Agricultural Sources to Future Reactive Nitrogen Deposition in the United States.” Earth’s Future 8: e2019EF001453.

[R19] Clark CM, Bell MD, Boyd JW, Compton JE, Davidson EA, Davis C, Fenn ME, Geiser L, Jones L, and Blett TF. 2017. “Nitrogen-Induced Terrestrial Eutrophication: Cascading Effects and Impacts on Ecosystem Services.” Ecosphere 8: e01877. 39669011 10.1002/ecs2.1877 PMC11636942

[R20] Clark CM, Phelan J, Ash J, Buckley J, Cajka J, Horn K, Thomas RQ, and Sabo RD. 2023. “Future Climate Change Effects on US Forest Composition May Offset Benefits of Reduced Atmospheric Deposition of N and S.” Global Change Biology 29: 4793–4810. 37417247 10.1111/gcb.16817 PMC11166206

[R21] Clark CM, Phelan J, Doraiswamy P, Buckley J, Cajka JC, Dennis RL, Lynch J, Nolte CG, and Spero TL. 2018. “Atmospheric Deposition and Exceedances of Critical Loads from 1800–2025 for the Conterminous United States.” Ecological Applications 28: 978–1022. 29714821 10.1002/eap.1703 PMC8637495

[R22] Clark CM, Simkin SM, Allen EB, Bowman WD, Belnap J, Brooks ML, Collins SL, 2019. “Potential Vulnerability of 348 Herbaceous Species to Atmospheric Deposition of Nitrogen and Sulfur in the United States.” Nature Plants 5: 697–705. 31263243 10.1038/s41477-019-0442-8 PMC10790282

[R23] Clark CM, Thomas RQ, and Horn KJ. 2023. “Aboveground Tree Carbon Storage in Response to Nitrogen Deposition in the U.S. Is Heterogeneous and May Have Weakened.” Communications Earth & Environment 4: 35–38. 10.1038/s43247-023-00677-w PMC10262689 37325084

[R24] Clark JS, Iverson L, Woodall CW, Allen CD, Bell DM, Bragg DC, D’Amato AW, 2016. “The Impacts of Increasing Drought on Forest Dynamics, Structure, and Biodiversity in the United States.” Global Change Biology 22: 2329–52. 26898361 10.1111/gcb.13160

[R25] Coughlin J. 2023. “All Species N Critical Loads.” Figshare. Dataset 10.6084/m9.figshare.22692964.v1.

[R26] Coughlin J. 2024a. “Raw Tree-Level and SHAP Metadata.” Figshare. Dataset 10.6084/m9.figshare.25976689.v1.

[R27] Coughlin J. 2024b. “Raw Tree-Level Survival Probability Data with Environmental Conditions.” Figshare. Dataset 10.6084/m9.figshare.25976215.v1.

[R28] Coughlin J. 2024c. “Raw Tree-Level Growth Data with Environmental Conditions.” Figshare. Dataset 10.6084/m9.figshare.25976182.v1.

[R29] Coughlin J. 2024d. “Raw SHAP Survival Probability Data.” Figshare. Dataset 10.6084/m9.figshare.25976155.v1.

[R30] Coughlin J. 2024e. “Raw SHAP Growth Data.” Figshare. Dataset 10.6084/m9.figshare.25976074.v1.

[R31] Coughlin JG, Clark CM, Pardo LH, Sabo RD, and Ash JD. 2023. “Sensitive Tree Species Remain at Risk despite Improved Air Quality Benefits to US Forests.” Nature Sustainability 6: 1607–19. 10.1038/s41893-023-01203-8 PMC11457011 39376716

[R32] Daly C, Halbleib M, Smith JI, Gibson WP, Doggett MK, Taylor GH, Curtis J, and Pasteris PP. 2008. “Physiographically Sensitive Mapping of Climatological Temperature and Precipitation across the Conterminous United States.” International Journal of Climatology 28: 2031–64.

[R33] De Vries W, Reinds GJ, Gundersen P, and Sterba H. 2006. “The Impact of Nitrogen Deposition on Carbon Sequestration in European Forests and Forest Soils.” Global Change Biology 12: 1151–73.

[R34] Dietze MC, and Moorcroft PR. 2011. “Tree Mortality in the Eastern and Central United States: Patterns and Drivers.” Global Change Biology 17: 3312–26.

[R35] Driscoll CT, Whitall D, Aber J, Boyer E, Castro M, Cronan C, Goodale CL, 2003. “Nitrogen Pollution in the Northeastern United States: Sources, Effects, and Management Options.” Bioscience 53: 357–374.

[R36] Du E, De Vries W, Galloway JN, Hu X, and Fang J. 2014. “Changes in Wet Nitrogen Deposition in the United States between 1985 and 2012.” Environmental Research Letters 9: 095004.

[R37] Fawcett T. 2006. “An Introduction to ROC Analysis.” Pattern Recognition Letters 27: 861–874.

[R38] Fenn ME, Bytnerowicz A, Schilling SL, Vallano DM, Zavaleta ES, Weiss SB, Morozumi C, Geiser LH, and Hanks K. 2018. “On-Road Emissions of Ammonia: An Underappreciated Source of Atmospheric Nitrogen Deposition.” Science of the Total Environment 625: 909–919. 29996462 10.1016/j.scitotenv.2017.12.313

[R39] Fenn ME, Poth MA, Aber JD, Baron JS, Bormann BT, Johnson DW, Lemly AD, McNulty SG, Ryan DF, and Stottlemyer R. 1998. “Nitrogen Excess in North American Ecosystems: Predisposing Factors, Ecosystem Responses, and Management Strategies.” Ecological Applications 8: 706–733.

[R40] Fenn ME, Preisler HK, Fried JS, Bytnerowicz A, Schilling SL, Jovan S, and Kuegler O. 2020. “Evaluating the Effects of Nitrogen and Sulfur Deposition and Ozone on Tree Growth and Mortality in California Using a Spatially Comprehensive Forest Inventory.” Forest Ecology and Management 465: 118084.

[R41] Galloway JN, Dentener FJ, Capone DG, Boyer EW, Howarth RW, Seitzinger SP, Asner GP, 2004. “Nitrogen Cycles: Past, Present, and Future.” Biogeochemistry 70: 153–226.

[R42] Galloway JN, Townsend AR, Erisman JW, Bekunda M, Cai Z, Freney JR, Martinelli LA, Seitzinger SP, and Sutton MA. 2008. “Transformation of the Nitrogen Cycle: Recent Trends, Questions, and Potential Solutions.” Science 320: 889–892. 18487183 10.1126/science.1136674

[R43] Geiser LH, Nelson PR, Jovan SE, Root HT, and Clark CM. 2019. “Assessing Ecological Risks from Atmospheric Deposition of Nitrogen and Sulfur to US Forests Using Epiphytic Macrolichens.” Diversity 11: 87. 10.3390/d11060087 PMC8549857 34712100

[R44] Geiser LH, Root H, Smith RJ, Jovan SE, St Clair L, and Dillman KL. 2021. “Lichen-Based Critical Loads for Deposition of Nitrogen and Sulfur in US Forests.” Environmental Pollution 291: 118187. 34563846 10.1016/j.envpol.2021.118187

[R45] Gilliam FS, Burns DA, Driscoll CT, Frey SD, Lovett GM, and Watmough SA. 2019. “Decreased Atmospheric Nitrogen Deposition in Eastern North America: Predicted Responses of Forest Ecosystems.” Environmental Pollution 244: 560–574. 30384062 10.1016/j.envpol.2018.09.135

[R46] Girardin MP, Bouriaud O, Hogg EH, Kurz W, Zimmermann NE, Metsaranta JM, De Jong R, 2016. “No Growth Stimulation of Canada’s Boreal Forest under Half-Century of Combined Warming and CO2 Fertilization.” Proceedings of the National Academy of Sciences of the United States of America 113: E8406–E8414. 27956624 10.1073/pnas.1610156113 PMC5206510

[R47] Gold CM, Remmele PR, and Roos T. 1997. “Voronoi Methods in GIS.” In Algorithmic Foundations of Geographic Information Systems. Berlin, Heidelberg: Springer. 21–35.

[R48] Goldstein A, Kapelner A, Bleich J, and Pitkin E. 2015. “Peeking inside the Black Box: Visualizing Statistical Learning with Plots of Individual Conditional Expectation.” Journal of Computational and Graphical Statistics 24: 44–65.

[R49] Gonzalez P, Battles JJ, Collins BM, Robards T, and Saah DS. 2015. “Aboveground Live Carbon Stock Changes of California Wildland Ecosystems, 2001–2010.” Forest Ecology and Management 348: 68–77.

[R50] Grantz DA, Gunn S, and Vu HB. 2006. “O3 Impacts on Plant Development: A Meta-Analysis of Root/Shoot Allocation and Growth.” Plant, Cell and Environment 29: 1193–1209. 10.1111/j.1365-3040.2006.01521.x 17080943

[R51] Högberg P, Fan H, Quist M, Binkley D, and Tamm CO. 2006. “Tree Growth and Soil Acidification in Response to 30 Years of Experimental Nitrogen Loading on Boreal Forest.” Global Change Biology 12: 489–499.

[R52] Horn KJ, Quinn Thomas R, Clark CM, Pardo LH, Fenn ME, Lawrence GB, Perakis SS, 2018. “Growth and Survival Relationships of 71 Tree Species with Nitrogen and Sulfur Deposition across the Conterminous U.S.” PLoS One 13: e0205296. 30335770 10.1371/journal.pone.0205296 PMC6193662

[R53] Hynicka JD, Pett-Ridge JC, and Perakis SS. 2016. “Nitrogen Enrichment Regulates Calcium Sources in Forests.” Global Change Biology 22: 4067–79. 27135298 10.1111/gcb.13335

[R54] Idso SB, and Kimball BA. 1993. “Tree Growth in Carbon Dioxide Enriched Air and Its Implications for Global Carbon Cycling and Maximum Levels of Atmospheric CO2.” Global Biogeochemical Cycles 7: 537–555.

[R55] Jenkins JC, Chojnacky DC, Heath LS, and Birdsey RA. 2003. “National-Scale Biomass Estimators for United States Tree Species.” Forest Science 49: 12–35.

[R56] Jenny H. 1994. Factors of Soil Formation: A System of Quantitative Pedology. New York: Page Courier Dover Publications.

[R57] Jo I, Fei S, Oswalt CM, Domke GM, and Phillips RP. 2019. “Shifts in Dominant Tree Mycorrhizal Associations in Response to Anthropogenic Impacts.” Science Advances 5(4): eaav6358. 10.1126/sciadv.aav6358. 30989116 PMC6457943

[R58] Kallarackal J, and Roby TJ. 2012. “Responses of Trees to Elevated Carbon Dioxide and Climate Change.” Biodiversity and Conservation 21: 1327–42.

[R59] Karlsson PE, Braun S, Broadmeadow M, Elvira S, Emberson L, Gimeno BS, Le Thiec D, 2007. “Risk Assessments for Forest Trees: The Performance of the Ozone Flux Versus the AOT Concepts.” Environmental Pollution 146: 608–616. 16938368 10.1016/j.envpol.2006.06.012

[R60] Karnosky DF, Percy KE, Mankovska B, Prichard T, Noormets A, Dickson RE, Jepsen E, and Isebrands JG. 2003. “Ozone Affects the Fitness of Trembling Aspen.” Developments in Environmental Science 3: 199–209.

[R61] Karnosky DF, Skelly JM, Percy KE, and Chappelka AH. 2007. “Perspectives Regarding 50 Years of Research on Effects of Tropospheric Ozone Air Pollution on US Forests.” Environmental Pollution 147(3): 489–506. 17084004 10.1016/j.envpol.2006.08.043

[R62] Klos RJ, Wang GG, Bauerle WL, and Rieck JR. 2009. “Drought Impact on Forest Growth and Mortality in the Southeast USA: An Analysis Using Forest Health and Monitoring Data.” Ecological Applications 19: 699–708. 19425432 10.1890/08-0330.1

[R63] Kohut RJ, Laurence JA, Amundson RG, Rabaand RM, and Melkonian JJ. 1990. “Effects of Ozone and Acidic Precipitation on the Growth and Photosynthesis of Red Spruce after Two Years of Exposure.” Water, Air, and Soil Pollution 51: 277–286.

[R64] Laurence JA, Kohut RJ, and Amundson RG. 1989. “Response of Red Spruce Seedlings Exposed to Ozone and Simulated Acidic Precipitation in the Field.” Archives of Environmental Contamination and Toxicology 18: 285–290.

[R65] LeBauer DS, and Treseder KK. 2008. “Nitrogen Limitation of Net Primary Productivity in Terrestrial Ecosystems Is Globally Distributed.” Ecology 89: 371–79. 18409427 10.1890/06-2057.1

[R66] Lee EH, Andersen CP, Beedlow PA, Tingey DT, Koike S, Dubois JJ, Kaylor SD, 2022. “Ozone Exposure-Response Relationships Parametrized for Sixteen Tree Species with Varying Sensitivity in the United States.” Atmospheric Environment 284: 1–16. 35775067 10.1016/j.atmosenv.2022.119191 PMC9237886

[R67] Lefohn AS, and Runeckles VC. 1987. “Establishing Standards to Protect Vegetation-Ozone Exposure/Dose Considerations.” Atmospheric Environment (1967) 21: 561–68.

[R68] Li Y., Schichtel BA, Walker JT, Schwede DB, Chen X, Lehmann CMB, Puchalski MA, Gay DA, and Collett JL. 2016. “Increasing Importance of Deposition of Reduced Nitrogen in the United States.” Proceedings of the National Academy of Sciences of the United States of America 113: 5874–79. 27162336 10.1073/pnas.1525736113 PMC4889379

[R69] Liese R, Lübbe T, Albers NW, and Meier IC. 2018. “The Mycorrhizal Type Governs Root Exudation and Nitrogen Uptake of Temperate Tree Species.” Tree Physiology 38: 83–95. 29126247 10.1093/treephys/tpx131

[R70] Likens GE, Driscoll CT, and Buso DC. 1996. “Long-Term Effects of Acid Rain: Response and Recovery of a Forest Ecosystem.” Science 272: 244–46.

[R71] Liu L, and Greaver TL. 2010. “A Global Perspective on Belowground Carbon Dynamics under Nitrogen Enrichment.” Ecology Letters 13: 819–828. 20482580 10.1111/j.1461-0248.2010.01482.x

[R72] Lundberg SM, and Lee SI. 2017. “A Unified Approach to Interpreting Model Predictions.” In Advances in Neural Information Processing Systems 30. Red Hook, NY: Curran Associates Inc.

[R73] Matson P, Lohse KA, and Hall SJ. 2002. “The Globalization of Nitrogen Deposition: Consequences for Terrestrial Ecosystems.” Ambio 31: 113–19. 10.1579/0044-7447-31.2.113 12077999

[R74] NADP CLAD Committee. 2017. “CLAD Critical Load Definitions, Version 1.1.”

[R75] NADP Program Office, W. S. L. of H. 465 H. M. M. W. 53706. 2022. “National Atmospheric Deposition Program (NRSP-3).”

[R76] Nilsson J. 1988. “Critical Loads for Sulphur and Nitrogen.” In Air Pollution and Ecosystems, edited by Mathy P, 85–91. Dordrecht: Springer.

[R77] Novak K, Schaub M, Fuhrer J, Skelly JM, Hug C, Landolt W, Bleuler P, and Kräuchi N. 2005. “Seasonal Trends in Reduced Leaf Gas Exchange and Ozone-Induced Foliar Injury in Three Ozone Sensitive Woody Plant Species.” Environmental Pollution 136: 33–45. 15809106 10.1016/j.envpol.2004.12.018

[R78] Pardo LH, Fenn ME, Goodale CL, Geiser LH, Driscoll CT, Allen EB, Baron JS, 2011. “Effects of Nitrogen Deposition and Empirical Nitrogen Critical Loads for Ecoregions of the United States.” Ecological Applications 21: 3049–82.

[R79] Pavlovic NR, Chang SY, Huang J, Craig K, Clark C, Horn K, and Driscoll CT. 2023. “Empirical Nitrogen and Sulfur Critical Loads of U.S. Tree Species and their Uncertainties with Machine Learning.” Science of the Total Environment 857: 159252. 36216054 10.1016/j.scitotenv.2022.159252 PMC10241478

[R80] PRISM Climate Group, O. S. U. 2021. “PRISM Data.”

[R81] Robin-Abbott MJ, and Pardo LH. 2017. “How Climatic Conditions, Site, and Soil Characteristics Affect Tree Growth and Critical Loads of Nitrogen for Northeastern Tree Species.” General Technical Report NRS-172. Newtown Square, PA: U.S. Department of Agriculture, Forest Service, Northern Research Station. 143 p.

[R82] Rogers HH, Bingham GE, Cure JD, Smith JM, and Surano KA. 1983. “Responses of Selected Plant Species to Elevated Carbon Dioxide in the Field.” Journal of Environmental Quality 12: 569–574.

[R83] Sabo RD, Clark CM, Bash J, Sobota D, Cooter E, Dobrowolski JP, Houlton BZ, 2019. “Decadal Shift in Nitrogen Inputs and Fluxes across the Contiguous United States: 2002–2012.” Journal of Geophysical Research: Biogeosciences 124: 3104–24. 10.1029/2019JG005110 PMC12180915 40547514

[R84] Saylor R, Myles LT, Sibble D, Caldwell J, and Xing J. 2015. “Recent Trends in Gas-Phase Ammonia and PM2.5 Ammonium in the Southeast United States.” Journal of the Air and Waste Management Association 65: 347–357. 25947130 10.1080/10962247.2014.992554

[R85] Schwede DB, and Lear GG. 2014. “A Novel Hybrid Approach for Estimating Total Deposition in the United States.” Atmospheric Environment 92: 207–220.

[R86] Shapley LS 1953. “A Value for n-Person Games.” In Contributions to the Theory of Games II, edited by Kuhn H. and Tucker A, 307–317. Princeton, NJ: Princeton University Press.

[R87] Simkin SM, Allen EB, Bowman WD, Clark CM, Belnap J, Brooksf ML, Cade BS, 2016. “Conditional Vulnerability of Plant Diversity to Atmospheric Nitrogen Deposition across the United States.” Proceedings of the National Academy of Sciences of the United States of America 113: 4086–91. 27035943 10.1073/pnas.1515241113 PMC4839424

[R88] Soong JL, Janssens IA, Grau O, Margalef O, Stahl C, Van Langenhove L, Urbina I, 2020. “Soil Properties Explain Tree Growth and Mortality, but Not Biomass, across Phosphorus-Depleted Tropical Forests.” Scientific Reports 10: 2302. 32041976 10.1038/s41598-020-58913-8 PMC7010742

[R89] Stevens CJ, Dupr C, Dorland E, Gaudnik C, Gowing DJG, Bleeker A, Diekmann M, 2010. “Nitrogen Deposition Threatens Species Richness of Grasslands across Europe.” Environmental Pollution 158: 2940–45. 20598409 10.1016/j.envpol.2010.06.006

[R90] Štrumbelj E, and Kononenko I. 2014. “Explaining Prediction Models and Individual Predictions with Feature Contributions.” Knowledge and Information Systems 41: 647–665.

[R91] Sullivan TJ, Lawrence GB, Bailey SW, McDonnell TC, Beier CM, Weathers KC, McPherson GT, and Bishop DA. 2013. “Effects of Acidic Deposition and Soil Acidification on Sugar Maple Trees in the Adirondack Mountains, New York.” Environmental Science and Technology 47: 12687–94. 24102084 10.1021/es401864w

[R92] Thomas RQ, Canham CD, Weathers KC, and Goodale CL. 2010. “Increased Tree Carbon Storage in Response to Nitrogen Deposition in the US.” Nature Geoscience 3: 13–17.

[R93] U.S. Environmental Protection Agency. 2007. “Review of the National Ambient Air Quality Standards for Ozone: Policy Assessment of Scientific and Technical Information.” OAQPS Staff Paper.

[R94] U.S. Environmental Protection Agency. 2022. “Ozone W126 Index.”

[R95] United States Department of Agriculture, N. R. C. S. 2020. “Soil Survey Staff. Gridded National Soil Survey Geographic (gNATSGO) Database for the Conterminous United States.” https://www.nrcs.usda.gov/resources/data-and-reports/gridded-national-soil-survey-geographic-database-gnatsgo.

[R96] Wallace ZP, Lovett GM, Hart JE, and Machona B. 2007. “Effects of Nitrogen Saturation on Tree Growth and Death in a Mixed-Oak Forest.” Forest Ecology and Management 243: 210–18.

[R97] Westfall JA, Coulston JW, Gray AN, Shaw JD, Radtke PJ, Walker DM, Weiskittel AR, 2024. “A National-Scale Tree Volume, Biomass, and Carbon Modeling System for the United States.” General Technical Report WO-104. Washington, DC: U.S. Department of Agriculture, Forest Service. 60 p. 10.2737/WO-GTR-104.

[R98] Wilkins K, Clark C, and Aherne J. 2022. “Ecological Thresholds under Atmospheric Nitrogen Deposition for 1200 Herbaceous Species and 24 Communities across the United States.” Global Change Biology 28: 2381–95. 34986509 10.1111/gcb.16076 PMC9770646

[R99] Woodall CW, Domke GM, Riley KL, Oswalt CM, Crocker SJ, and Yohe GW. 2013. “A Framework for Assessing Global Change Risks to Forest Carbon Stocks in the United States.” PLoS One 8: e73222. 24039889 10.1371/journal.pone.0073222 PMC3769302

[R100] Woudenberg SW, Conkling BL, O’connell BM, Lapoint EB, Turner JA, and Waddell KL. 2010. “The Forest Inventory and Analysis Database: Database Description and Users Manual Version 4.0 for Phase 2.” General Technical Report RMRS-GTR-245. Fort Collins, CO: U.S. Department of Agriculture, Forest Service, Rocky Mountain Research Station. 336 p.

[R101] Zhang Y, Mathur R, Bash JO, Hogrefe C, Xing J, and Roselle SJ. 2018. “Long-Term Trends in Total Inorganic Nitrogen and Sulfur Deposition in the US from 1990 to 2010.” Atmospheric Chemistry and Physics 18: 9091–9106. 30079084 10.5194/acp-18-9091-2018 PMC6069975

