# Supplementary material for: Characterizing localized nitrogen sensitivity of tree species and the associated influences of mediating factors
Source: Ecosphere. Author manuscript; Available in PMC 2025 Jul 3. (PMC11694898; doi:10.1002/ecs2.4925)
Supplement: Supplement1 [file NIHMS2017876-supplement-Supplement1.pdf]

ECOSPHERE

**APPENDIX S1**

# Characterizing localized nitrogen sensitivity of tree species and the associated influences of mediating factors

Justin G. Coughlin[1], Shih Ying Chang[1], Kenneth Craig[1], Charles Scarborough[1], Charles T. Driscoll[2], Christopher M. Clark[3], Nathan R. Pavlovic[1]*

[1] Sonoma Technology, Inc., 1450 N. McDowell Blvd., Suite 200, Petaluma, CA 94954 USA

[2] Department of Civil and Environmental Engineering, Syracuse University, 151 Link Hall Syracuse, NY 13244 USA

[3]U.S. Environmental Protection Agency, Office of Research and Development, 1200 Pennsylvania Avenue, NW, Mail Code: 8101R, Washington, DC 20460 USA

* Correspondence: npavlovic@sonomatech.com

Summary: This supporting information consists of 6 tables, 51 additional figures, and additional results for the ten selected species. Additional results for all evaluated species, without bootstrap modeling, can be found at:

https://figshare.com/articles/dataset/All_Species_N_Critical_Loads/22692964 (Table S7).

**Contents**

Model Performance Results....................2

Additional Results....................7

Additional Figures....................19

References....................60

## Model Performance Results

Table S1. Growth ML model performance of this study compared to results from Pavlovic et al. (2023) where $R^2$ is the coefficient of variation, RMSE is the root mean square error, and MAE is the mean absolute error.

| ***SPCD*** | **Common Name** | **Scientific Name** | **This Study** | | | **Pavlovic et al. (2023)** | | |
|---|---|---|---|---|---|---|---|---|
| | | | $R^2$ | RMSE | MAE | $R^2$ | RMSE | MAE |
| *97* | red spruce | *Picea rubens* | 0.64 | 0.73 | 0.53 | 0.57 | 0.85 | 0.64 |
| *122* | ponderosa pine | *Pinus ponderosa* | 0.61 | 1.62 | 1.12 | 0.61 | 1.56 | 1.07 |
| *202* | Douglas-fir | *Pseudotsuga menziesii* | 0.72 | 4.24 | 2.64 | 0.71 | 4.56 | 2.84 |
| *263* | western hemlock | *Tsuga heterophylla* | 0.69 | 2.37 | 1.54 | 0.67 | 2.45 | 1.63 |
| *318* | sugar maple | *Acer saccharum* | 0.63 | 1.51 | 1.05 | 0.58 | 1.63 | 1.17 |
| *375* | paper birch | *Betula papyrifera* | 0.46 | 0.75 | 0.57 | 0.38 | 0.79 | 0.63 |
| *621* | yellow-poplar | *Liriodendron tulipifera* | 0.69 | 2.61 | 1.77 | 0.64 | 2.92 | 2.08 |
| *742* | eastern cottonwood | *Populus deltoides* | 0.73 | 5.40 | 3.63 | 0.67 | 5.76 | 3.97 |
| *746* | quaking aspen | *Populus tremuloides* | 0.71 | 0.85 | 0.59 | 0.62 | 1.00 | 0.71 |
| *762* | black cherry | *Prunus serotina* | 0.59 | 1.50 | 1.05 | 0.52 | 1.68 | 1.21 |

Table S2. A table of the survival probability ML model performance of this study compared to results from Pavlovic et al. (2023) where AUC is the area under the curve.

| ***SPCD*** | **Common Name** | **Scientific Name** | **This Study** | | | **Pavlovic et al. (2023)** | | |
|---|---|---|---|---|---|---|---|---|
| | | | AUC | False Positive | False Negative | AUC | False Positive | False Negative |
| *97* | red spruce | *Picea rubens* | 0.819 | 0.03 | 0.58 | 0.779 | 0.05 | 0.68 |
| *122* | ponderosa pine | *Pinus ponderosa* | 0.843 | 0.04 | 0.49 | 0.827 | 0.03 | 0.49 |
| *202* | Douglas-fir | *Pseudotsuga menziesii* | 0.850 | 0.05 | 0.47 | 0.801 | 0.04 | 0.58 |
| *263* | western hemlock | *Tsuga heterophylla* | 0.712 | 0.04 | 0.77 | 0.659 | 0.05 | 0.82 |
| *318* | sugar maple | *Acer saccharum* | 0.762 | 0.02 | 0.72 | 0.720 | 0 | 0.99 |
| *375* | paper birch | *Betula papyrifera* | 0.817 | 0.12 | 0.4 | 0.775 | 0.1 | 0.54 |
| *621* | yellow-poplar | *Liriodendron tulipifera* | 0.839 | 0.05 | 0.55 | 0.734 | 0.03 | 0.74 |
| *742* | eastern cottonwood | *Populus deltoides* | 0.780 | 0.09 | 0.56 | 0.733 | 0.13 | 0.62 |
| *746* | quaking aspen | *Populus tremuloides* | 0.778 | 0.14 | 0.45 | 0.742 | 0.03 | 0.78 |
| *762* | black cherry | *Prunus serotina* | 0.756 | 0.08 | 0.64 | 0.710 | 0.06 | 0.76 |

Table S3. Species-wide N CLs from this study (mean, standard deviation [SD] of local median N CLs), lower and upper bounds from both Horn et al. (2018) and Pavlovic et al. (2023). The maximum likelihood estimate is included for Horn et al. (2018) and the median N CL is included for Pavlovic et al. (2023).

| SPCD | Scientific Name | Common Name | Mean N CL (kg N ha$^{-1}$ yr$^{-1}$) | SD N CL (kg N ha$^{-1}$ yr$^{-1}$) | Lower Bound Horn et al. (2018) N CL (kg N ha$^{-1}$ yr$^{-1}$) | Upper Bound Horn et al. (2018) N CL (kg N ha$^{-1}$ yr$^{-1}$) | Horn et al. (2018) N CL (kg N ha$^{-1}$ yr$^{-1}$) | Lower Bound Pavlovic et al. (2023) N CL (kg N ha$^{-1}$ yr$^{-1}$) | Upper Bound Pavlovic et al. (2023) N CL (kg N ha$^{-1}$ yr$^{-1}$) | Median Pavlovic et al. (2023) N CL (kg N ha$^{-1}$ yr$^{-1}$) |
|---|---|---|---|---|---|---|---|---|---|---|
| | | | | | **Growth** | | | | | |
| 97 | *Picea rubens* | red spruce | 6.34 | 1.92 | 0.01 | 0.01 | 0.01 | 3.83 | 10.52 | 4.30 |
| 122 | *Pinus ponderosa* | ponderosa pine | 4.89 | 2.08 | | | | 3.01 | 3.55 | 3.11 |
| 202 | *Pseudotsuga menziesii* | Douglas-fir | 4.70 | 1.68 | 21.04 | 33.85 | 33.85 | 5.04 | 7.86 | 6.92 |
| 263 | *Tsuga heterophylla* | western hemlock | 4.24 | 1.14 | 6.01 | 6.01 | 6.01 | 4.33 | 4.68 | 4.38 |
| 318 | *Acer saccharum* | sugar maple | 8.02 | 2.55 | | | | 3.53 | 9.32 | 9.11 |
| 375 | *Betula papyrifera* | paper birch | 7.56 | 1.93 | | | | 3.74 | 11.57 | 3.78 |
| 621 | *Liriodendron tulipifera* | yellow-poplar | 14.50 | 4.01 | 62.20 | 62.20 | 62.20 | 12.93 | 24.64 | 21.55 |
| 742 | *Populus deltoides* | eastern cottonwood | 11.16 | 1.97 | 58.84 | 58.84 | 58.84 | 11.46 | 14.55 | 13.25 |
| 746 | *Populus tremuloides* | quaking aspen | 7.85 | 1.42 | 11.15 | 11.15 | 11.15 | 2.69 | 10.50 | 3.31 |
| 762 | *Prunus serotina* | black cherry | 10.27 | 1.73 | 13.44 | 15.43 | 14.58 | 9.36 | 16.01 | 10.59 |
| | | | | | **Survival** | | | | | |
| 97 | *Picea rubens* | red spruce | 9.21 | 1.50 | | | | 6.47 | 11.21 | 7.50 |
| 122 | *Pinus ponderosa* | ponderosa pine | 4.27 | 1.93 | | | | 1.98 | 8.04 | 6.94 |
| 202 | *Pseudotsuga menziesii* | Douglas-fir | 3.76 | 1.82 | 1.95 | 1.95 | 1.95 | 2.40 | 4.04 | 2.95 |
| 263 | *Tsuga heterophylla* | western hemlock | 3.09 | 1.16 | 2.11 | 2.11 | | 2.24 | 2.63 | 2.31 |
| 318 | *Acer saccharum* | sugar maple | 9.81 | 1.88 | | | | 8.61 | 13.45 | 10.62 |
| 375 | *Betula papyrifera* | paper birch | 7.73 | 1.47 | 9.37 | 9.37 | 9.37 | 7.84 | 13.39 | 13.29 |
| 621 | *Liriodendron tulipifera* | yellow-poplar | 11.69 | 2.22 | | | | 6.01 | 8.49 | 7.16 |
| 742 | *Populus deltoides* | eastern cottonwood | 11.12 | 0.56 | | | | 6.74 | 14.58 | 9.81 |
| 746 | *Populus tremuloides* | quaking aspen | 8.20 | 1.46 | 4.21 | 4.21 | 4.21 | 1.71 | 12.12 | 2.62 |
| 762 | *Prunus serotina* | black cherry | 10.25 | 1.28 | 8.71 | 15.86 | 8.71 | 7.35 | 13.55 | 8.11 |

Table S4. Nitrogen deposition correlations, by species, for all other growth predictor variables across all FIA plots where the species is present.

| | | | N Deposition (kg N ha-1 yr-1) Correlation | | | | | | | | | |
|---|---|---|---|---|---|---|---|---|---|---|---|---|
| SPCD | Scientific Name | Common Name | Initial plot basal area (m^2/ha) | Above ground biomass (kg-Carbon) | Average yearly precipitation for elapsed timed (dm) | Average yearly temperature for elapsed time (Degrees K) | S Deposition (kg S ha-1 yr-1) | pH | Organic Matter (%) | Percent Clay (%) | Mean PDSI | Mean O3 (W126) |
| 97 | *Picea rubens* | red spruce | 0.31 | 0.09 | 0.49 | 0.38 | 0.84 | -0.11 | -0.04 | -0.17 | 0.04 | 0.77 |
| 122 | *Pinus ponderosa* | ponderosa pine | 0.10 | -0.18 | 0.18 | 0.36 | 0.90 | 0.02 | -0.01 | 0.18 | -0.01 | 0.67 |
| 202 | *Pseudotsuga menziesii* | Douglas-fir | 0.12 | 0.02 | 0.34 | 0.19 | 0.65 | -0.11 | 0.07 | 0.09 | 0.16 | 0.41 |
| 263 | *Tsuga heterophylla* | western hemlock | -0.01 | 0.03 | 0.33 | -0.03 | 0.34 | 0.00 | 0.16 | -0.09 | 0.17 | 0.00 |
| 318 | *Acer saccharum* | sugar maple | -0.04 | 0.01 | 0.25 | 0.67 | 0.67 | 0.28 | -0.25 | 0.51 | -0.02 | 0.74 |
| 375 | *Betula papyrifera* | paper birch | -0.07 | 0.04 | -0.18 | 0.49 | 0.42 | 0.32 | -0.13 | 0.03 | -0.14 | 0.62 |
| 621 | *Liriodendron tulipifera* | yellow-poplar | -0.01 | 0.05 | -0.02 | -0.22 | 0.41 | 0.11 | -0.10 | 0.04 | 0.21 | 0.34 |
| 742 | *Populus deltoides* | eastern cottonwood | 0.10 | 0.08 | 0.28 | -0.03 | 0.35 | -0.15 | 0.20 | 0.05 | -0.07 | 0.14 |
| 746 | *Populus tremuloides* | quaking aspen | -0.26 | -0.02 | 0.31 | 0.59 | 0.60 | -0.17 | 0.00 | -0.24 | 0.07 | -0.15 |
| 762 | *Prunus serotina* | black cherry | -0.02 | 0.03 | -0.10 | 0.07 | 0.33 | 0.31 | -0.16 | 0.24 | -0.06 | 0.41 |

Table S5. Nitrogen deposition correlations, by species, for all other survival predictor variables across all FIA plots where the species is present.

| | | | N Deposition (kg N ha-1 yr-1) Correlation | | | | | | | | | | | | | |
|---|---|---|---|---|---|---|---|---|---|---|---|---|---|---|---|---|
| SPCD | Scientific Name | Common Name | Height of the tallest tree in the subplot (m) | Initial subplot basal area (m^2/ha) | Ratio of tree of interest basal area to plot basal area | Initial ratio of tree of interest height to tallest tree in the subplot | Initial aboveground biomass (kg-Carbon) | Average yearly precipitation for elapsed timed (dm) | Average yearly temperature for elapsed time (Degrees K) | S Deposition (kg S ha-1 yr-1) | Elapsed time (years) | pH | Organic Matter (%) | Percent Clay (%) | Mean PDSI | Mean O3 (W126) |
| 97 | *Picea rubens* | red spruce | 0.20 | 0.23 | 0.09 | -0.07 | 0.16 | 0.50 | 0.42 | 0.84 | -0.19 | -0.10 | -0.04 | -0.14 | 0.06 | 0.77 |
| 122 | *Pinus ponderosa* | ponderosa pine | -0.22 | 0.10 | 0.11 | 0.09 | -0.13 | 0.19 | 0.37 | 0.88 | -0.31 | 0.02 | -0.02 | 0.17 | 0.00 | 0.68 |
| 202 | *Pseudotsuga menziesii* | Douglas-fir | 0.01 | 0.09 | 0.04 | 0.03 | 0.03 | 0.33 | 0.21 | 0.65 | -0.15 | -0.11 | 0.07 | 0.09 | 0.16 | 0.40 |
| 263 | *Tsuga heterophylla* | western hemlock | -0.04 | -0.01 | -0.04 | 0.03 | 0.04 | 0.32 | -0.02 | 0.32 | -0.04 | 0.00 | 0.18 | -0.09 | 0.17 | 0.01 |
| 318 | *Acer saccharum* | sugar maple | 0.28 | 0.02 | 0.02 | -0.19 | 0.03 | 0.25 | 0.67 | 0.67 | 0.01 | 0.28 | -0.25 | 0.51 | -0.05 | 0.74 |
| 375 | *Betula papyrifera* | paper birch | 0.08 | -0.01 | -0.05 | 0.02 | 0.04 | -0.16 | 0.48 | 0.42 | -0.09 | 0.30 | -0.13 | 0.04 | -0.13 | 0.60 |
| 621 | *Liriodendron tulipifera* | yellow-poplar | 0.00 | 0.04 | -0.02 | 0.01 | 0.05 | -0.03 | -0.22 | 0.40 | 0.07 | 0.10 | -0.10 | 0.03 | 0.20 | 0.33 |
| 742 | *Populus deltoides* | eastern cottonwood | 0.28 | 0.16 | 0.05 | 0.03 | 0.05 | 0.25 | -0.06 | 0.34 | 0.01 | -0.13 | 0.20 | 0.04 | -0.06 | 0.14 |
| 746 | *Populus tremuloides* | quaking aspen | 0.05 | -0.24 | -0.18 | 0.10 | -0.03 | 0.33 | 0.59 | 0.61 | -0.20 | -0.19 | 0.00 | -0.25 | 0.09 | -0.19 |
| 762 | *Prunus serotina* | black cherry | 0.08 | 0.04 | 0.02 | -0.02 | 0.03 | -0.11 | 0.07 | 0.33 | 0.08 | 0.31 | -0.16 | 0.23 | -0.07 | 0.43 |

Table S6. Minimum and maximum N deposition experienced, by species, for both growth and survival assessments across all FIA plots where the species is present. The N deposition variance inflation factor (VIF) is also shown for each species by endpoint.

| SPCD | Scientific Name | Common Name | Max N Deposition (kg N ha-1 yr-1) | Min N Deposition (kg N ha-1 yr-1) | N Deposition VIF |
|---|---|---|---|---|---|
| | | | **Growth** | | |
| 97 | *Picea rubens* | red spruce | 12.3 | 2.6 | 6.0 |
| 122 | *Pinus ponderosa* | ponderosa pine | 13.1 | 1.0 | 8.9 |
| 202 | *Pseudotsuga menziesii* | Douglas-fir | 17.5 | 1.2 | 2.9 |
| 263 | *Tsuga heterophylla* | western hemlock | 15.0 | 1.4 | 1.5 |
| 318 | *Acer saccharum* | sugar maple | 24.7 | 3.3 | 2.8 |
| 375 | *Betula papyrifera* | paper birch | 16.8 | 2.1 | 1.9 |
| 621 | *Liriodendron tulipifera* | yellow-poplar | 33.7 | 5.2 | 1.4 |
| 742 | *Populus deltoides* | eastern cottonwood | 31.3 | 3.7 | 1.3 |
| 746 | *Populus tremuloides* | quaking aspen | 18.2 | 1.6 | 2.1 |
| 762 | *Prunus serotina* | black cherry | 29.2 | 3.7 | 1.5 |
| | | | **Survival** | | |
| 97 | *Picea rubens* | red spruce | 12.3 | 2.6 | 6.1 |
| 122 | *Pinus ponderosa* | ponderosa pine | 15.6 | 1.0 | 8.3 |
| 202 | *Pseudotsuga menziesii* | Douglas-fir | 17.5 | 1.2 | 2.8 |
| 263 | *Tsuga heterophylla* | western hemlock | 15.0 | 1.4 | 1.4 |
| 318 | *Acer saccharum* | sugar maple | 24.7 | 3.3 | 2.8 |
| 375 | *Betula papyrifera* | paper birch | 15.3 | 2.1 | 1.9 |
| 621 | *Liriodendron tulipifera* | yellow-poplar | 33.7 | 5.2 | 1.4 |
| 742 | *Populus deltoides* | eastern cottonwood | 31.3 | 3.7 | 1.3 |
| 746 | *Populus tremuloides* | quaking aspen | 18.2 | 1.6 | 2.2 |
| 762 | *Prunus serotina* | black cherry | 29.2 | 3.6 | 1.5 |

## Additional Results

*Comparison with previous work*

Here, we expand upon absolute differences between previous critical load (CL) work and this study. For growth, the largest differences were observed for yellow-poplar (-47.7 kg N $ha^{-1}$ $yr^{-1}$), eastern cottonwood (-47.7 kg N $ha^{-1}$ $yr^{-1}$), and Douglas-fir (-29.1 kg N $ha^{-1}$ $yr^{-1}$); these three were all comparisons against results from Horn et al. (2018) where N CLs were substantially higher than values found here (Figure 1 in the main manuscript). For these three species, N and S deposition correlations were all < 0.7 and variance inflation factors (VIFs) for N deposition were all below 3. Additionally, yellow-poplar and Douglas-fir did not have competing models (i.e., dAIC between models was > 2) for growth so the discrepancy is unexplainable other than differences in methodology. Using our methodology, we find that a handful of species had higher average N CLs for growth – these included ponderosa pine (1.8 kg N $ha^{-1}$ $yr^{-1}$), red spruce (2.0 kg N $ha^{-1}$ $yr^{-1}$), paper birch (3.8 kg N $ha^{-1}$ $yr^{-1}$), quaking aspen (4.5 kg N $ha^{-1}$ $yr^{-1}$), and red spruce (6.3 kg N $ha^{-1}$ $yr^{-1}$). All these species were higher than results found in Pavlovic et al. (2023), except for red spruce which was higher than the low N CL (0.01 kg N $ha^{-1}$ $yr^{-1}$) found in Horn et al. (2018) (Figure 1 in the main manuscript).

Differences in survival CLs were much more minimal than the growth endpoints (Figure 5). Absolute differences range from -5.6 to 5.6 kg N $ha^{-1}$ $yr^{-1}$ between both studies (Horn et al. 2018, Pavlovic et al. 2023) (Figure 1 in the main manuscript). Most species were found to have slightly higher N CLs for survival than what was previously found except for paper birch (-5.6 kg N $ha^{-1}$ $yr^{-1}$), ponderosa pine (-2.6 to -1.6 kg N $ha^{-1}$ $yr^{-1}$), and sugar maple (-0.8 kg N $ha^{-1}$ $yr^{-1}$). Note that

not all species (of the 140) are able to be compared against Horn et al. (2018) due to the sample size cutoff that was used within that study ($n \geq 2,000$).

*Sensitivity Analysis*

We conducted an evaluation of changing the reduction threshold at which a CL can be established to understand how variable CLs are when a higher amount of reduction than 1% (e.g., 5%) is acceptable. For this analysis, we evaluated 1%, 5%, 10%, and 20% reductions for both growth and survival probability. Generally, while comparisons between bootstrap median N CLs across different reduction thresholds were variable, the variability was minimal. This result has important context for the 95% confidence interval (CI) variability that was observed where some species had large 95% Cis for N CLs. Even though 95% CI variability was large, the resulting N CL differences amongst the 1%, 5%, 10%, and 20% reduction thresholds demonstrates that the start of the dose-response curve decline tends to lead to large reductions shortly after the initial 1% decrease.

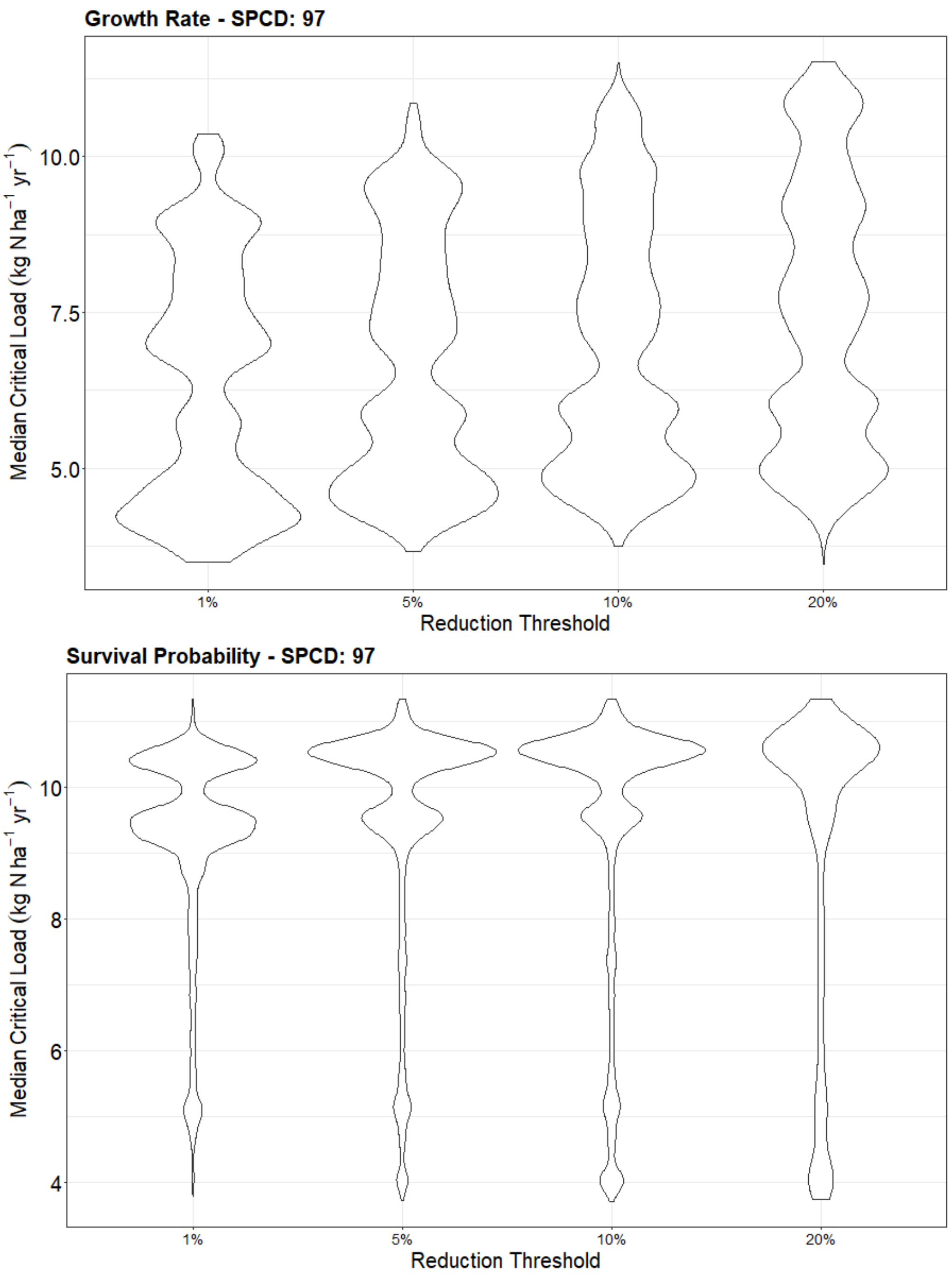


**Figure S1.** The bootstrap median N CL for red spruce for (a) growth and (b) survival probability at different reduction thresholds (1%, 5%, 10%, and 20%). Violin plots reflect the distribution of points where wider areas have a higher density of points in that CL bin.

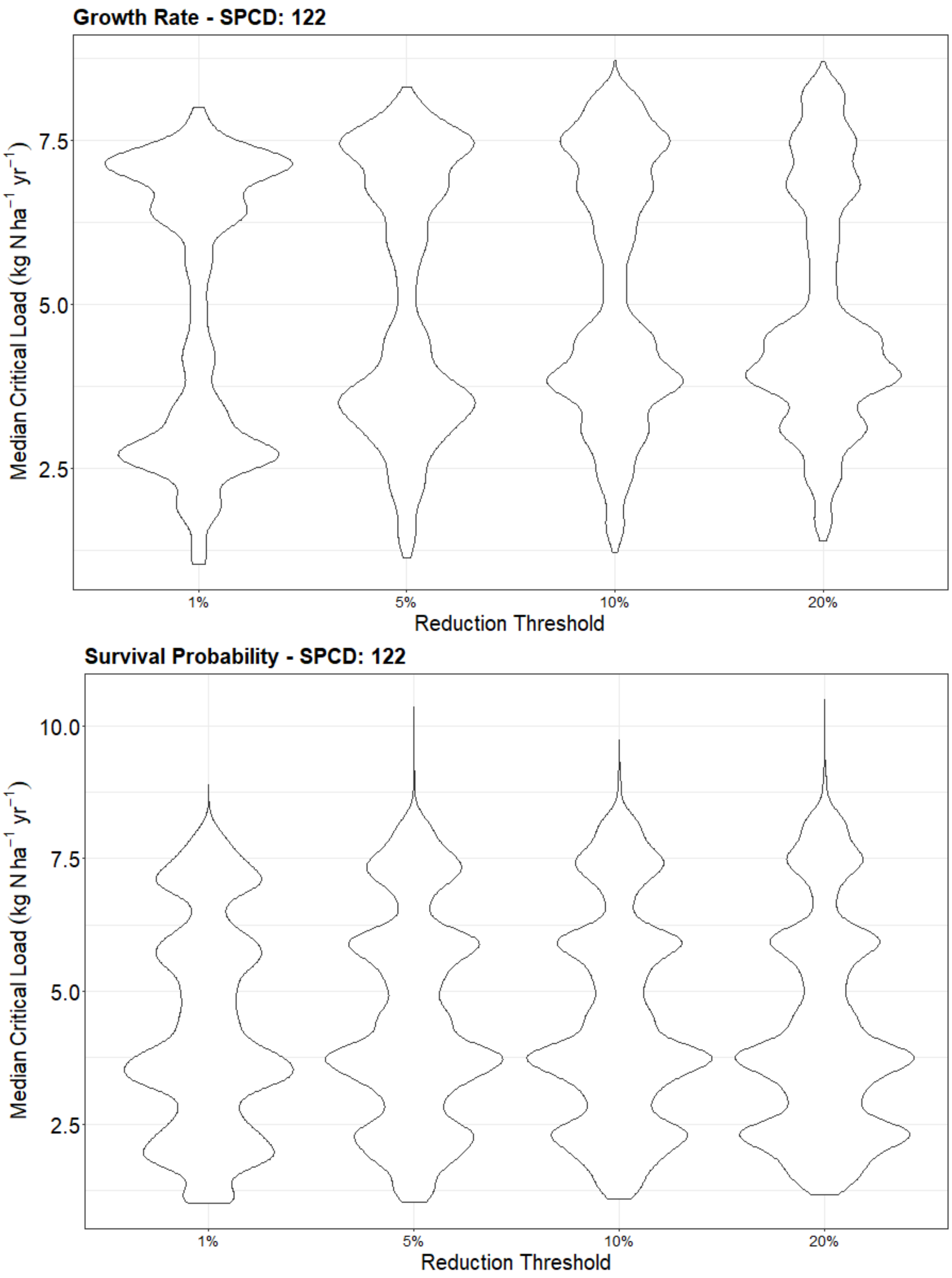


**Figure S2.** The bootstrap median N CL for ponderosa pine for (a) growth and (b) survival probability at different reduction thresholds (1%, 5%, 10%, and 20%). Violin plots reflect the distribution of points where wider areas have a higher density of points in that CL bin.

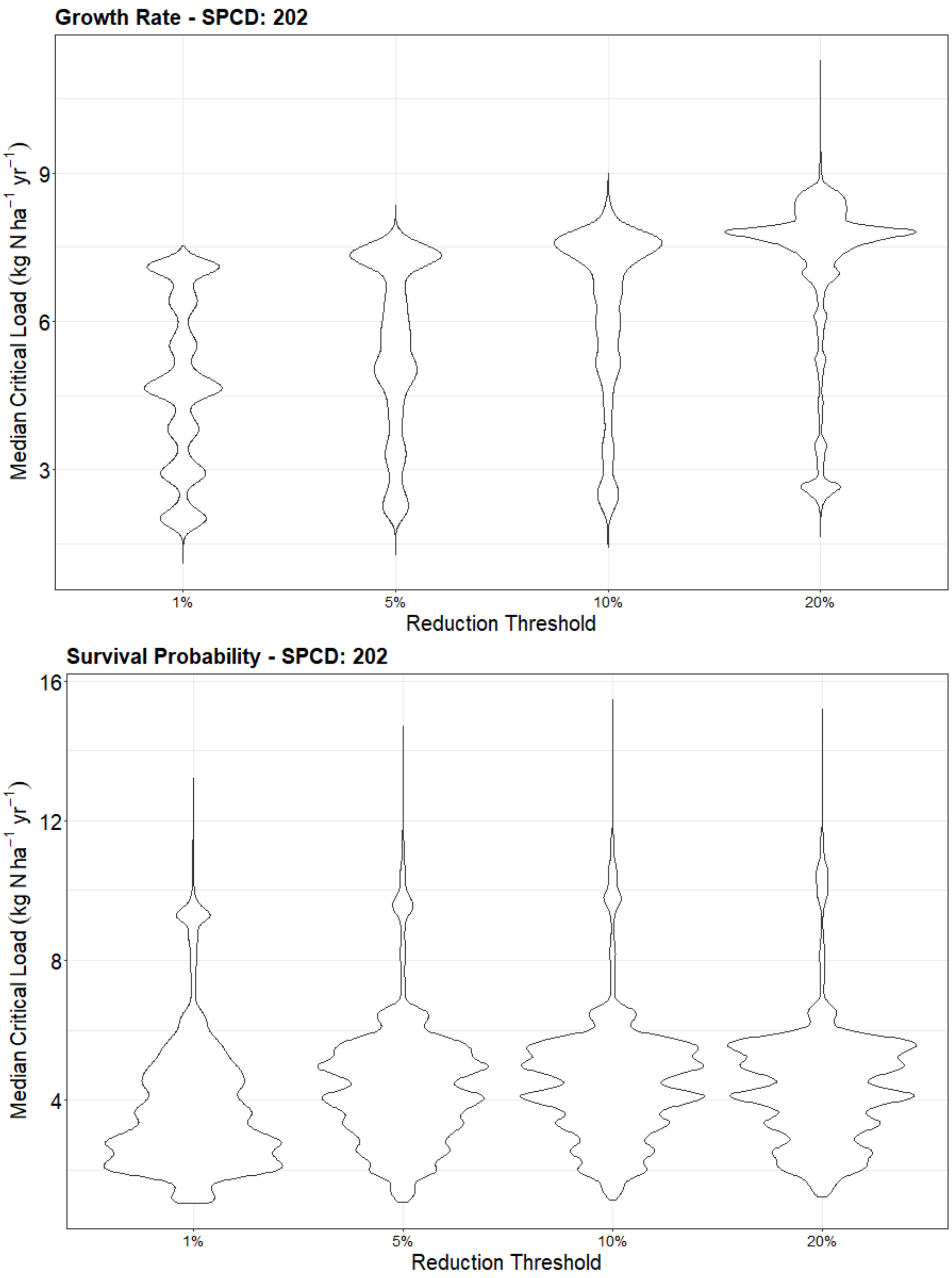


**Figure S3.** The bootstrap median N CL for Douglas-fir for (a) growth and (b) survival probability at different reduction thresholds (1%, 5%, 10%, and 20%). Violin plots reflect the distribution of points where wider areas have a higher density of points in that CL bin.

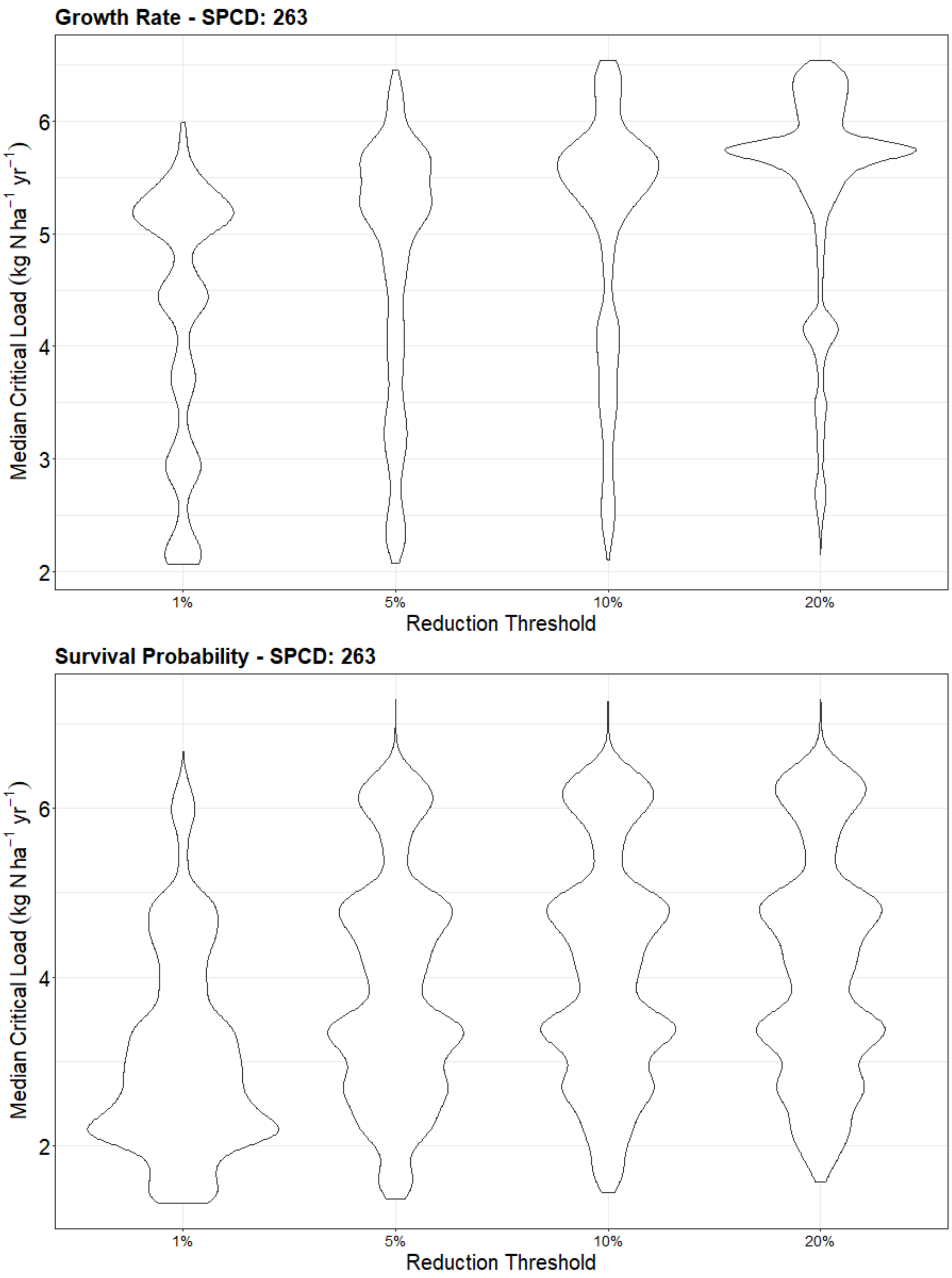


**Figure S4.** The bootstrap median N CL for western hemlock for (a) growth and (b) survival probability at different reduction thresholds (1%, 5%, 10%, and 20%). Violin plots reflect the distribution of points where wider areas have a higher density of points in that CL bin.

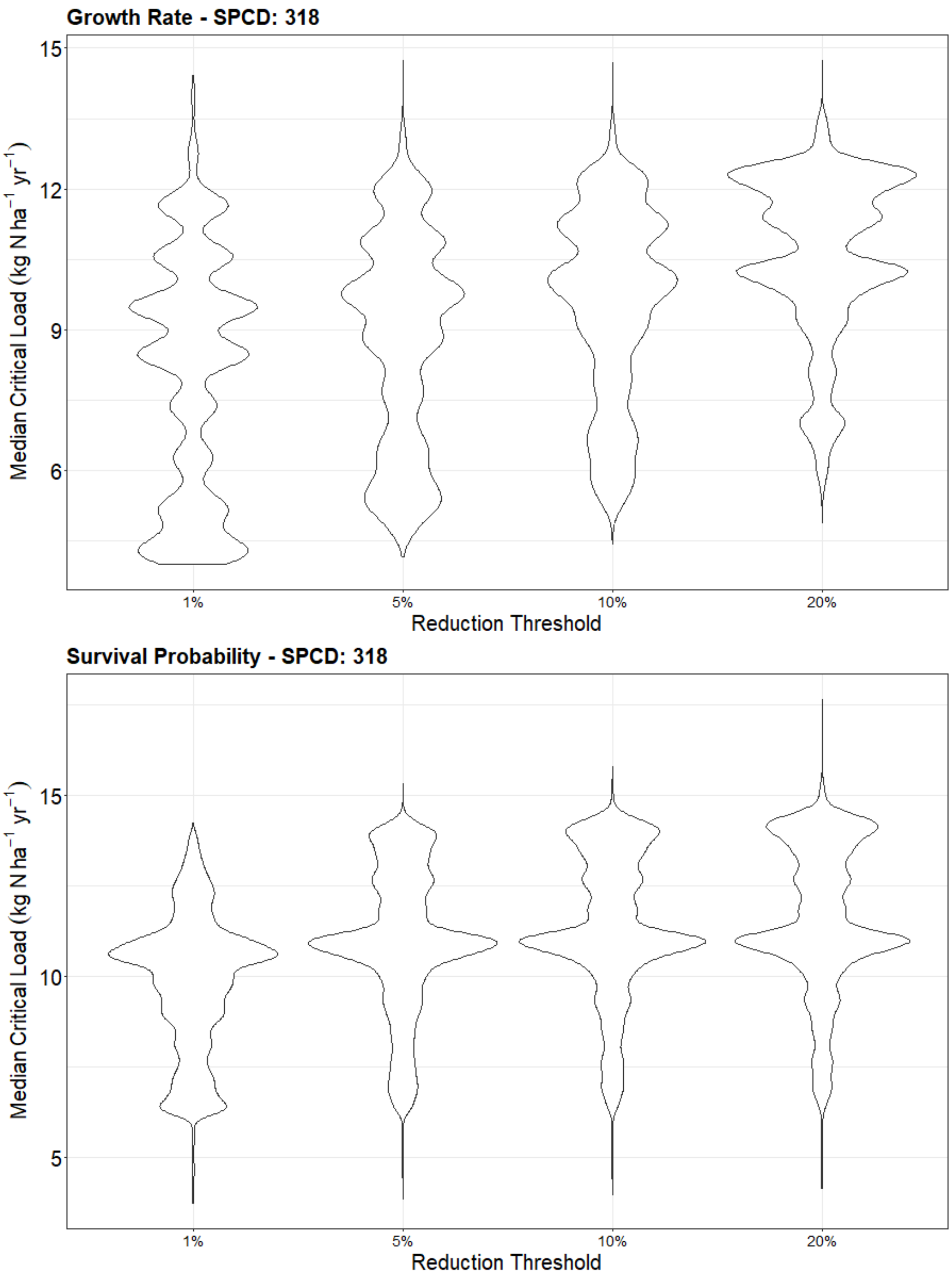


**Figure S5.** The bootstrap median N CL for sugar maple for (a) growth and (b) survival probability at different reduction thresholds (1%, 5%, 10%, and 20%). Violin plots reflect the distribution of points where wider areas have a higher density of points in that CL bin.

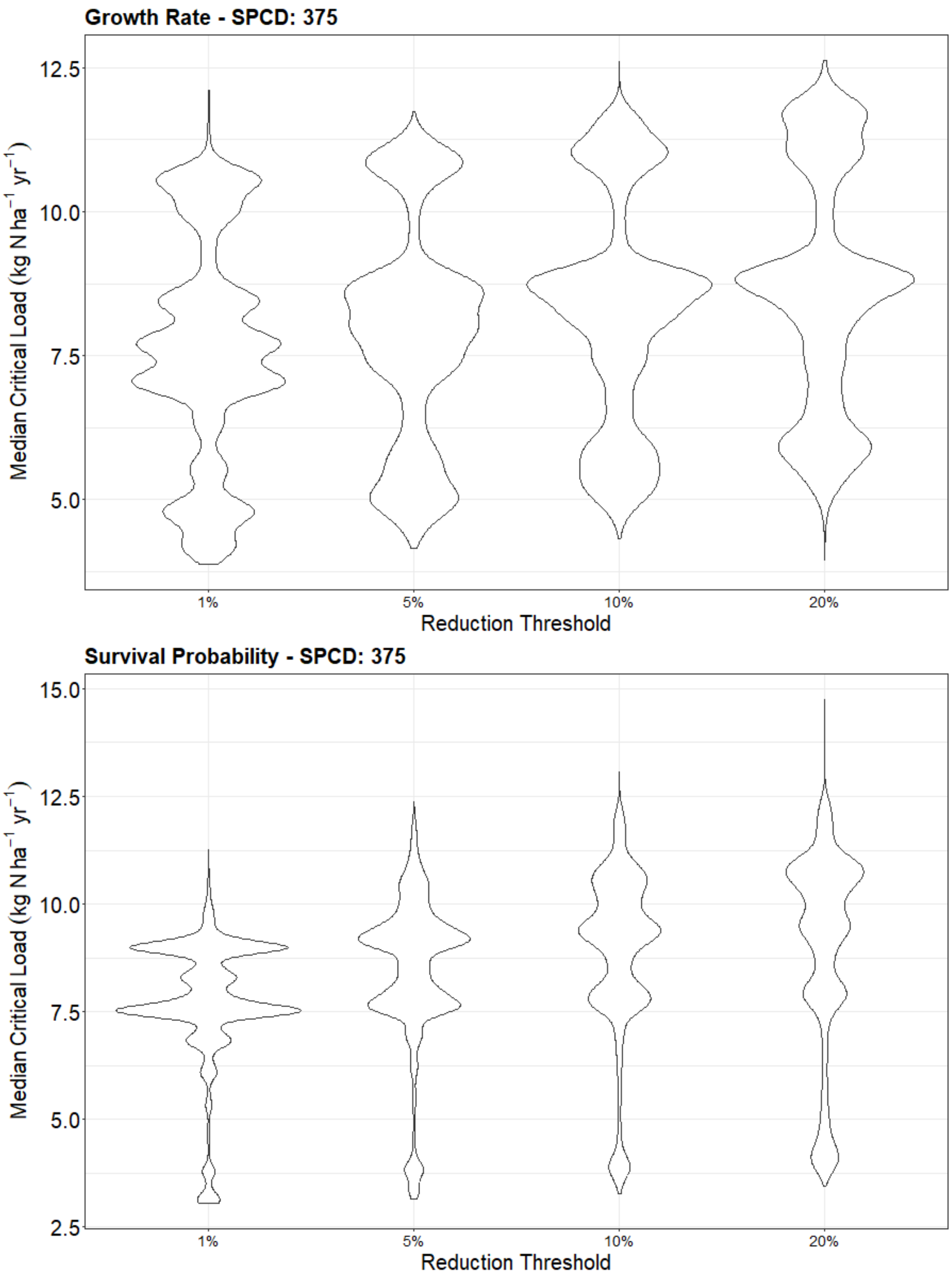


**Figure S6.** The bootstrap median N CL for paper birch for (a) growth and (b) survival probability at different reduction thresholds (1%, 5%, 10%, and 20%). Violin plots reflect the distribution of points where wider areas have a higher density of points in that CL bin.

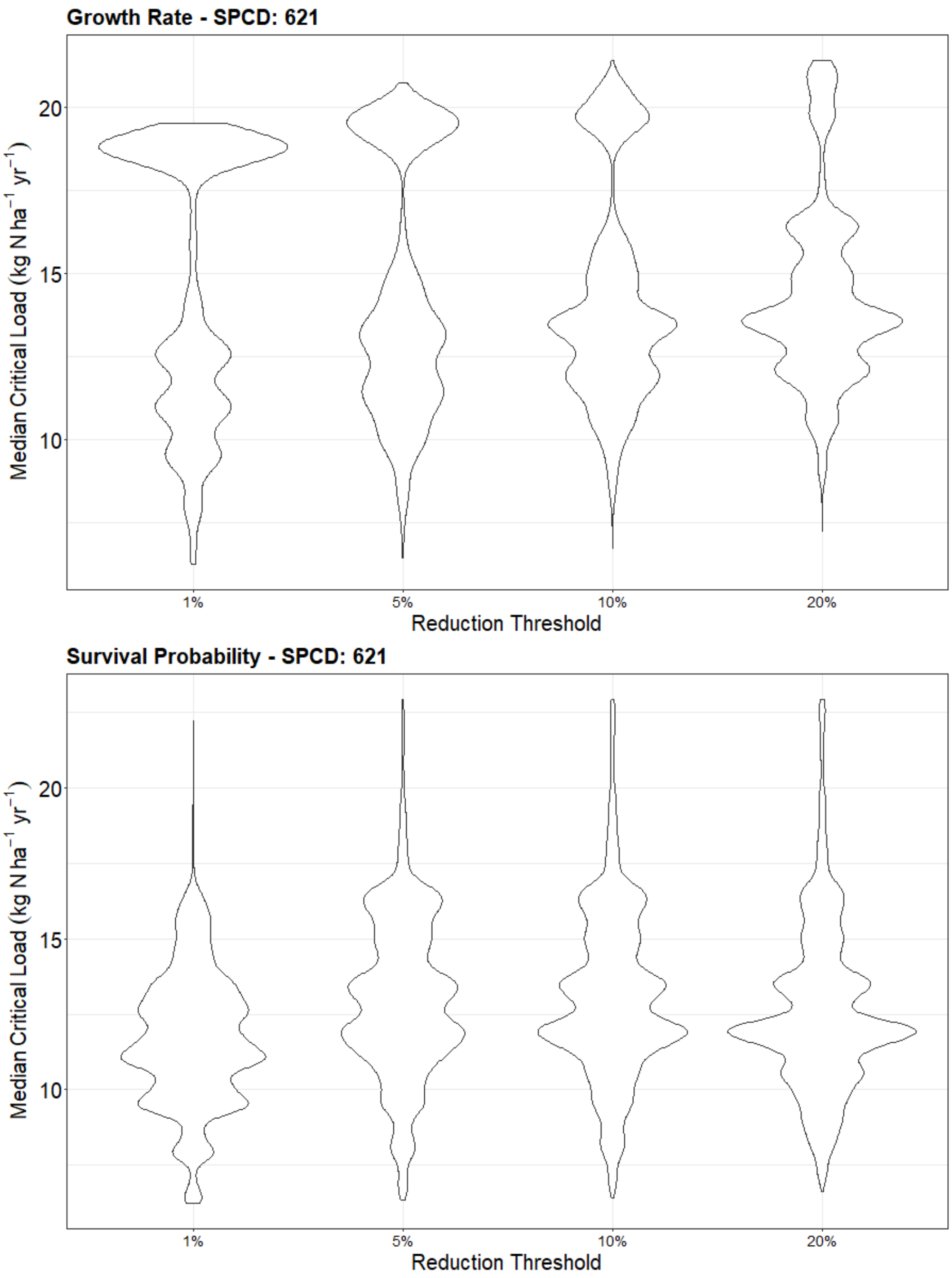


**Figure S7.** The bootstrap median N CL for yellow-poplar for (a) growth and (b) survival probability at different reduction thresholds (1%, 5%, 10%, and 20%). Violin plots reflect the distribution of points where wider areas have a higher density of points in that CL bin.

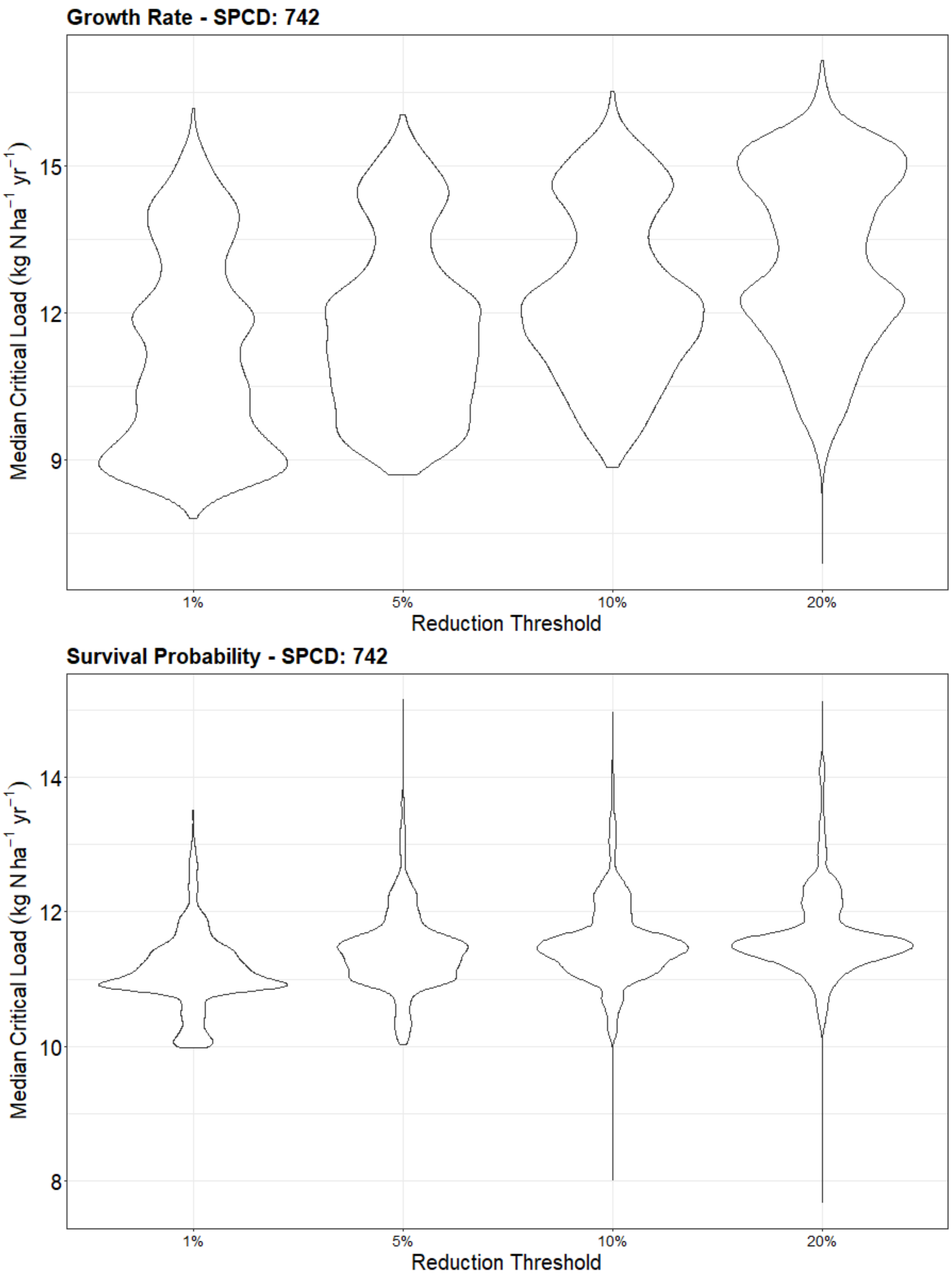


**Figure S8.** The bootstrap median N CL for eastern cottonwood for (a) growth and (b) survival probability at different reduction thresholds (1%, 5%, 10%, and 20%). Violin plots reflect the distribution of points where wider areas have a higher density of points in that CL bin.

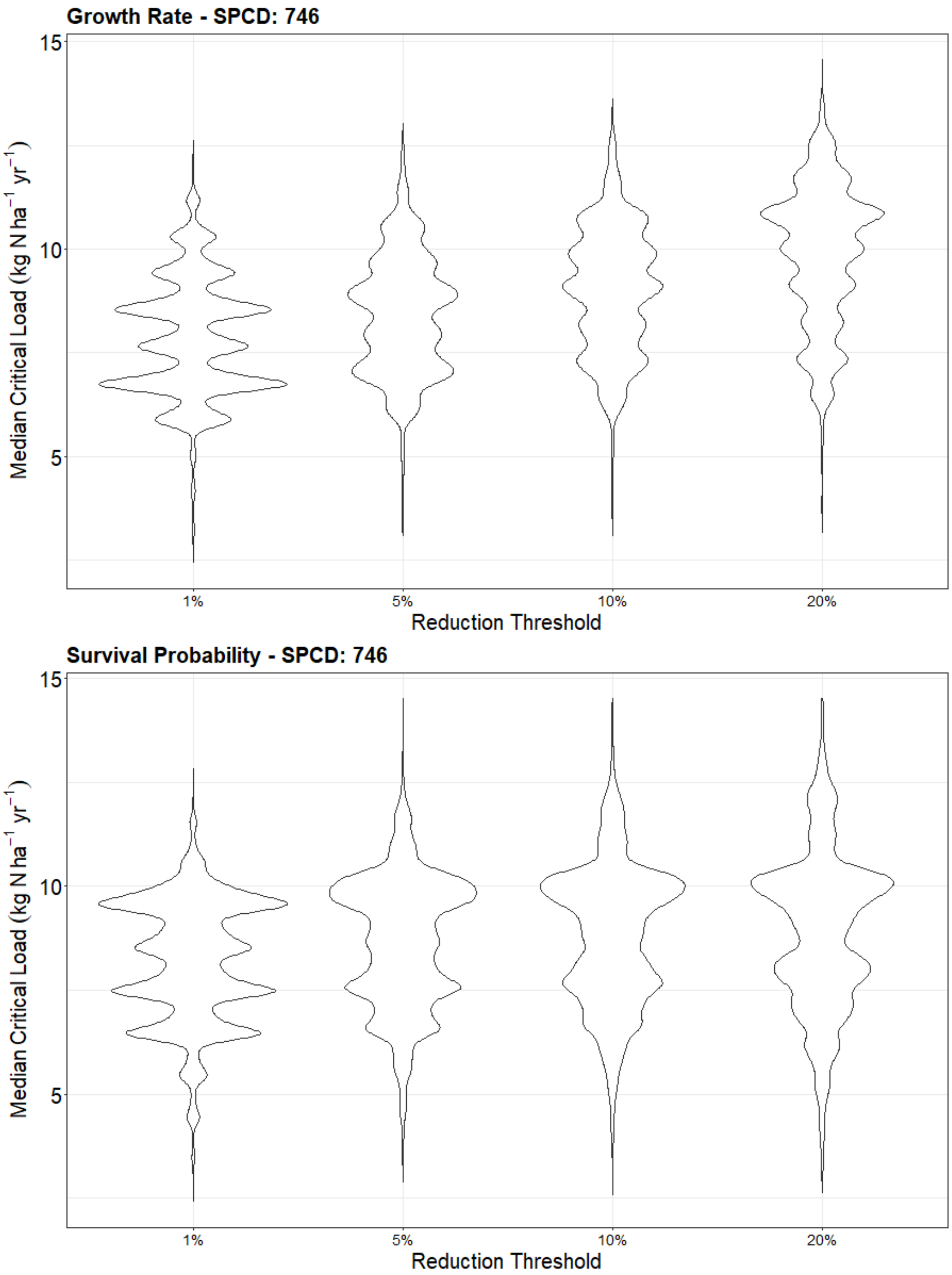


**Figure S9.** The bootstrap median N CL for quaking aspen for (a) growth and (b) survival probability at different reduction thresholds (1%, 5%, 10%, and 20%). Violin plots reflect the distribution of points where wider areas have a higher density of points in that CL bin.

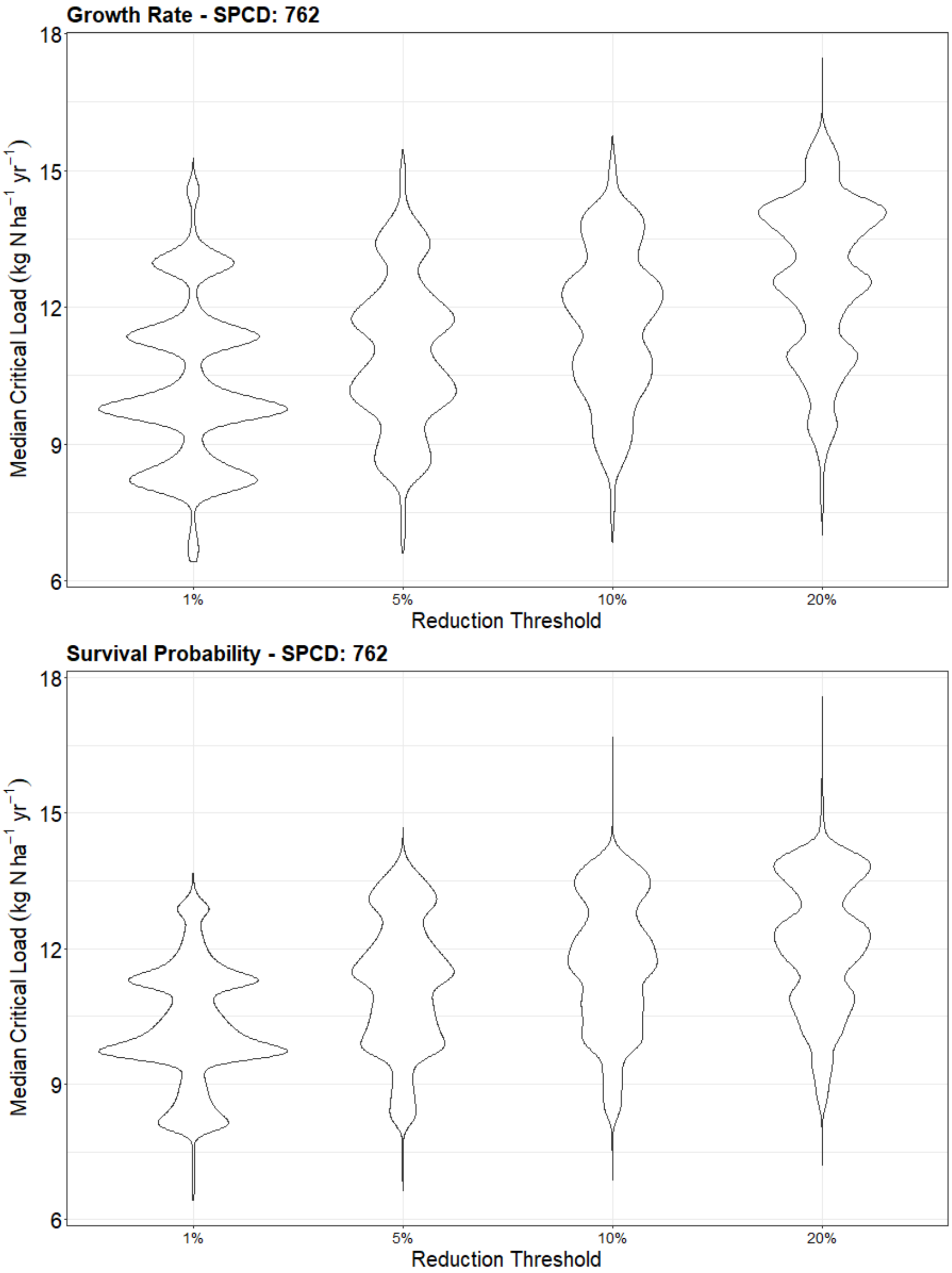


**Figure S10.** The bootstrap median N CL for black cherry for (a) growth and (b) survival probability at different reduction thresholds (1%, 5%, 10%, and 20%). Violin plots reflect the distribution of points where wider areas have a higher density of points in that CL bin.

# Additional Figures

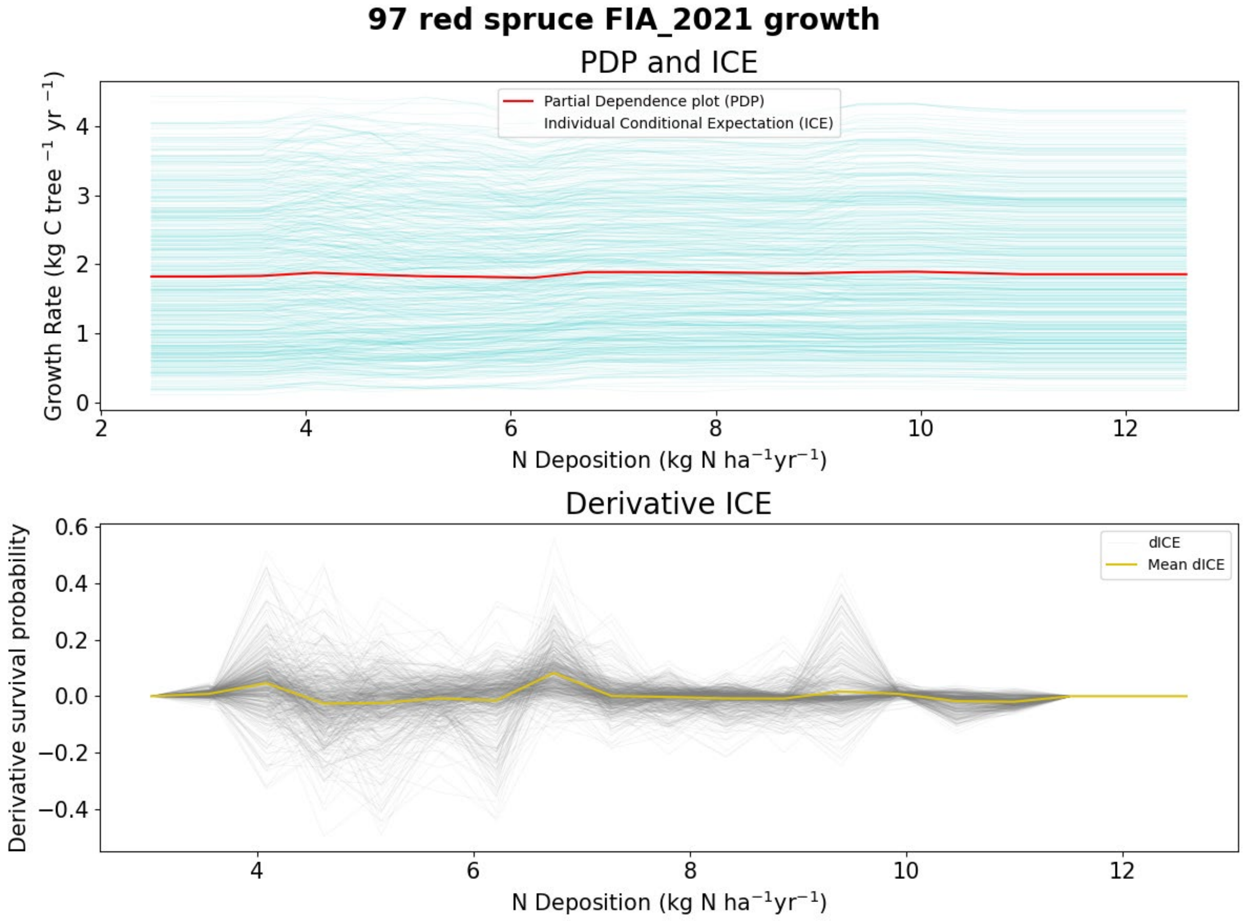


**Figure S11.** The growth rate ICE plot for all red spruce trees in the FIA dataset. Individual inventory trees are shown in (a) by the blue ICE lines while the partial dependence plot (PDP) response is shown in red. The derivative ICE (dICE), which shows the incremental change between N deposition bins, is shown in (b) with individual lines shown in grey and the mean dICE shown in yellow.

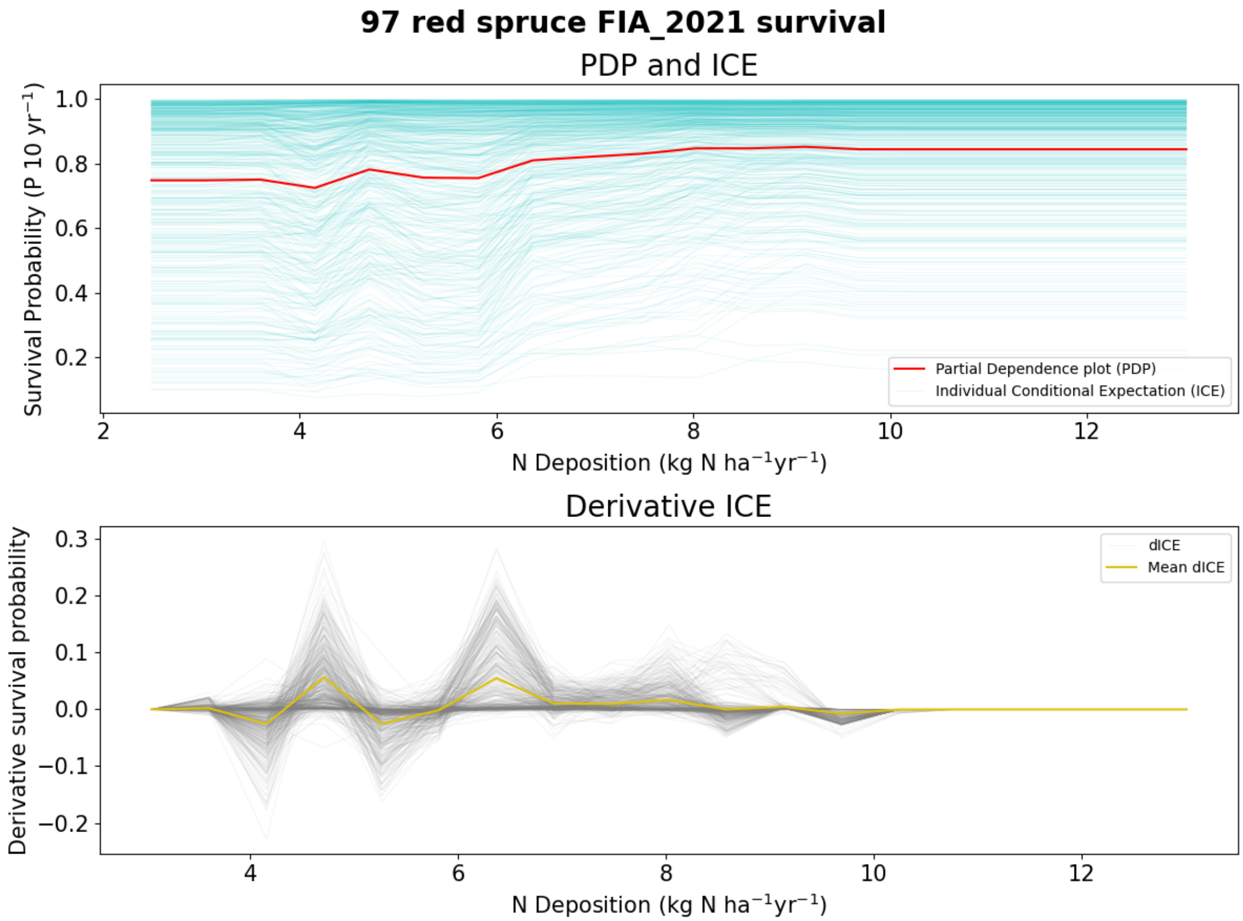


**Figure S12.** The survival probability ICE plot for all red spruce trees in the FIA dataset. Individual inventory trees are shown in (a) by the blue ICE lines while the partial dependence plot (PDP) response is shown in red. The derivative ICE (dICE), which shows the incremental change between N deposition bins, is shown in (b) with individual lines shown in grey and the mean dICE shown in yellow.

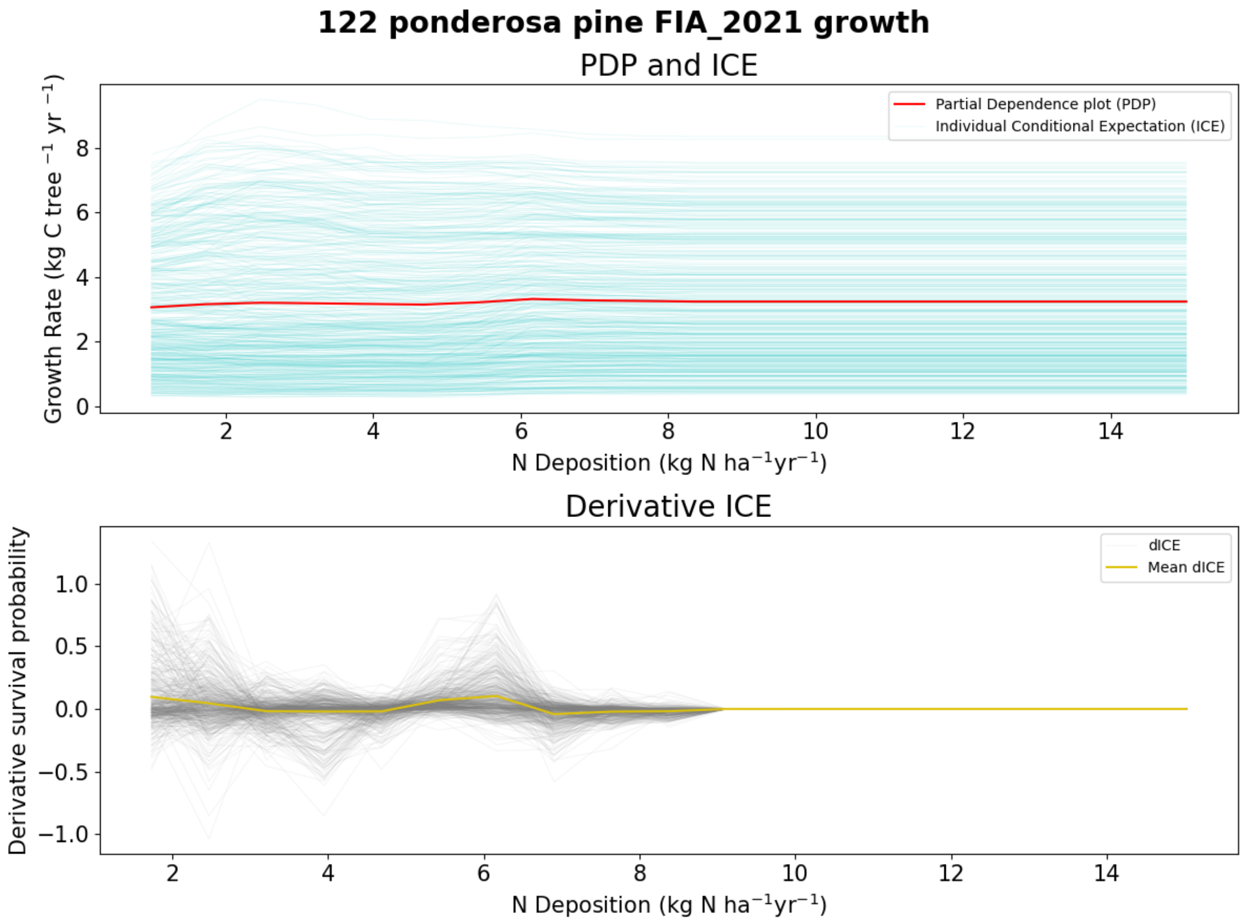


**Figure S13.** The growth rate ICE plot for all ponderosa pine trees in the FIA dataset. Individual inventory trees are shown in (a) by the blue ICE lines while the partial dependence plot (PDP) response is shown in red. The derivative ICE (dICE), which shows the incremental change between N deposition bins, is shown in (b) with individual lines shown in grey and the mean dICE shown in yellow.

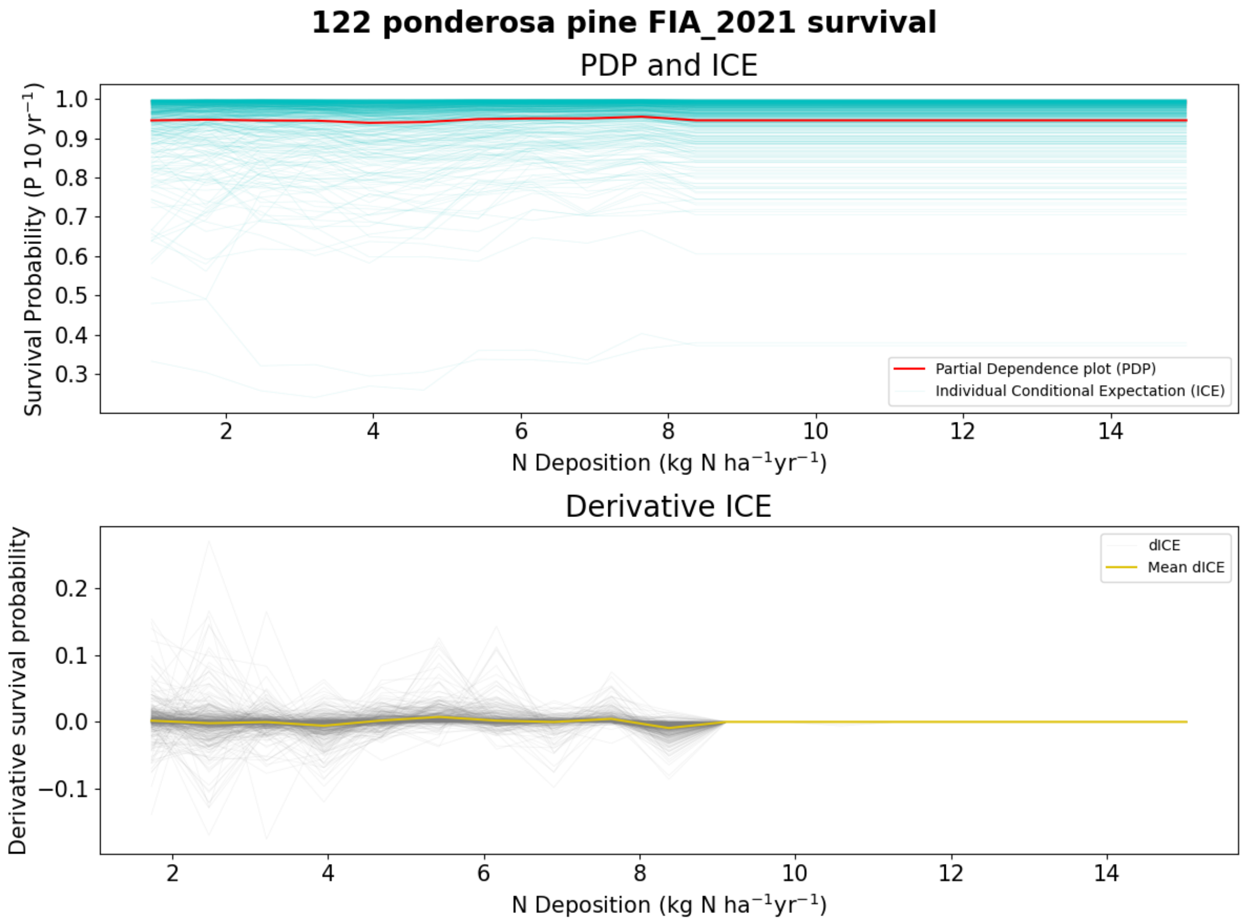


**Figure S14.** The survival probability ICE plot for all ponderosa pine trees in the FIA dataset. Individual inventory trees are shown in (a) by the blue ICE lines while the partial dependence plot (PDP) response is shown in red. The derivative ICE (dICE), which shows the incremental change between N deposition bins, is shown in (b) with individual lines shown in grey and the mean dICE shown in yellow.

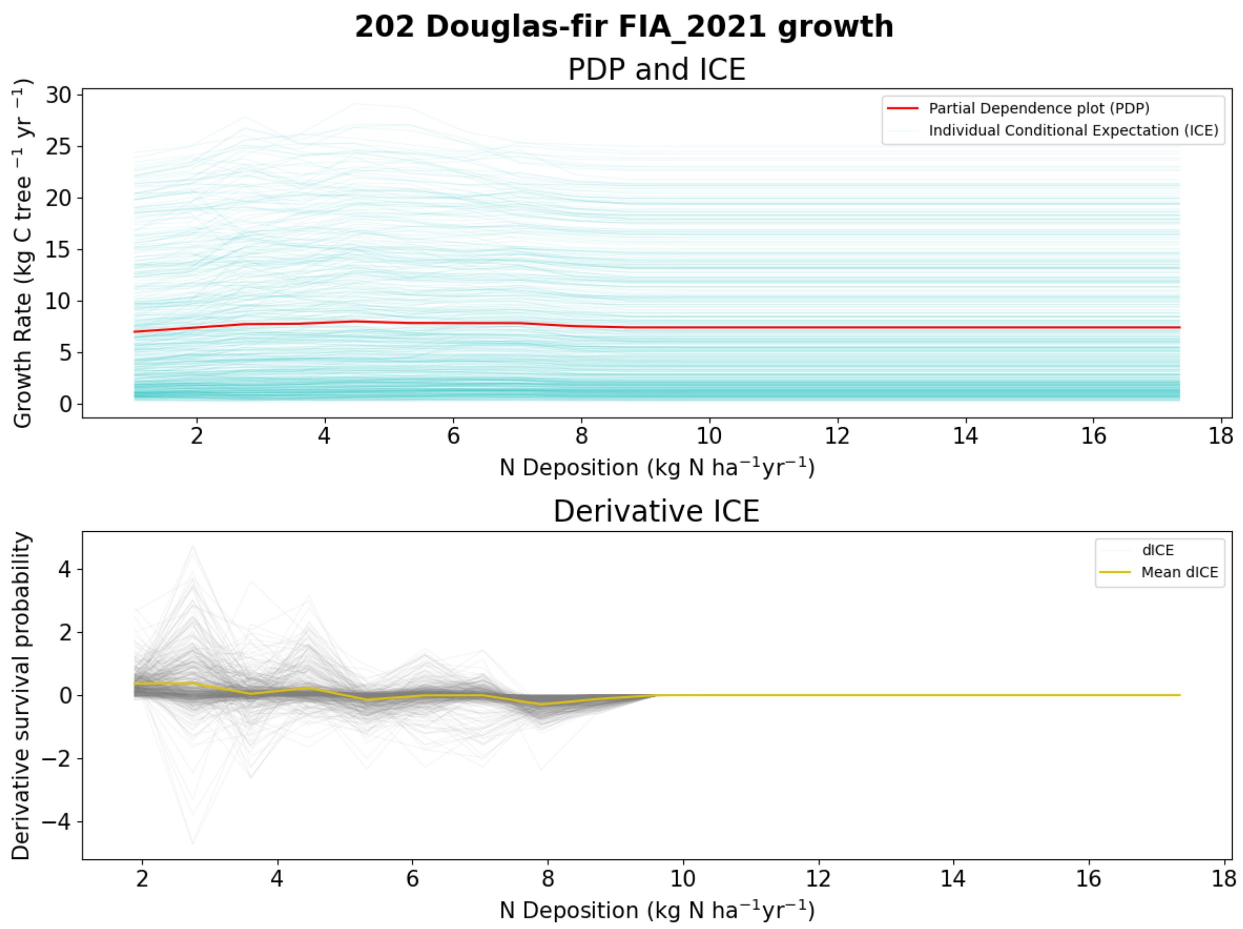


**Figure S15.** The growth rate ICE plot for all Douglas-fir trees in the FIA dataset. Individual inventory trees are shown in (a) by the blue ICE lines while the partial dependence plot (PDP) response is shown in red. The derivative ICE (dICE), which shows the incremental change between N deposition bins, is shown in (b) with individual lines shown in grey and the mean dICE shown in yellow.

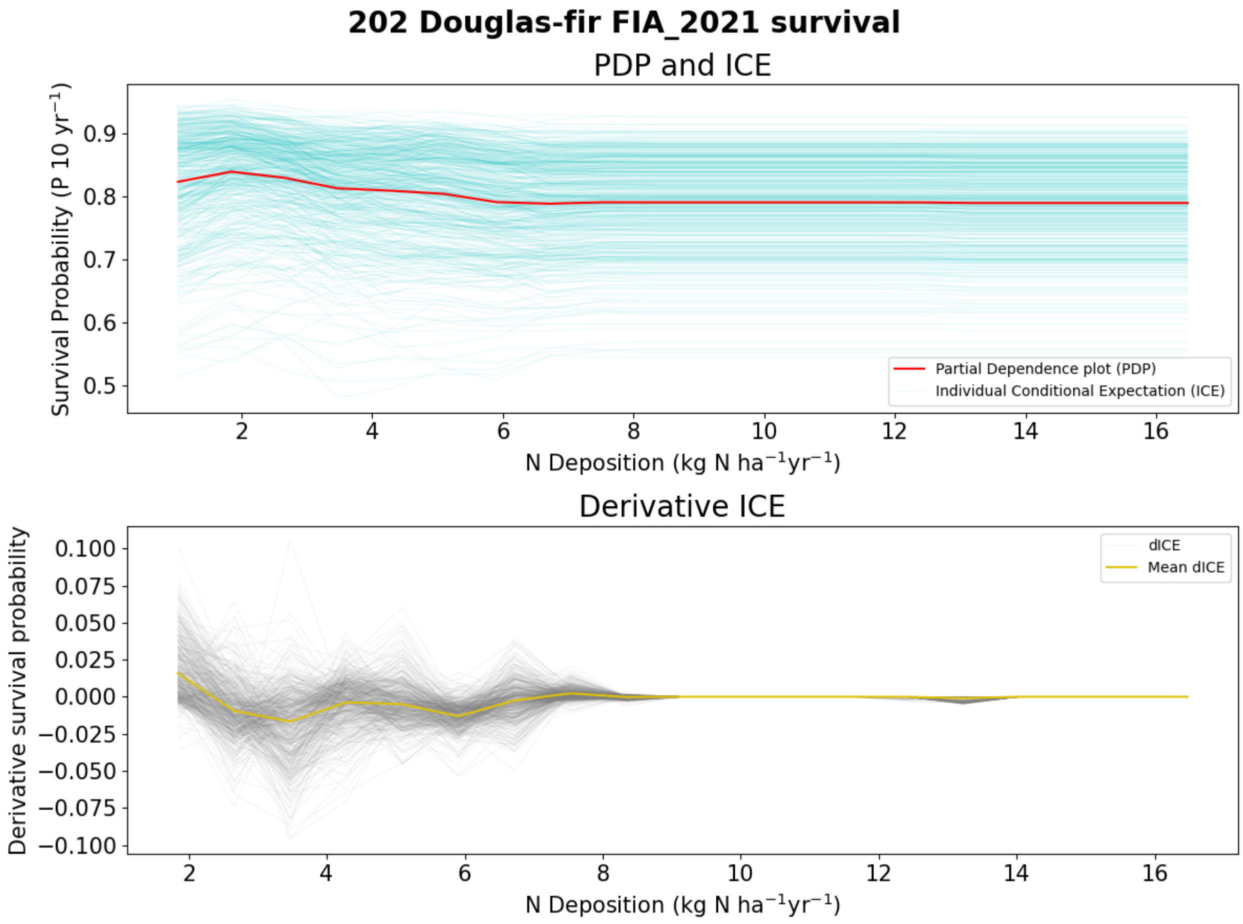


**Figure S16.** The survival probability ICE plot for all Douglas-fir trees in the FIA dataset. Individual inventory trees are shown in (a) by the blue ICE lines while the partial dependence plot (PDP) response is shown in red. The derivative ICE (dICE), which shows the incremental change between N deposition bins, is shown in (b) with individual lines shown in grey and the mean dICE shown in yellow.

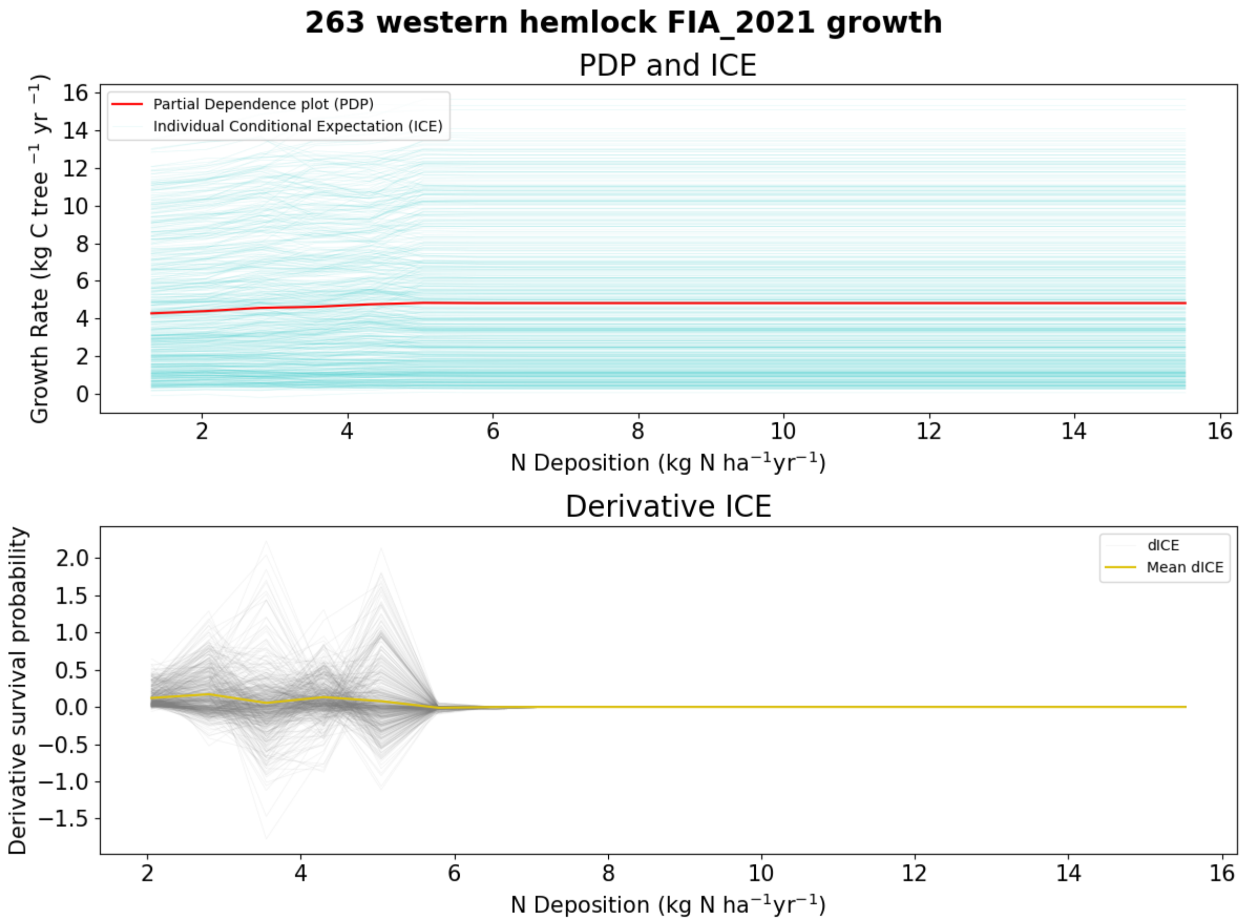


**Figure S17.** The growth rate ICE plot for all western hemlock trees in the FIA dataset. Individual inventory trees are shown in (a) by the blue ICE lines while the partial dependence plot (PDP) response is shown in red. The derivative ICE (dICE), which shows the incremental change between N deposition bins, is shown in (b) with individual lines shown in grey and the mean dICE shown in yellow.

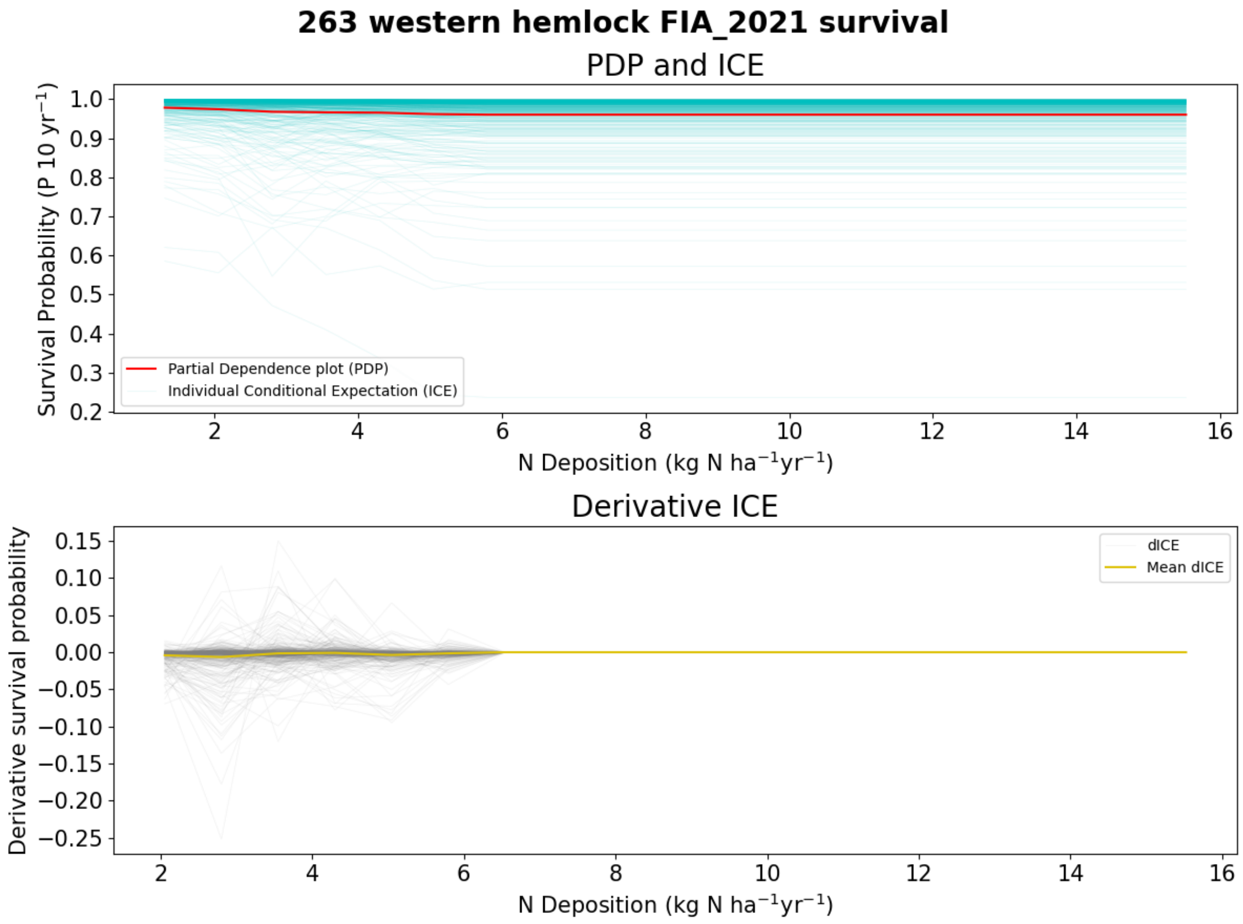


**Figure S18.** The survival probability ICE plot for all western hemlock trees in the FIA dataset. Individual inventory trees are shown in (a) by the blue ICE lines while the partial dependence plot (PDP) response is shown in red. The derivative ICE (dICE), which shows the incremental change between N deposition bins, is shown in (b) with individual lines shown in grey and the mean dICE shown in yellow.

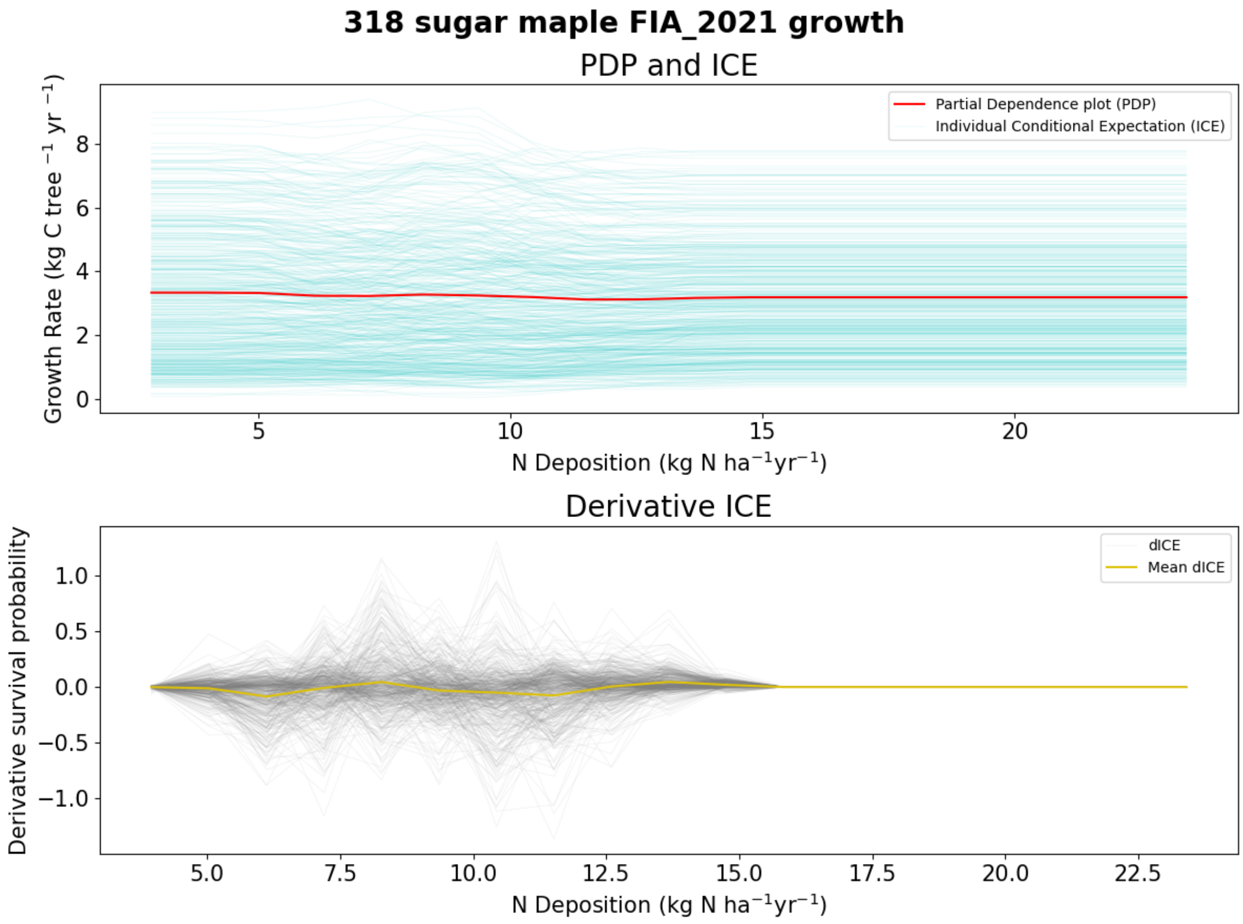


**Figure S19.** The growth rate ICE plot for all sugar maple trees in the FIA dataset. Individual inventory trees are shown in (a) by the blue ICE lines while the partial dependence plot (PDP) response is shown in red. The derivative ICE (dICE), which shows the incremental change between N deposition bins, is shown in (b) with individual lines shown in grey and the mean dICE shown in yellow.

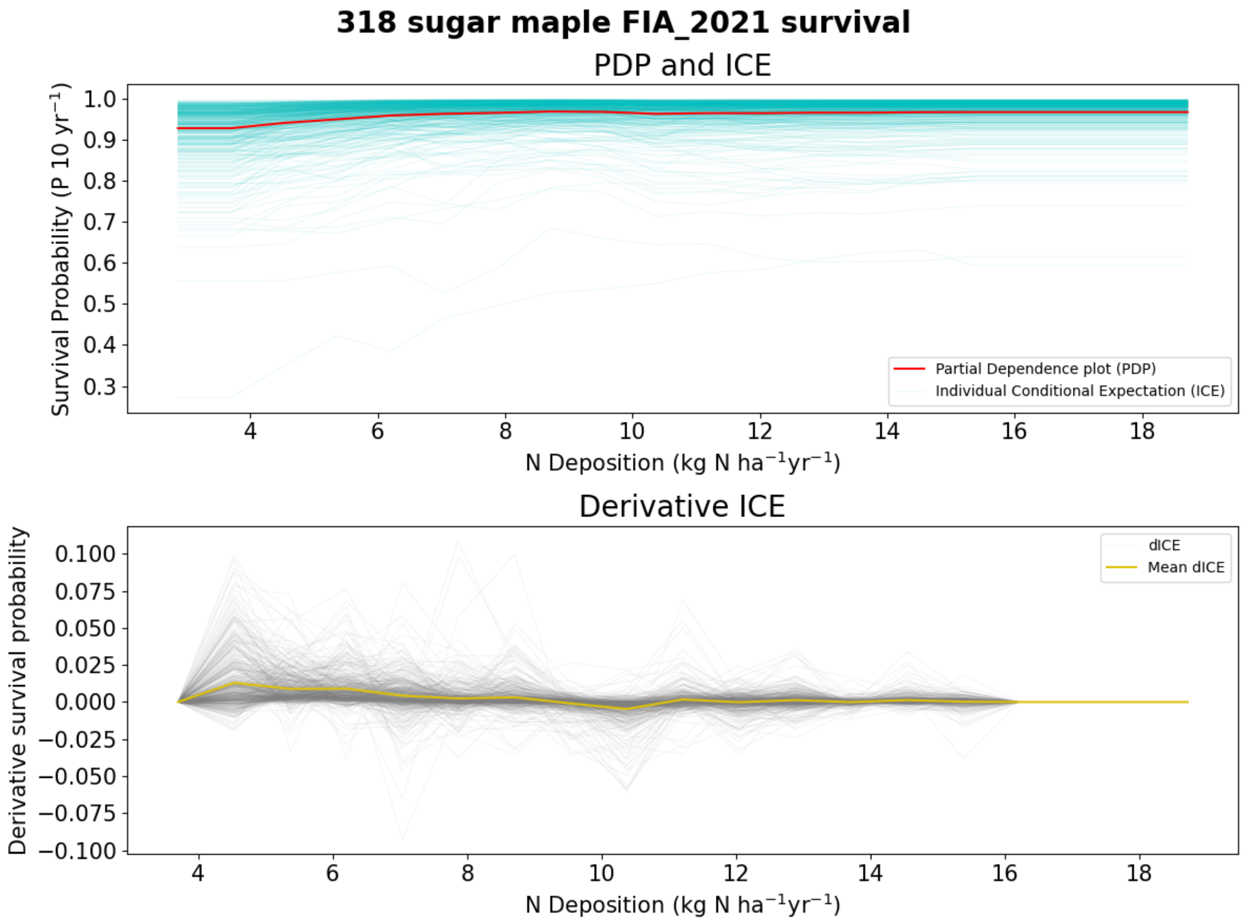


**Figure S20.** The survival probability ICE plot for all sugar maple trees in the FIA dataset. Individual inventory trees are shown in (a) by the blue ICE lines while the partial dependence plot (PDP) response is shown in red. The derivative ICE (dICE), which shows the incremental change between N deposition bins, is shown in (b) with individual lines shown in grey and the mean dICE shown in yellow.

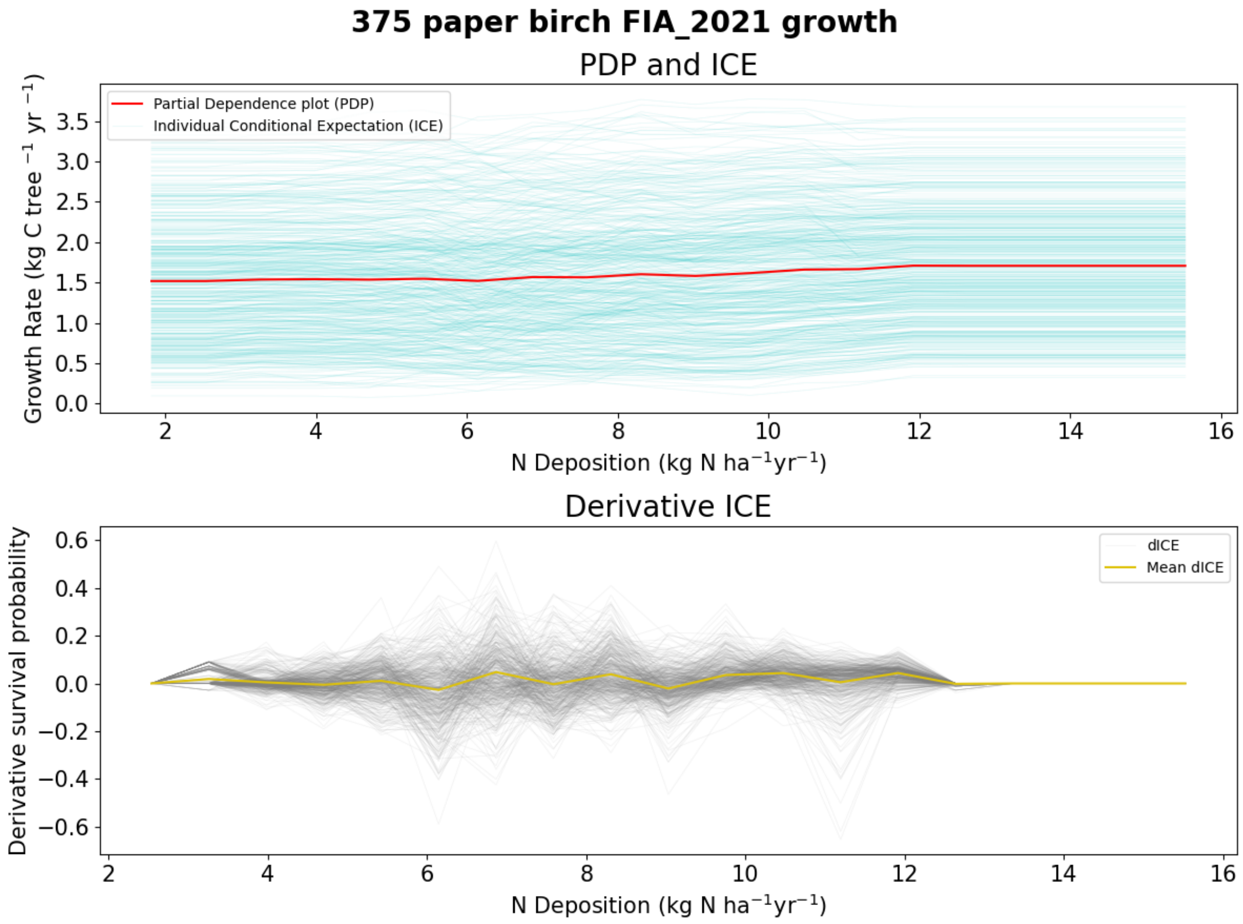


**Figure S21.** The growth rate ICE plot for all paper birch trees in the FIA dataset. Individual inventory trees are shown in (a) by the blue ICE lines while the partial dependence plot (PDP) response is shown in red. The derivative ICE (dICE), which shows the incremental change between N deposition bins, is shown in (b) with individual lines shown in grey and the mean dICE shown in yellow.

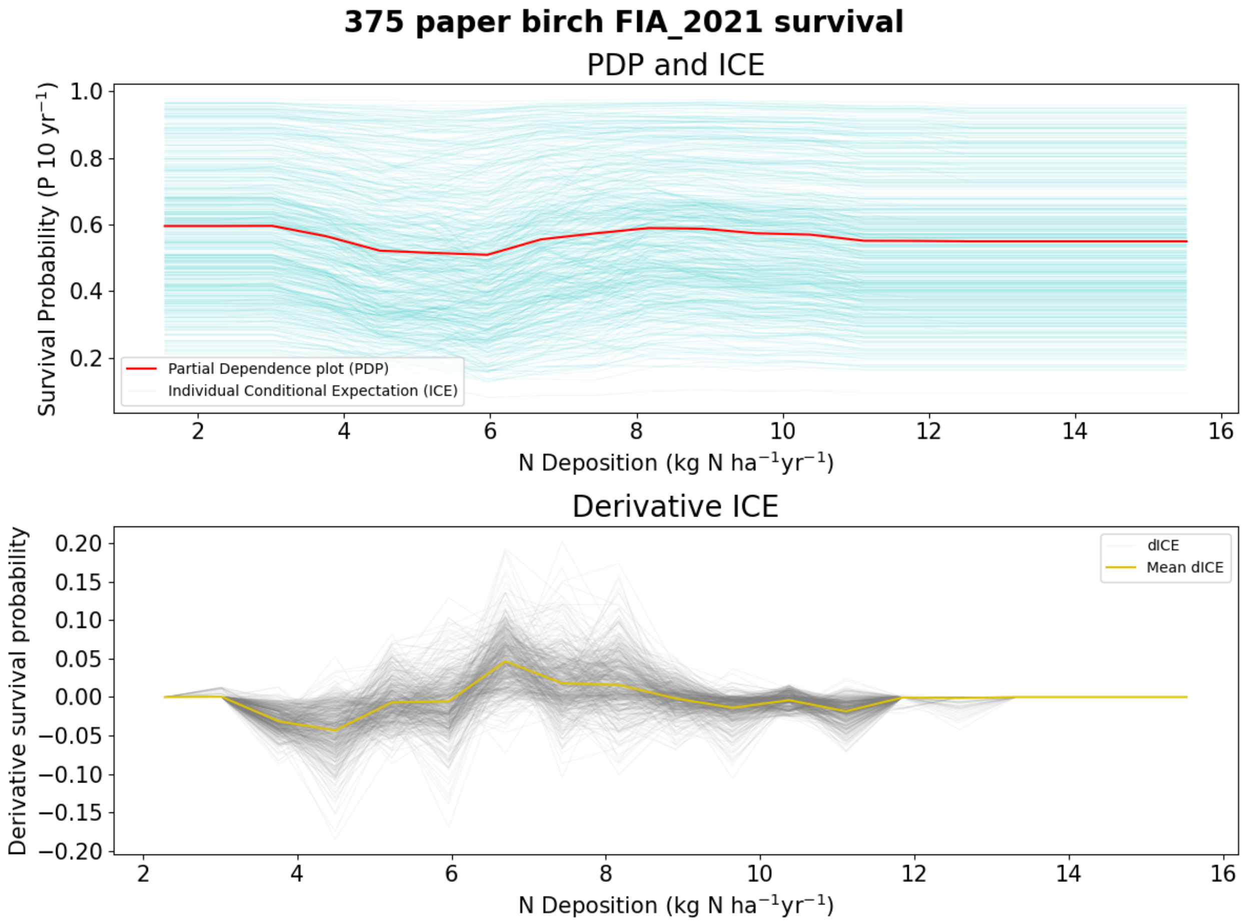


**Figure S22.** The survival probability ICE plot for all paper birch trees in the FIA dataset. Individual inventory trees are shown in (a) by the blue ICE lines while the partial dependence plot (PDP) response is shown in red. The derivative ICE (dICE), which shows the incremental change between N deposition bins, is shown in (b) with individual lines shown in grey and the mean dICE shown in yellow.

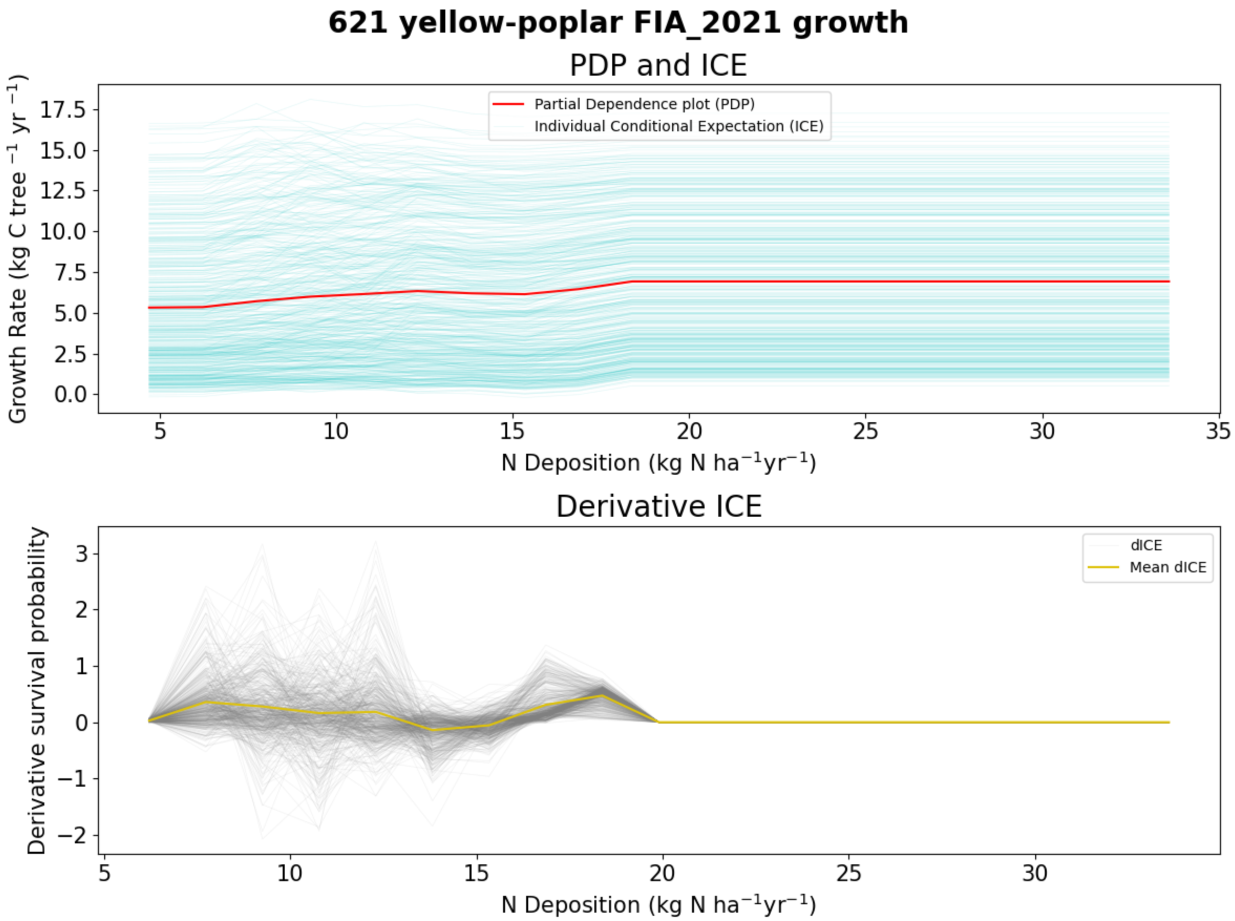


**Figure S23.** The growth rate ICE plot for all yellow-poplar trees in the FIA dataset. Individual inventory trees are shown in (a) by the blue ICE lines while the partial dependence plot (PDP) response is shown in red. The derivative ICE (dICE), which shows the incremental change between N deposition bins, is shown in (b) with individual lines shown in grey and the mean dICE shown in yellow.

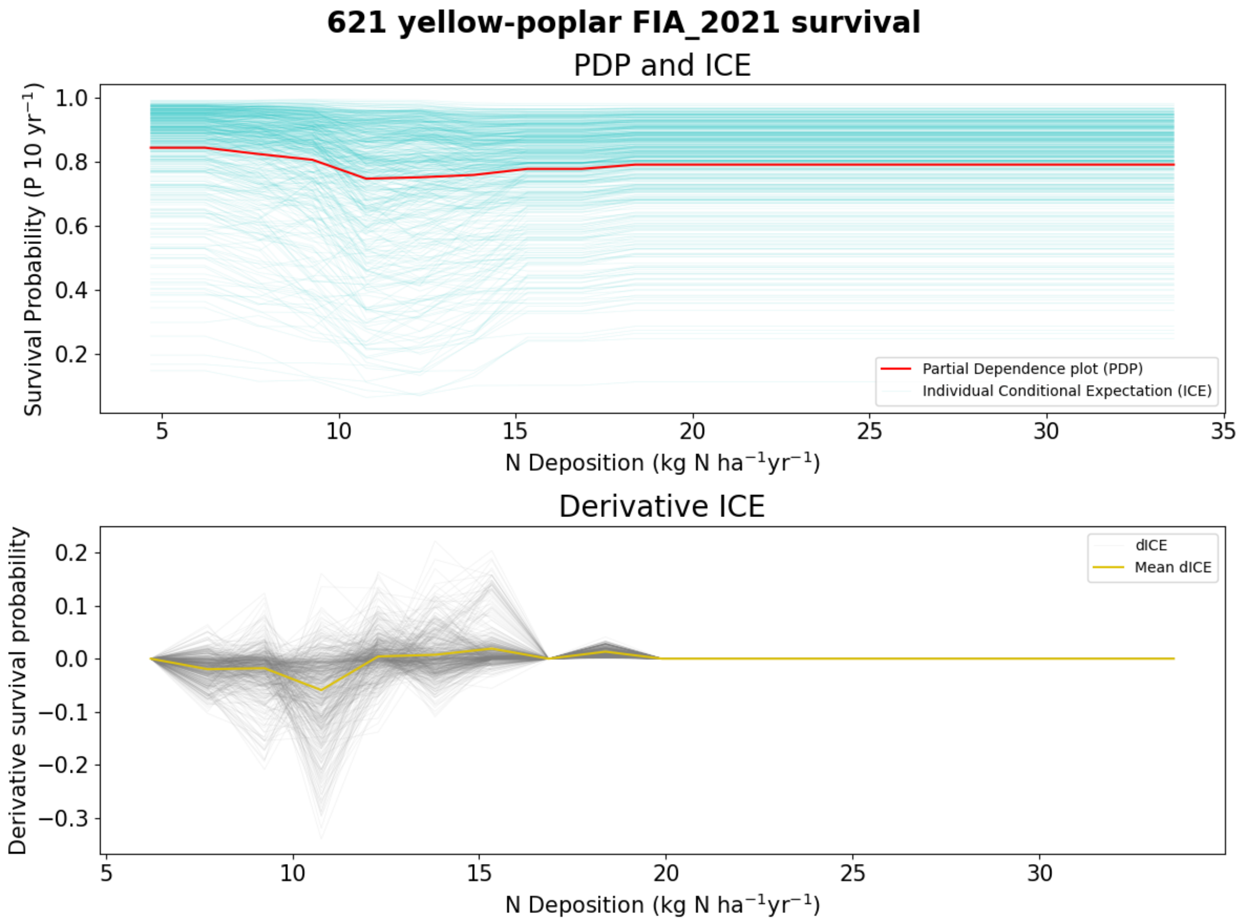


**Figure S24.** The survival probability ICE plot for all yellow-poplar trees in the FIA dataset. Individual inventory trees are shown in (a) by the blue ICE lines while the partial dependence plot (PDP) response is shown in red. The derivative ICE (dICE), which shows the incremental change between N deposition bins, is shown in (b) with individual lines shown in grey and the mean dICE shown in yellow.

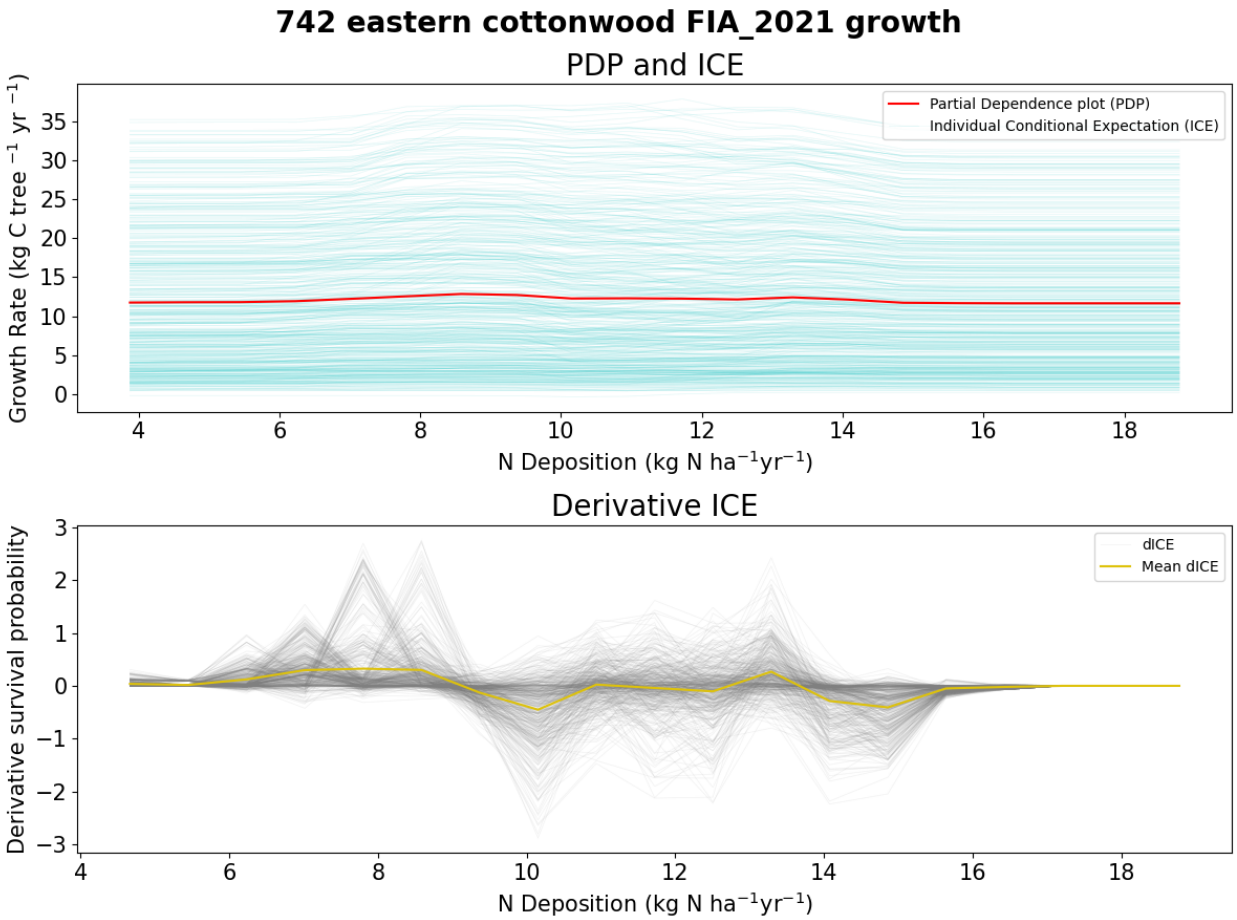


**Figure S25.** The growth rate ICE plot for all eastern cottonwood trees in the FIA dataset. Individual inventory trees are shown in (a) by the blue ICE lines while the partial dependence plot (PDP) response is shown in red. The derivative ICE (dICE), which shows the incremental change between N deposition bins, is shown in (b) with individual lines shown in grey and the mean dICE shown in yellow.

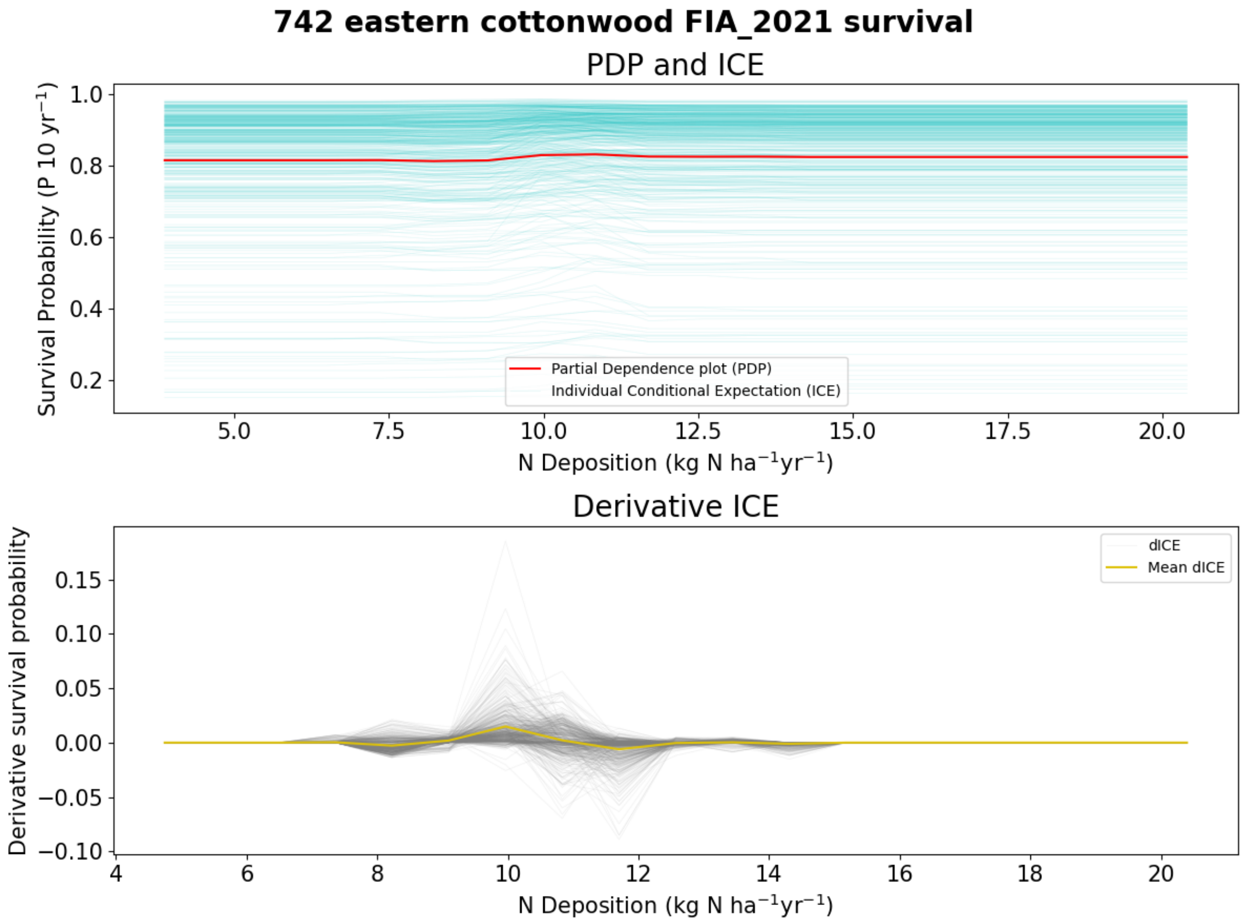


**Figure S26.** The survival probability ICE plot for all eastern cottonwood trees in the FIA dataset. Individual inventory trees are shown in (a) by the blue ICE lines while the partial dependence plot (PDP) response is shown in red. The derivative ICE (dICE), which shows the incremental change between N deposition bins, is shown in (b) with individual lines shown in grey and the mean dICE shown in yellow.

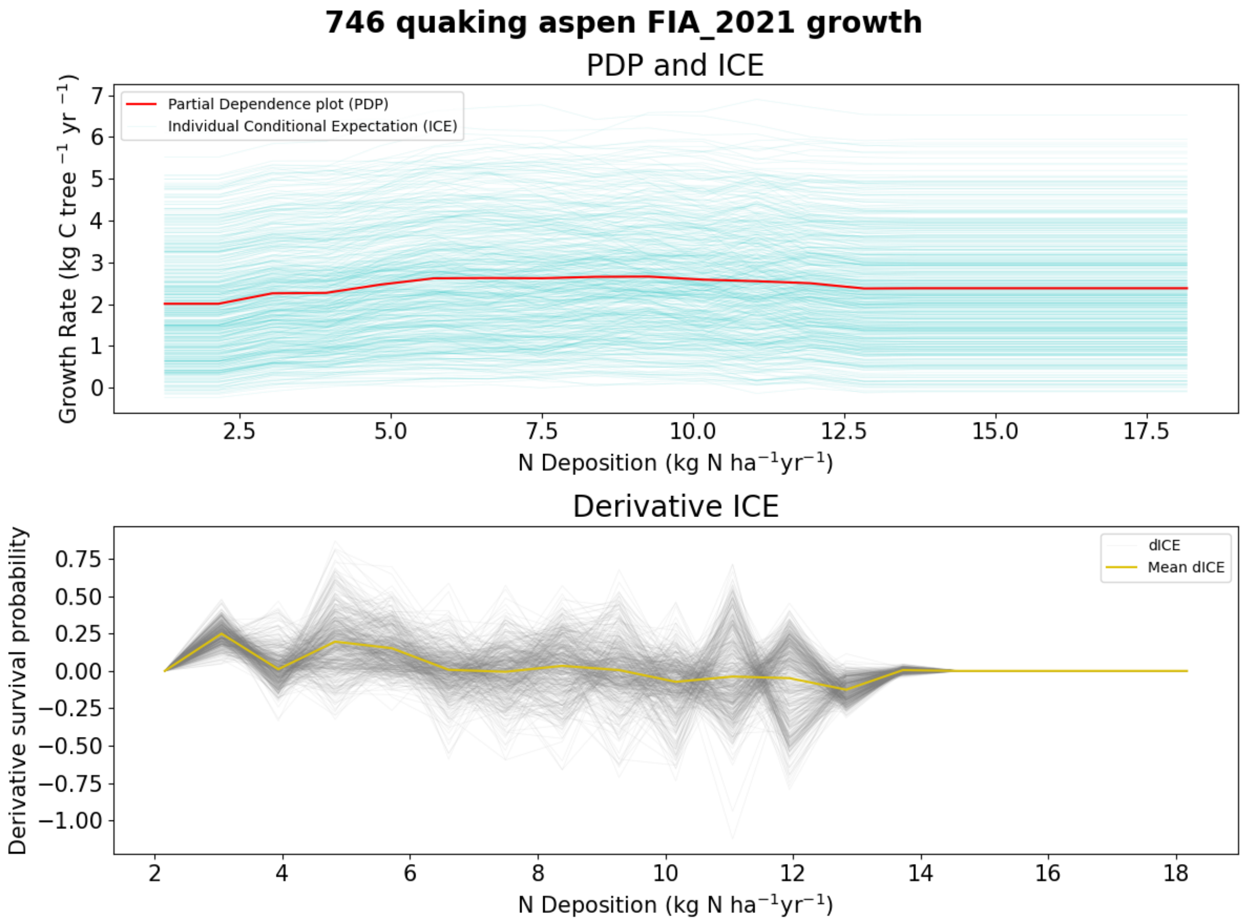


**Figure S27.** The growth rate ICE plot for all quaking aspen trees in the FIA dataset. Individual inventory trees are shown in (a) by the blue ICE lines while the partial dependence plot (PDP) response is shown in red. The derivative ICE (dICE), which shows the incremental change between N deposition bins, is shown in (b) with individual lines shown in grey and the mean dICE shown in yellow.

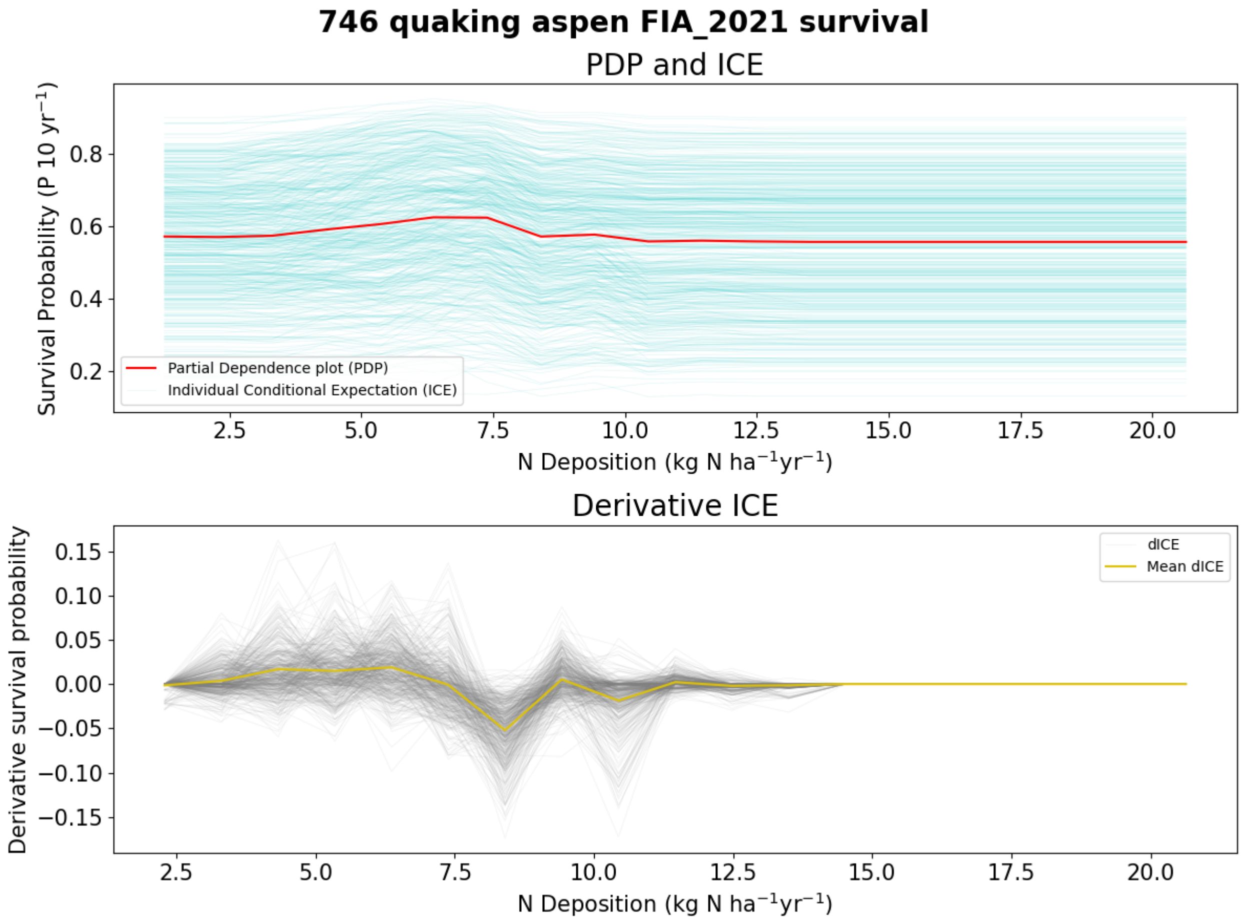


**Figure S28.** The survival probability ICE plot for all quaking aspen trees in the FIA dataset. Individual inventory trees are shown in (a) by the blue ICE lines while the partial dependence plot (PDP) response is shown in red. The derivative ICE (dICE), which shows the incremental change between N deposition bins, is shown in (b) with individual lines shown in grey and the mean dICE shown in yellow.

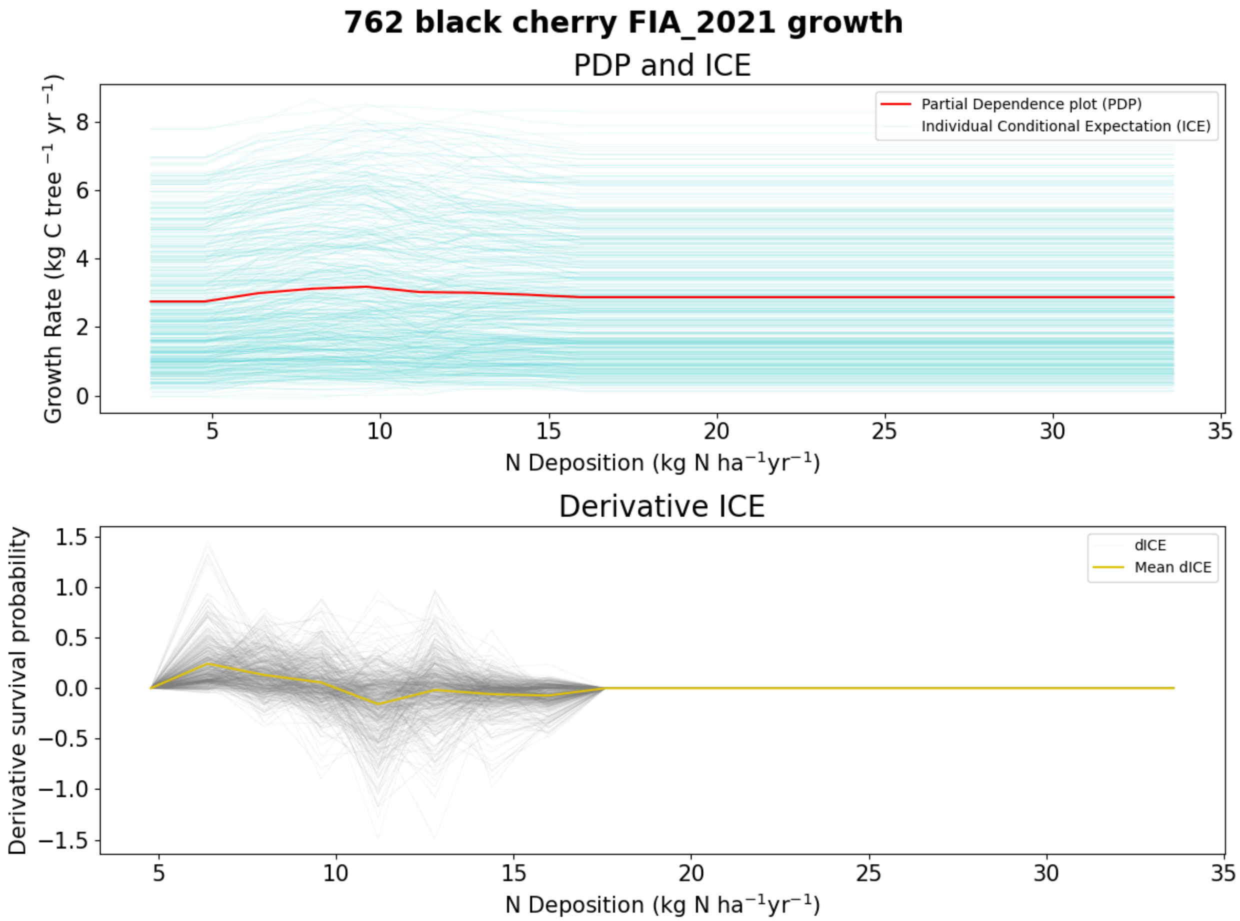


**Figure S29.** The growth rate ICE plot for all black cherry trees in the FIA dataset. Individual inventory trees are shown in (a) by the blue ICE lines while the partial dependence plot (PDP) response is shown in red. The derivative ICE (dICE), which shows the incremental change between N deposition bins, is shown in (b) with individual lines shown in grey and the mean dICE shown in yellow.

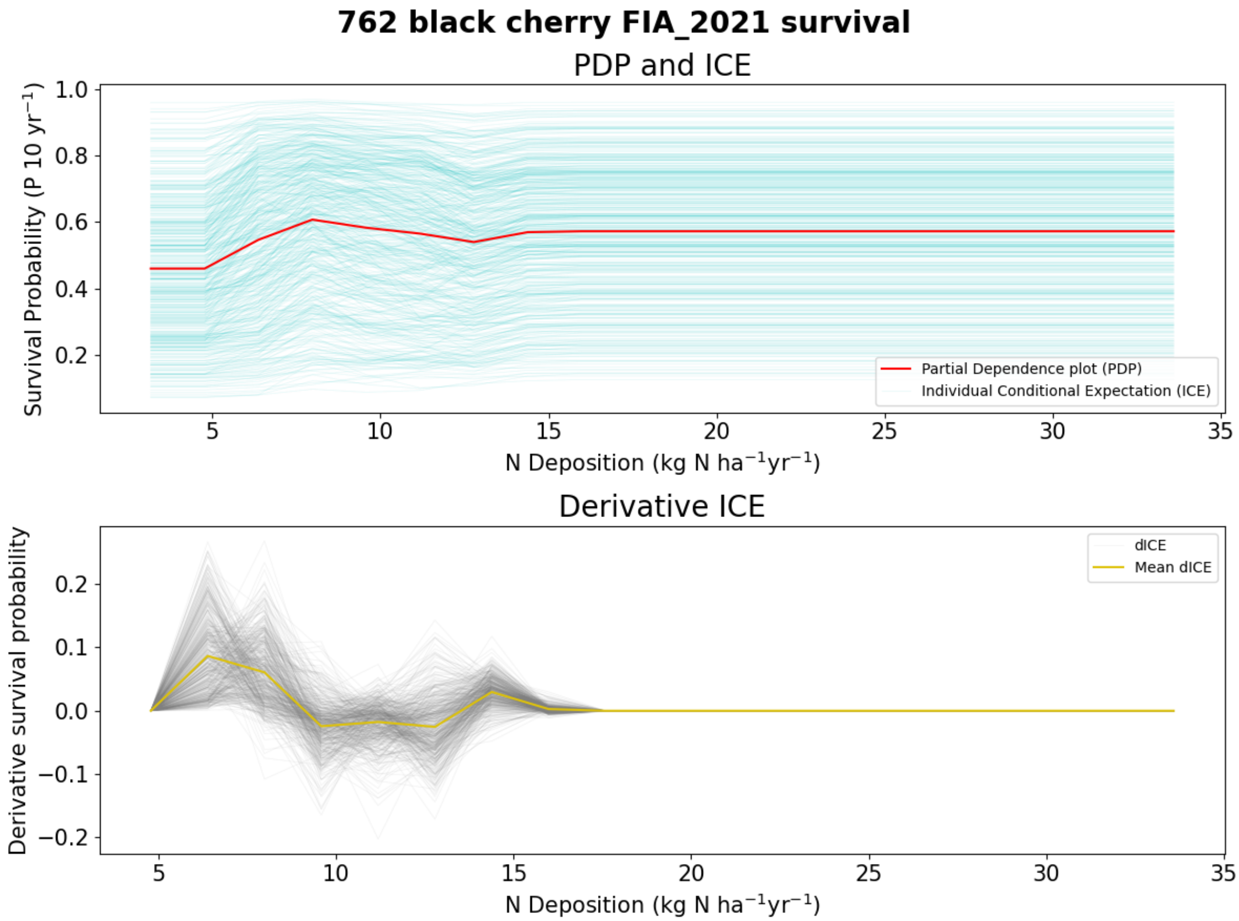


**Figure S30.** The survival probability ICE plot for all black cherry trees in the FIA dataset. Individual inventory trees are shown in (a) by the blue ICE lines while the partial dependence plot (PDP) response is shown in red. The derivative ICE (dICE), which shows the incremental change between N deposition bins, is shown in (b) with individual lines shown in grey and the mean dICE shown in yellow.

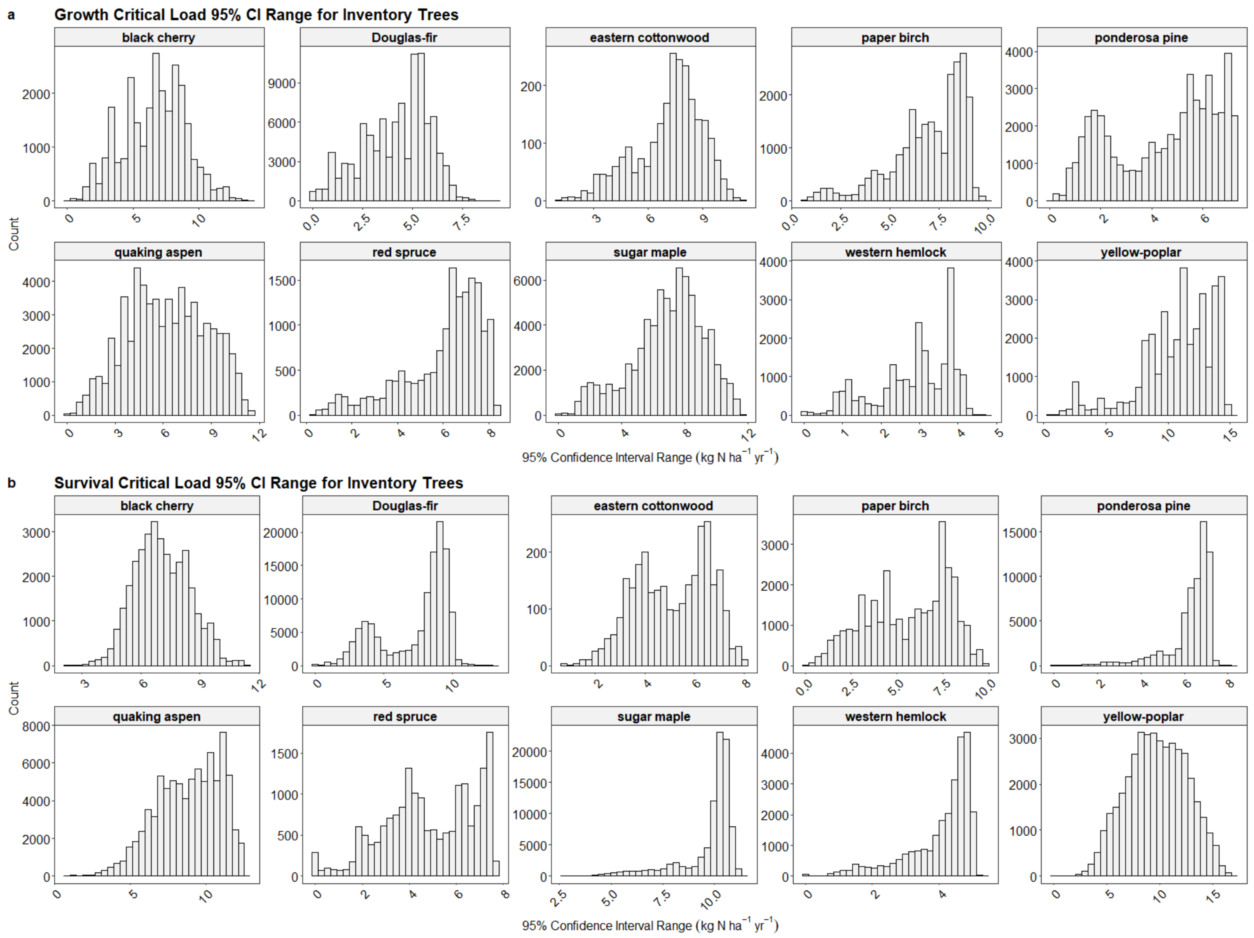


**Figure S31.** N critical load 95% CI range for inventory trees for (a) growth and (b) survival. Each individual tree species is shown. Bins represent the 95% CI range for individual inventory trees from the bootstrapping ensemble methodology.

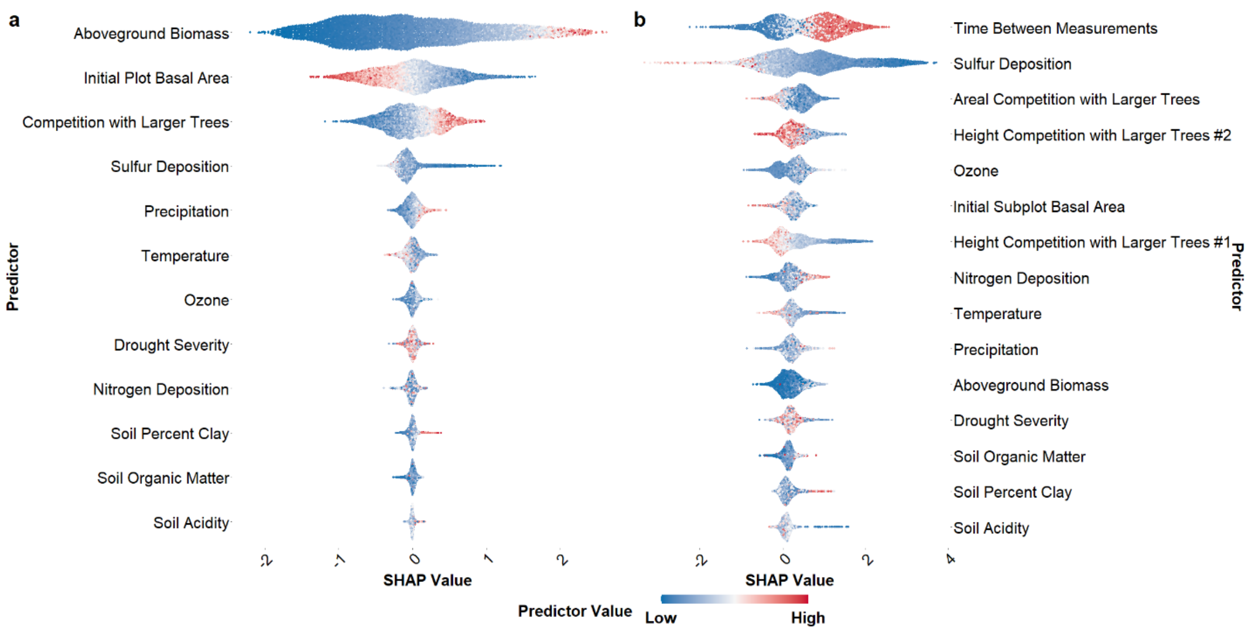


**Figure S32.** SHAP value results for red spruce for both **(a)** growth and **(b)** survival probability XGBoost model predictions. Each predictor used in the modeling is shown as a separate y-axis bin. Positive SHAP values mean the predictor resulted in an increased growth rate or survival probability while negative SHAP values mean a decreased value was predicted. The color gradient illustrates whether the predictor value was relatively low (blue) or high (red) against all values of the respective predictor. Each point represents a single inventory tree. Predictors are ordered by their absolute median SHAP value.

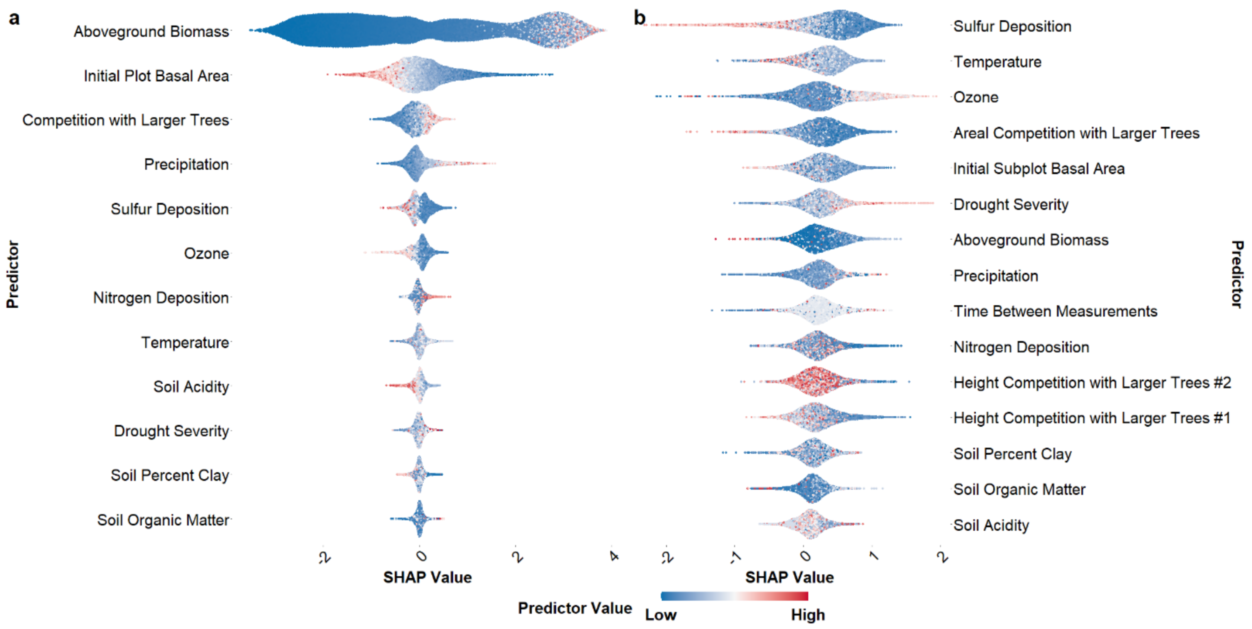


**Figure S33.** SHAP value results for ponderosa pine for both **(a)** growth and **(b)** survival probability XGBoost model predictions. Each predictor used in the modeling is shown as a separate y-axis bin. Positive SHAP values mean the predictor resulted in an increased growth rate or survival probability while negative SHAP values mean a decreased value was predicted. The color gradient illustrates whether the predictor value was relatively low (blue) or high (red) against all values of the respective predictor. Each point represents a single inventory tree. Predictors are ordered by their absolute median SHAP value.

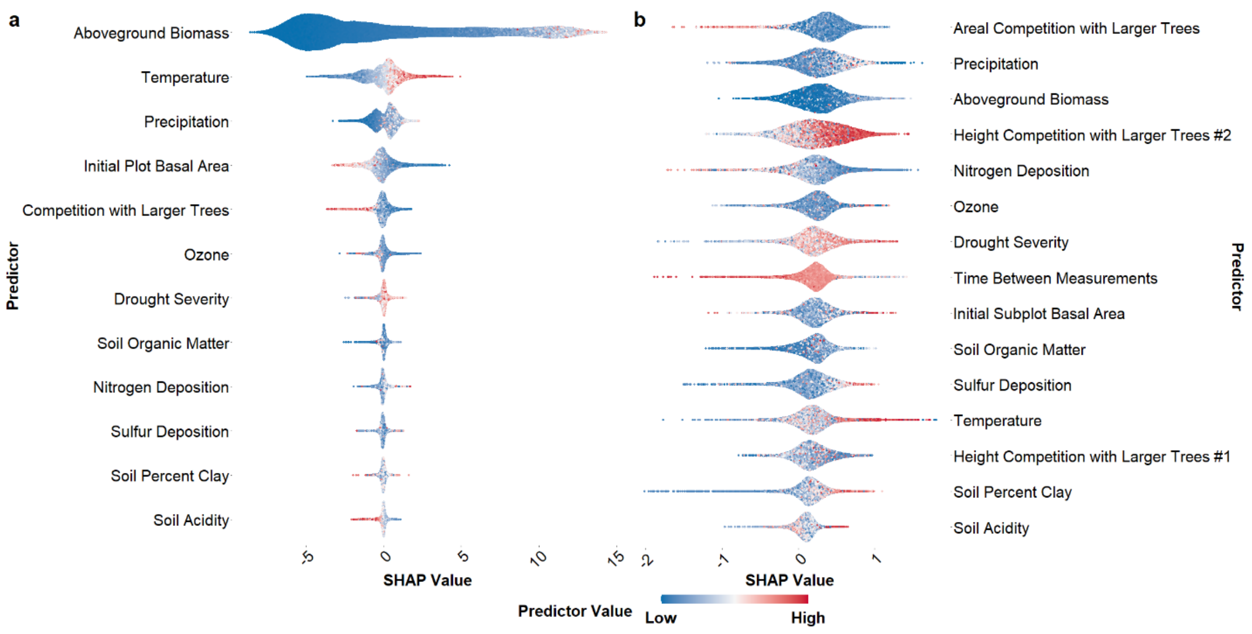


**Figure S34.** SHAP value results for Douglas-fir for both **(a)** growth and **(b)** survival probability XGBoost model predictions. Each predictor used in the modeling is shown as a separate y-axis bin. Positive SHAP values mean the predictor resulted in an increased growth rate or survival probability while negative SHAP values mean a decreased value was predicted. The color gradient illustrates whether the predictor value was relatively low (blue) or high (red) against all values of the respective predictor. Each point represents a single inventory tree. Predictors are ordered by their absolute median SHAP value.

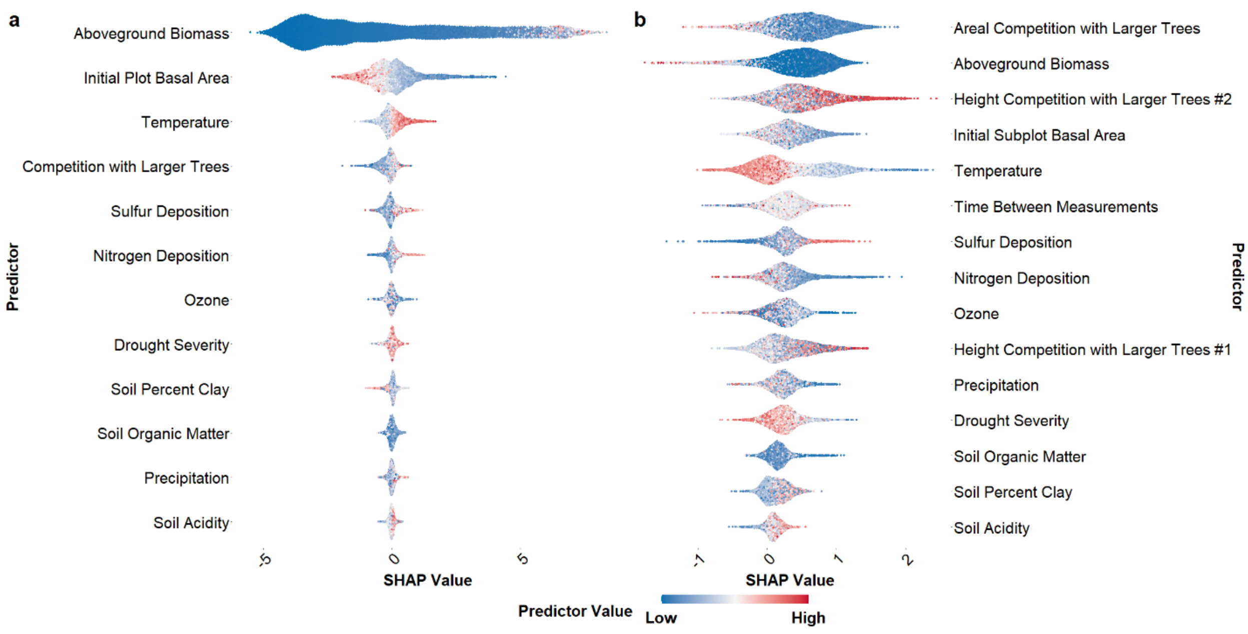


**Figure S35.** SHAP value results for western hemlock for both **(a)** growth and **(b)** survival probability XGBoost model predictions. Each predictor used in the modeling is shown as a separate y-axis bin. Positive SHAP values mean the predictor resulted in an increased growth rate or survival probability while negative SHAP values mean a decreased value was predicted. The color gradient illustrates whether the predictor value was relatively low (blue) or high (red) against all values of the respective predictor. Each point represents a single inventory tree. Predictors are ordered by their absolute median SHAP value.

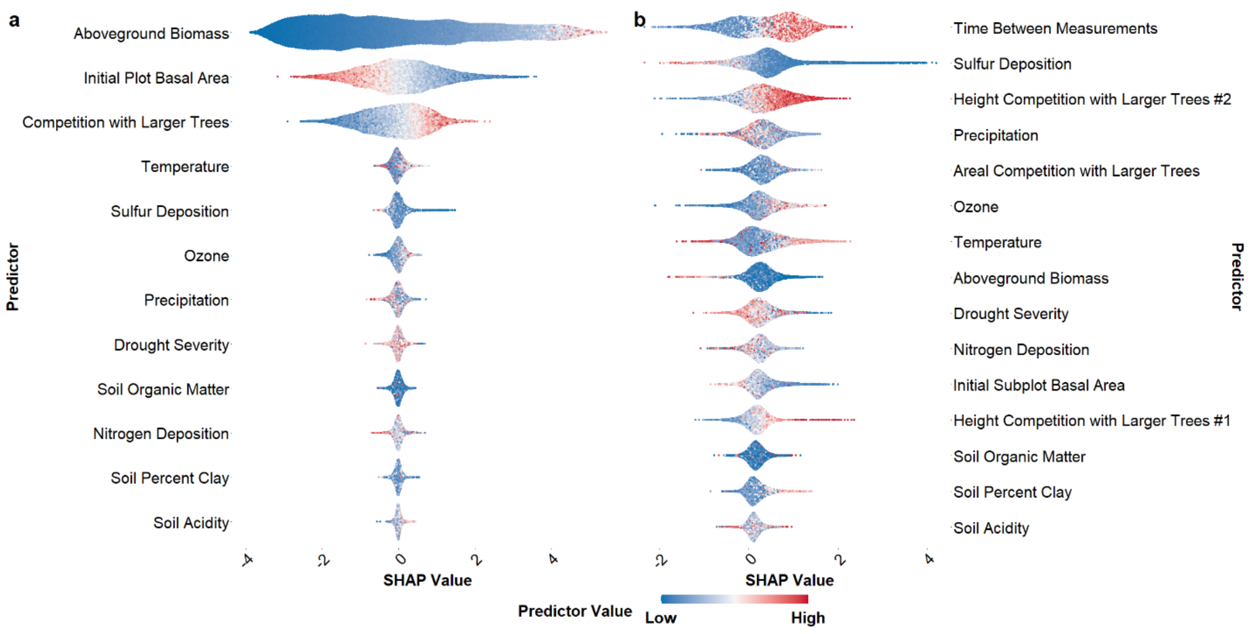


**Figure S36.** SHAP value results for sugar maple for both **(a)** growth and **(b)** survival probability XGBoost model predictions. Each predictor used in the modeling is shown as a separate y-axis bin. Positive SHAP values mean the predictor resulted in an increased growth rate or survival probability while negative SHAP values mean a decreased value was predicted. The color gradient illustrates whether the predictor value was relatively low (blue) or high (red) against all values of the respective predictor. Each point represents a single inventory tree. Predictors are ordered by their absolute median SHAP value.

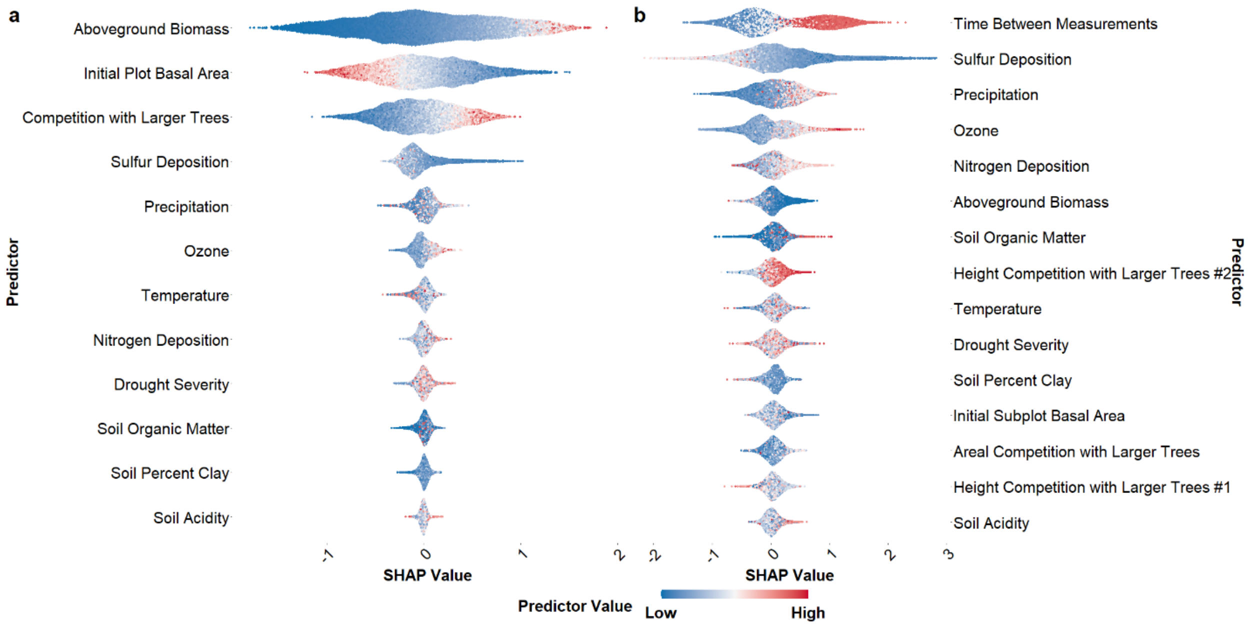


**Figure S37.** SHAP value results for paper birch for both **(a)** growth and **(b)** survival probability XGBoost model predictions. Each predictor used in the modeling is shown as a separate y-axis bin. Positive SHAP values mean the predictor resulted in an increased growth rate or survival probability while negative SHAP values mean a decreased value was predicted. The color gradient illustrates whether the predictor value was relatively low (blue) or high (red) against all values of the respective predictor. Each point represents a single inventory tree. Predictors are ordered by their absolute median SHAP value.

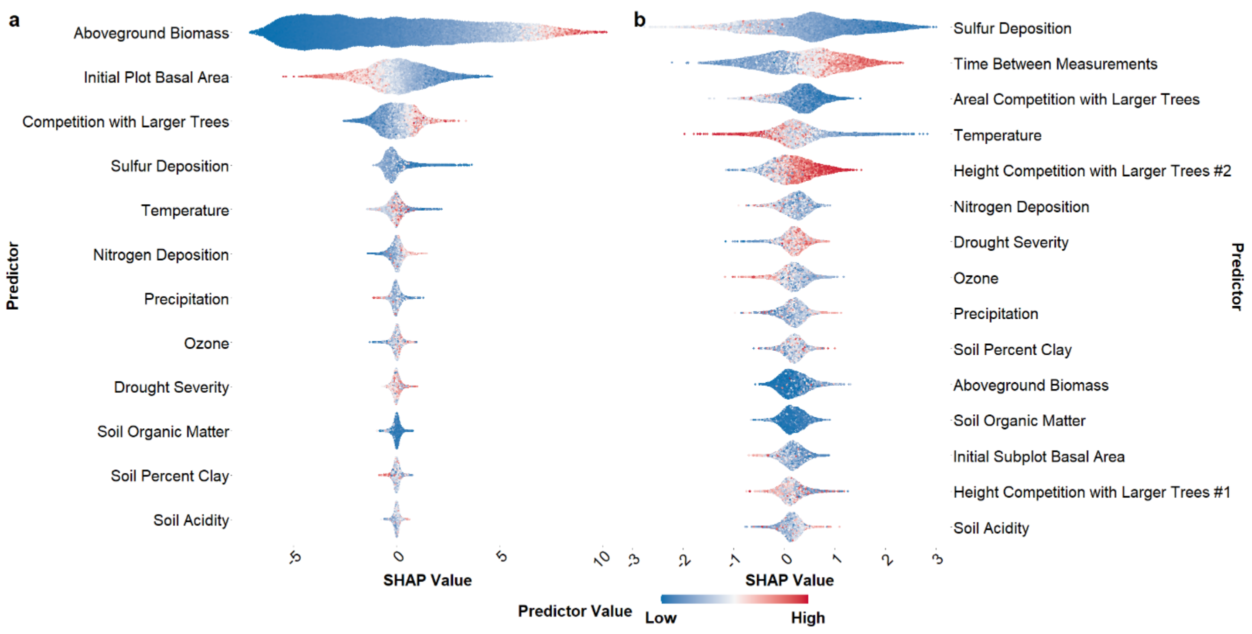


**Figure S38.** SHAP value results for yellow-poplar both **(a)** growth and **(b)** survival probability XGBoost model predictions. Each predictor used in the modeling is shown as a separate y-axis bin. Positive SHAP values mean the predictor resulted in an increased growth rate or survival probability while negative SHAP values mean a decreased value was predicted. The color gradient illustrates whether the predictor value was relatively low (blue) or high (red) against all values of the respective predictor. Each point represents a single inventory tree. Predictors are ordered by their absolute median SHAP value.

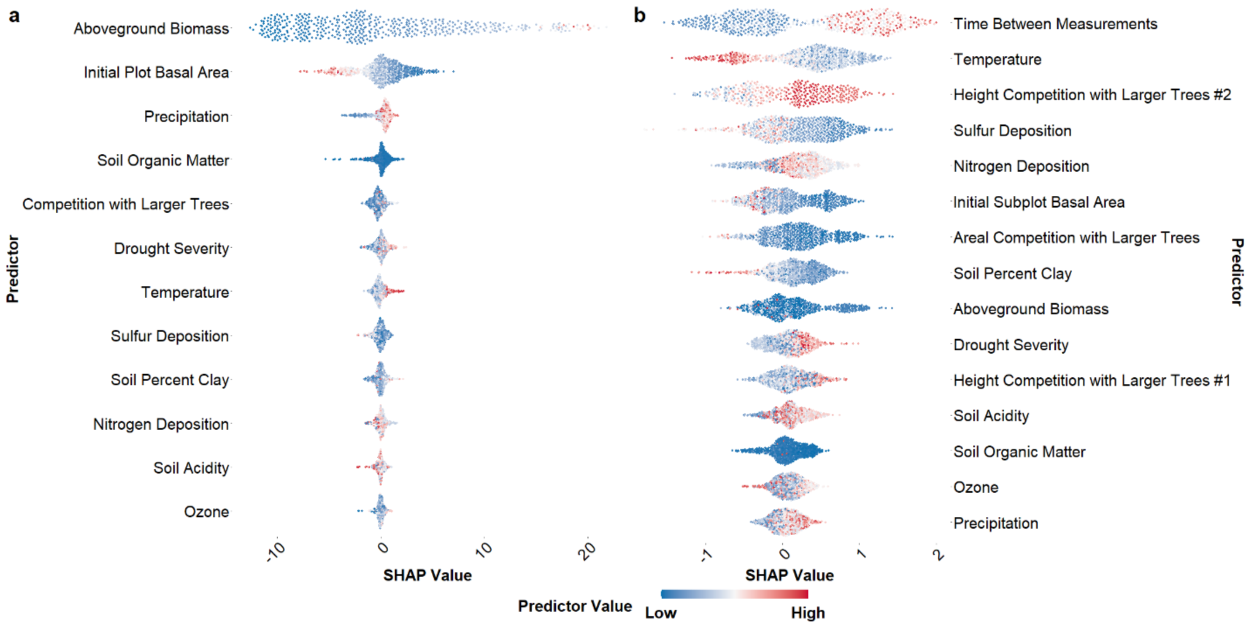


**Figure S39.** SHAP value results for eastern cottonwood for both **(a)** growth and **(b)** survival probability XGBoost model predictions. Each predictor used in the modeling is shown as a separate y-axis bin. Positive SHAP values mean the predictor resulted in an increased growth rate or survival probability while negative SHAP values mean a decreased value was predicted. The color gradient illustrates whether the predictor value was relatively low (blue) or high (red) against all values of the respective predictor. Each point represents a single inventory tree. Predictors are ordered by their absolute median SHAP value.

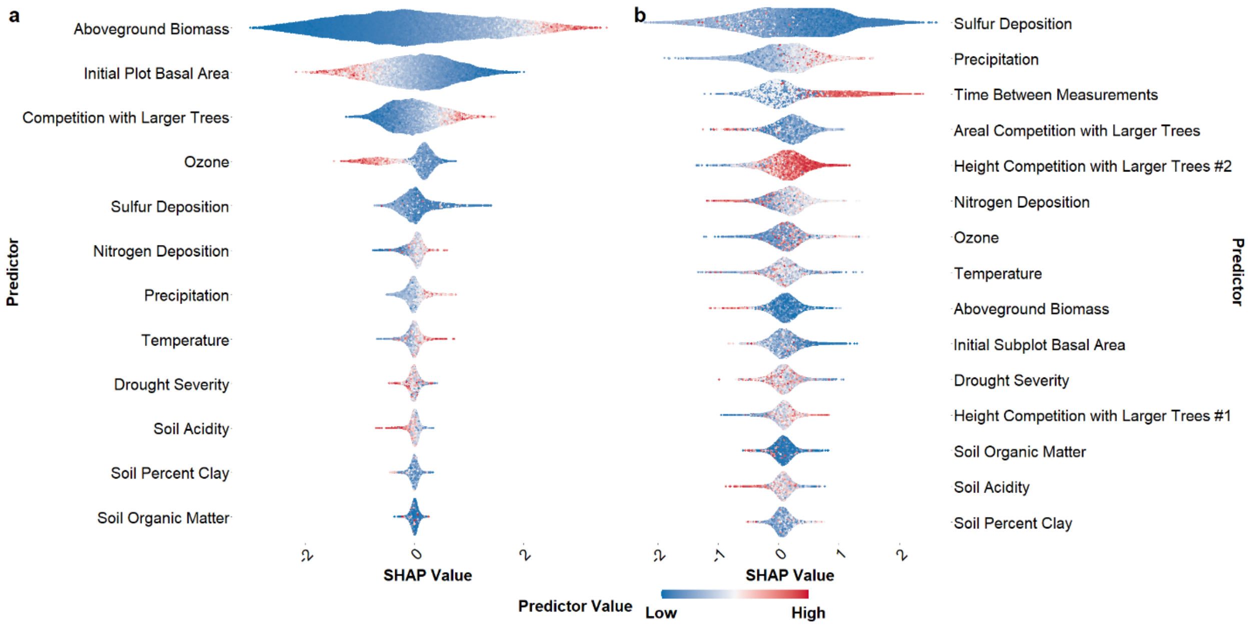


**Figure S40.** SHAP value results for quaking aspen for both **(a)** growth and **(b)** survival probability XGBoost model predictions. Each predictor used in the modeling is shown as a separate y-axis bin. Positive SHAP values mean the predictor resulted in an increased growth rate or survival probability while negative SHAP values mean a decreased value was predicted. The color gradient illustrates whether the predictor value was relatively low (blue) or high (red) against all values of the respective predictor. Each point represents a single inventory tree. Predictors are ordered by their absolute median SHAP value.

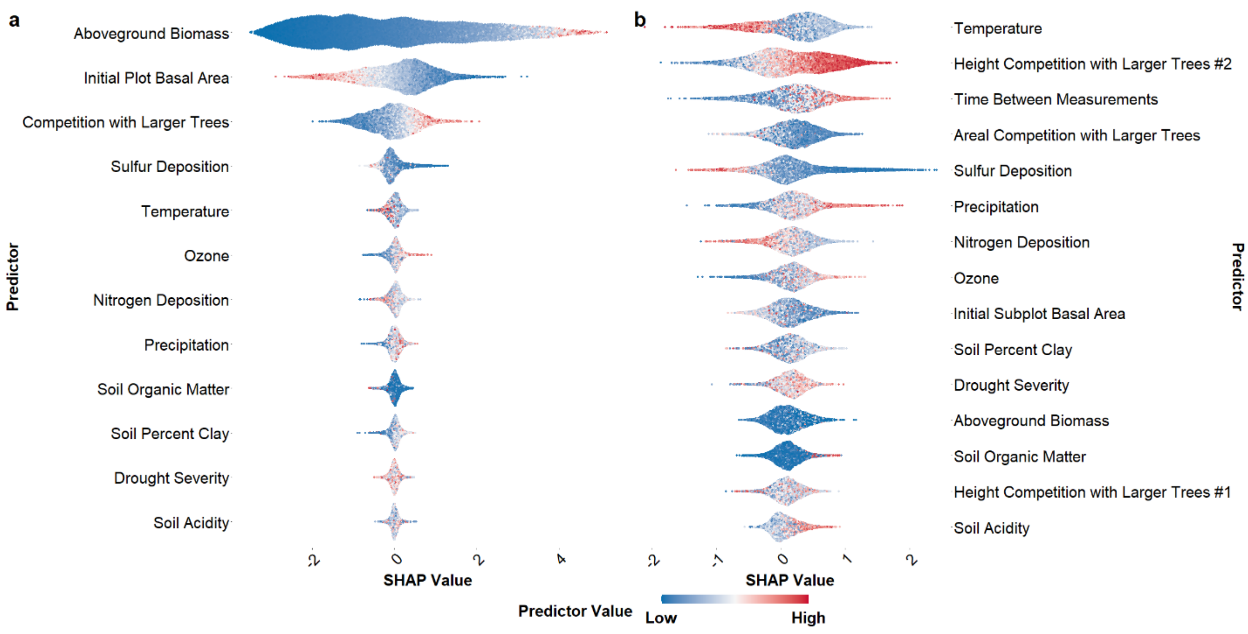


**Figure S41.** SHAP value results for black cherry for both **(a)** growth and **(b)** survival probability XGBoost model predictions. Each predictor used in the modeling is shown as a separate y-axis bin. Positive SHAP values mean the predictor resulted in an increased growth rate or survival probability while negative SHAP values mean a decreased value was predicted. The color gradient illustrates whether the predictor value was relatively low (blue) or high (red) against all values of the respective predictor. Each point represents a single inventory tree. Predictors are ordered by their absolute median SHAP value.

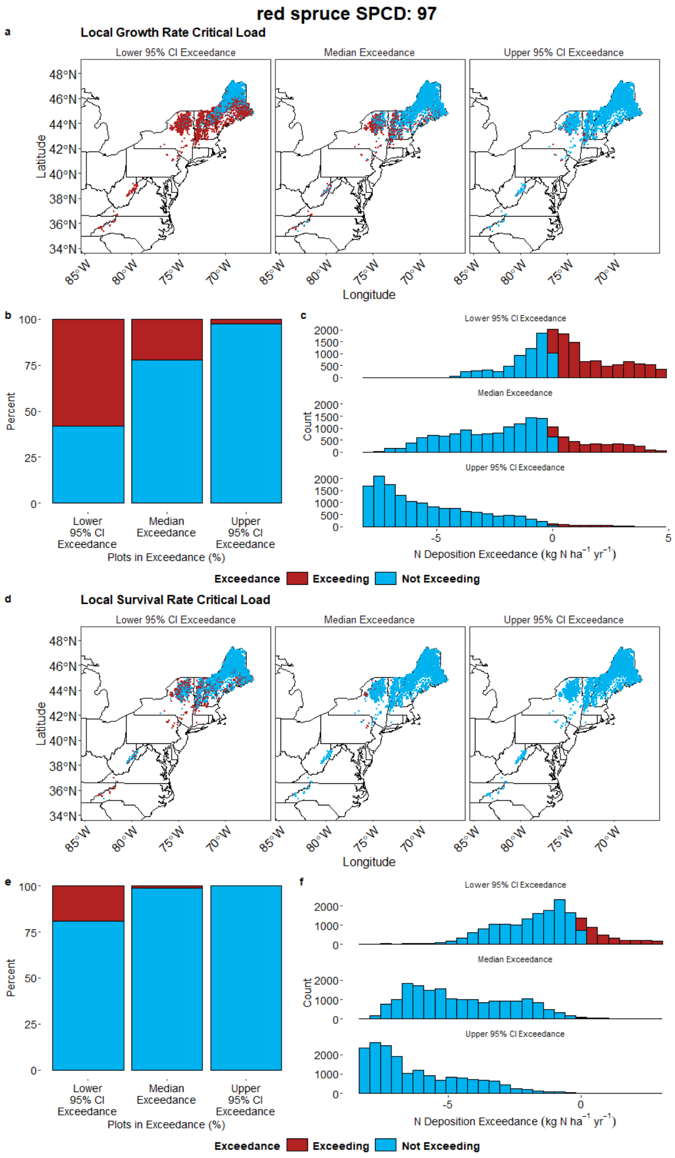


**Figure S42.** (a) Maps of 2019-2021 N CL exceedances for growth based on the median and lower and upper 95% CI for red spruce across its range, (b) the percent of red spruce FIA plots in exceedance in 2019-2021 on the three aggregations for growth, and (c) the magnitude of N deposition exceedance in relation to the N CL based on the three aggregations for growth. (d) Maps of 2019-2021 N CL exceedances for survival based on the median and lower and upper 95% CI for red spruce across its range, (e) the percent of red spruce FIA plots in exceedance in 2019-2021 on the three aggregations for survival, and (f) the magnitude of N deposition exceedance in relation to the N CL based on the three aggregations for survival. Percentages in red are plots that are in exceedance while percentages in blue are not exceeding. Percentages or plots in red are in exceedance while percentages or plots in blue are not exceeding.

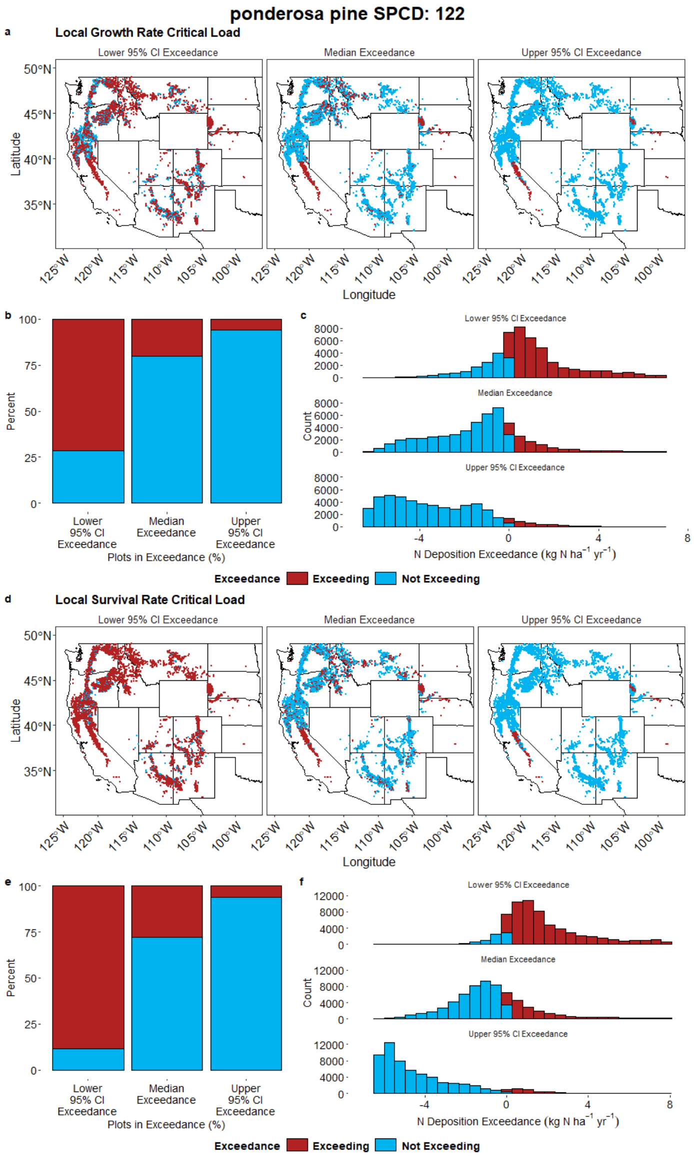


**Figure S43.** (a) Maps of 2019-2021 N CL exceedances for growth based on the median and lower and upper 95% CI for ponderosa pine across its range, (b) the percent of ponderosa pine FIA plots in exceedance in 2019-2021 on the three aggregations for growth, and (c) the magnitude of N deposition exceedance in relation to the N CL based on the three aggregations for growth. (d) Maps of 2019-2021 N CL exceedances for survival based on the median and lower and upper 95% CI for ponderosa pine across its range, (e) the percent of ponderosa pine FIA plots in exceedance in 2019-2021 on the three aggregations for survival, and (f) the magnitude of N deposition exceedance in relation to the N CL based on the three aggregations for survival. Percentages in red are plots that are in exceedance while percentages in blue are not exceeding. Percentages or plots in red are in exceedance while percentages or plots in blue are not exceeding.

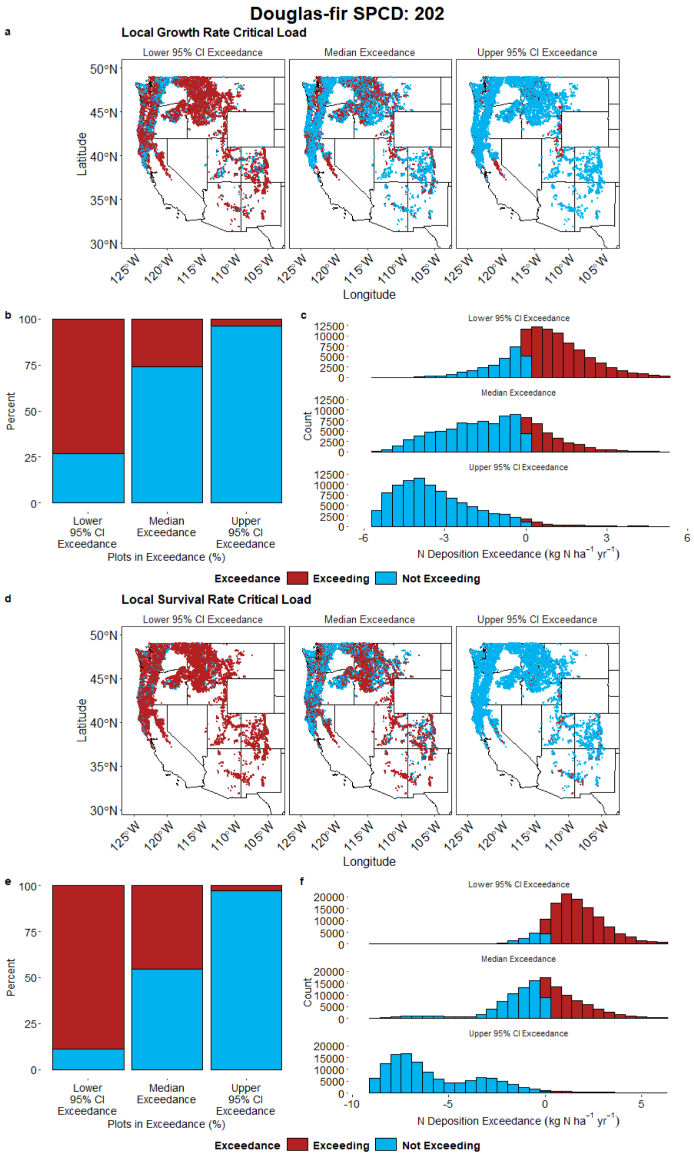


**Figure S44.** (a) Maps of 2019-2021 N CL exceedances for growth based on the median and lower and upper 95% CI for Douglas-fir across its range, (b) the percent of Douglas-fir FIA plots in exceedance in 2019-2021 on the three aggregations for growth, and (c) the magnitude of N deposition exceedance in relation to the N CL based on the three aggregations for growth. (d) Maps of 2019-2021 N CL exceedances for survival based on the median and lower and upper 95% CI for Douglas-fir across its range, (e) the percent of Douglas-fir FIA plots in exceedance in 2019-2021 on the three aggregations for survival, and (f) the magnitude of N deposition exceedance in relation to the N CL based on the three aggregations for survival. Percentages in red are plots that are in exceedance while percentages in blue are not exceeding. Percentages or plots in red are in exceedance while percentages or plots in blue are not exceeding.

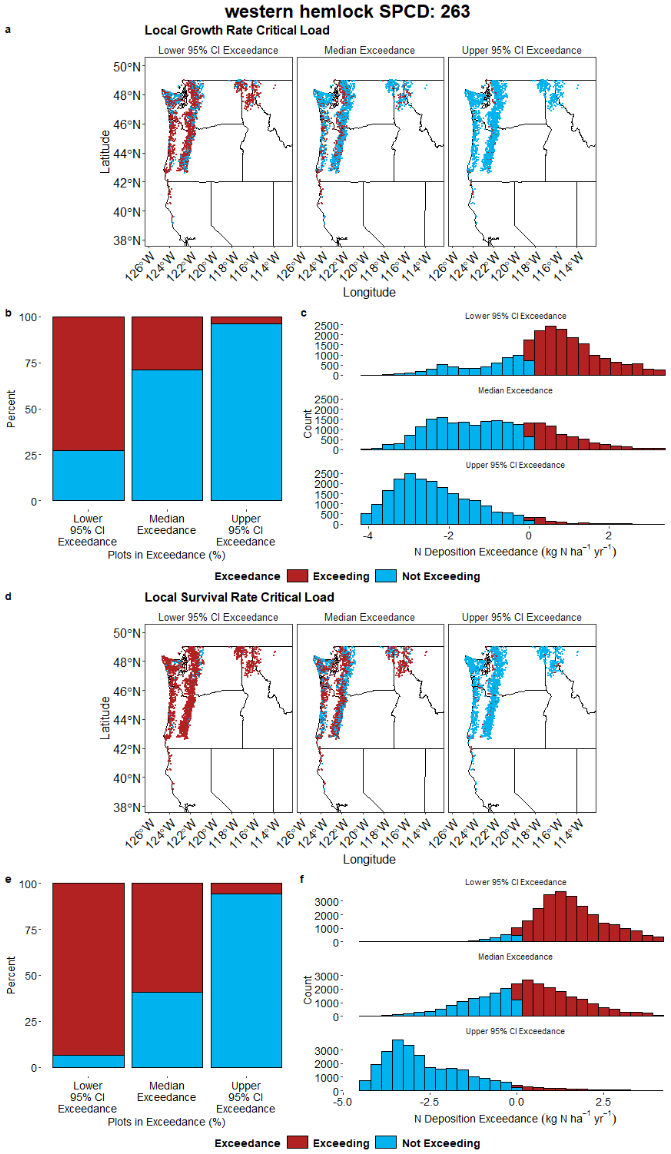


**Figure S45.** (a) Maps of 2019-2021 N CL exceedances for growth based on the median and lower and upper 95% CI for western hemlock across its range, (b) the percent of western hemlock FIA plots in exceedance in 2019-2021 on the three aggregations for growth, and (c) the magnitude of N deposition exceedance in relation to the N CL based on the three aggregations for growth. (d) Maps of 2019-2021 N CL exceedances for survival based on the median and lower and upper 95% CI for western hemlock across its range, (e) the percent of western hemlock FIA plots in exceedance in 2019-2021 on the three aggregations for survival, and (f) the magnitude of N deposition exceedance in relation to the N CL based on the three aggregations for survival. Percentages in red are plots that are in exceedance while percentages in blue are not exceeding. Percentages or plots in red are in exceedance while percentages or plots in blue are not exceeding.

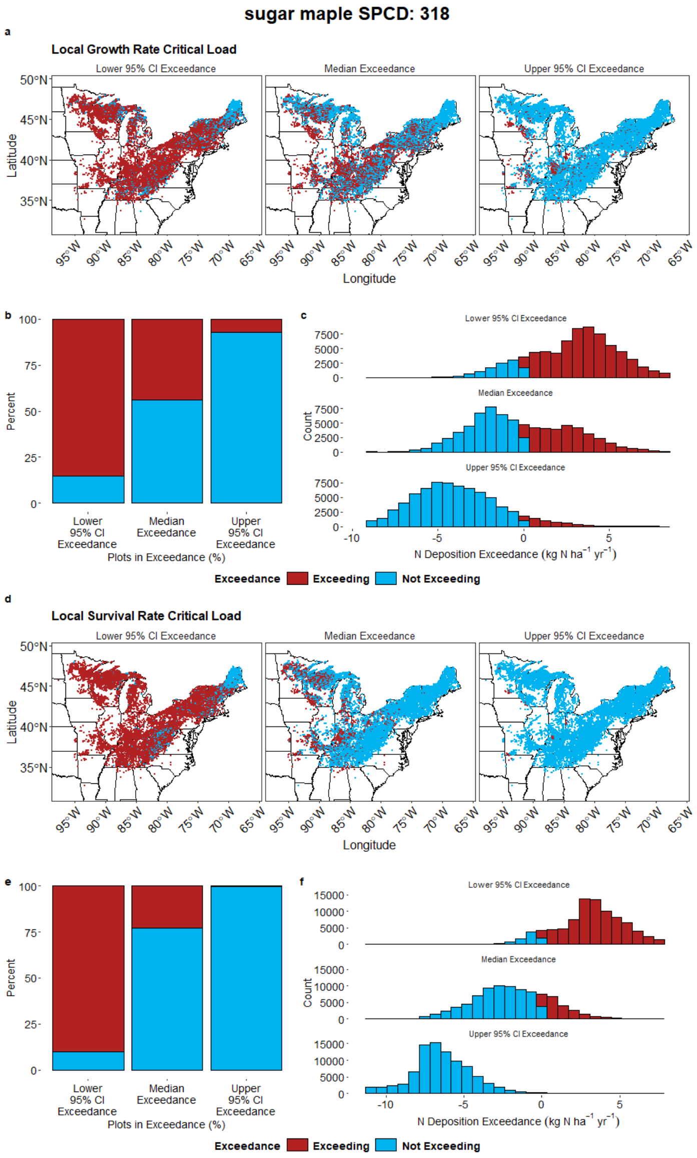


**Figure S46.** (a) Maps of 2019-2021 N CL exceedances for growth based on the median and lower and upper 95% CI for sugar maple across its range, (b) the percent of sugar maple FIA plots in exceedance in 2019-2021 on the three aggregations for growth, and (c) the magnitude of N deposition exceedance in relation to the N CL based on the three aggregations for growth. (d) Maps of 2019-2021 N CL exceedances for survival based on the median and lower and upper 95% CI for sugar maple across its range, (e) the percent of sugar maple FIA plots in exceedance in 2019-2021 on the three aggregations for survival, and (f) the magnitude of N deposition exceedance in relation to the N CL based on the three aggregations for survival. Percentages in red are plots that are in exceedance while percentages in blue are not exceeding. Percentages or plots in red are in exceedance while percentages or plots in blue are not exceeding.

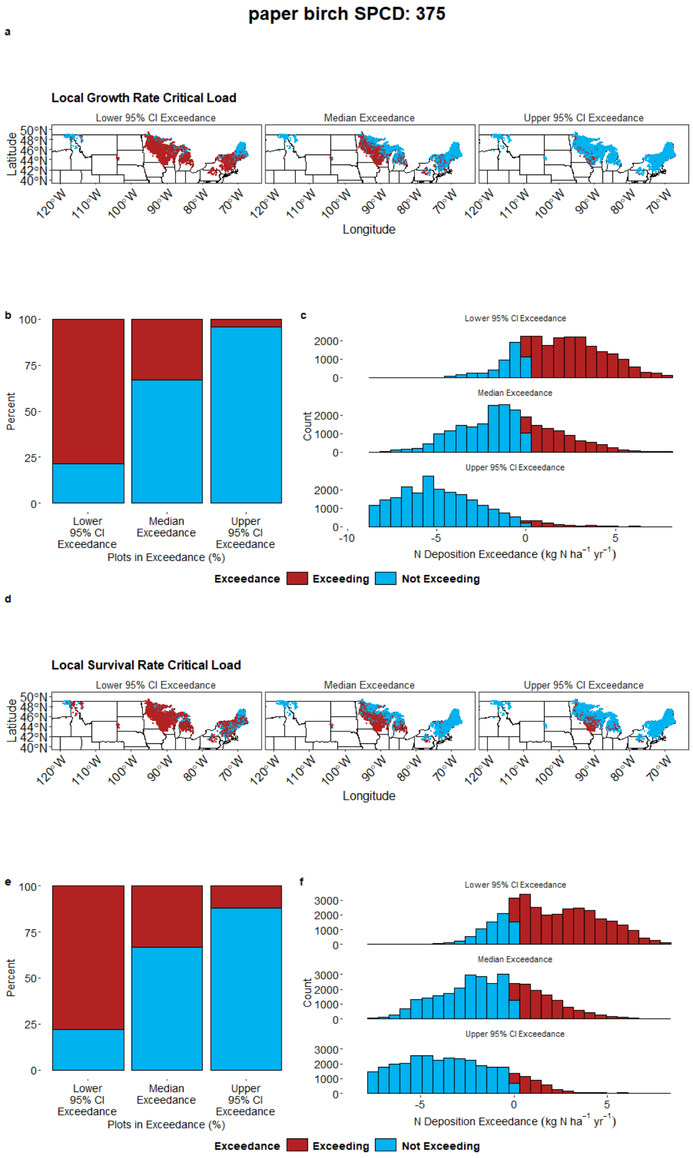


**Figure S47.** (a) Maps of 2019-2021 N CL exceedances for growth based on the median and lower and upper 95% CI for paper birch across its range, (b) the percent of paper birch FIA plots in exceedance in 2019-2021 on the three aggregations for growth, and (c) the magnitude of N deposition exceedance in relation to the N CL based on the three aggregations for growth. (d) Maps of 2019-2021 N CL exceedances for survival based on the median and lower and upper 95% CI for paper birch across its range, (e) the percent of paper birch FIA plots in exceedance in 2019-2021 on the three aggregations for survival, and (f) the magnitude of N deposition exceedance in relation to the N CL based on the three aggregations for survival. Percentages in red are plots that are in exceedance while percentages in blue are not exceeding. Percentages or plots in red are in exceedance while percentages or plots in blue are not exceeding.

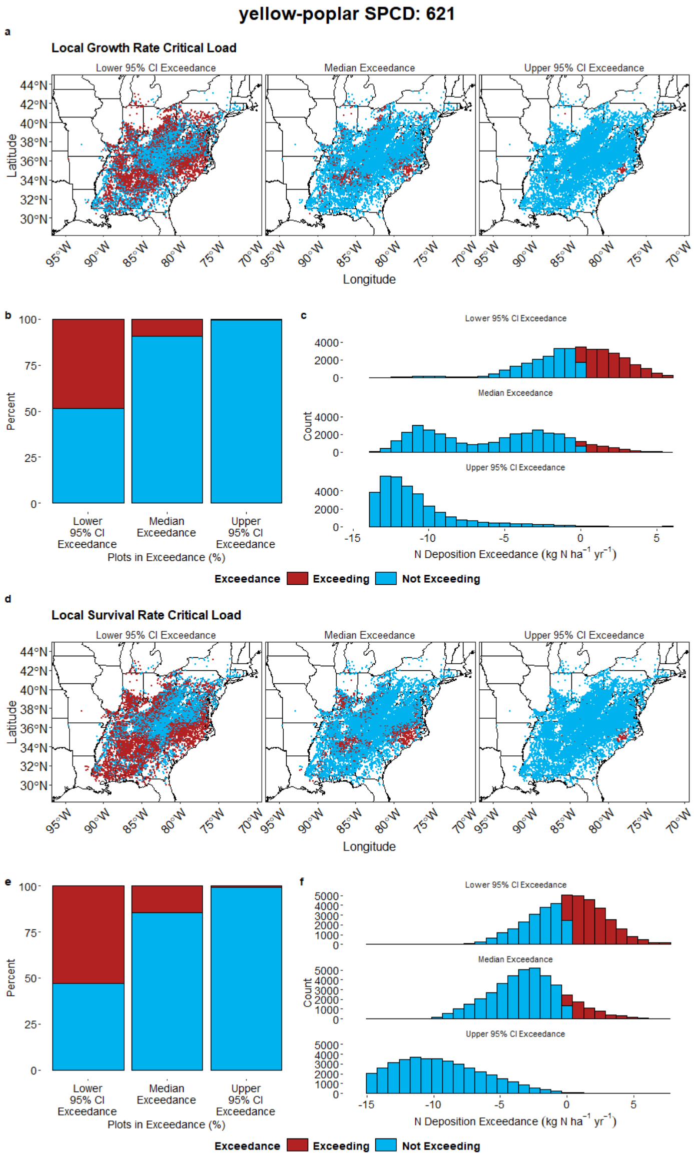


**Figure S48.** (a) Maps of 2019-2021 N CL exceedances for growth based on the median and lower and upper 95% CI for yellow-poplar across its range, (b) the percent of yellow-poplar FIA plots in exceedance in 2019-2021 on the three aggregations for growth, and (c) the magnitude of N deposition exceedance in relation to the N CL based on the three aggregations for growth. (d) Maps of 2019-2021 N CL exceedances for survival based on the median and lower and upper 95% CI for yellow-poplar across its range, (e) the percent of yellow-poplar FIA plots in exceedance in 2019-2021 on the three aggregations for survival, and (f) the magnitude of N deposition exceedance in relation to the N CL based on the three aggregations for survival. Percentages in red are plots that are in exceedance while percentages in blue are not exceeding. Percentages or plots in red are in exceedance while percentages or plots in blue are not exceeding.

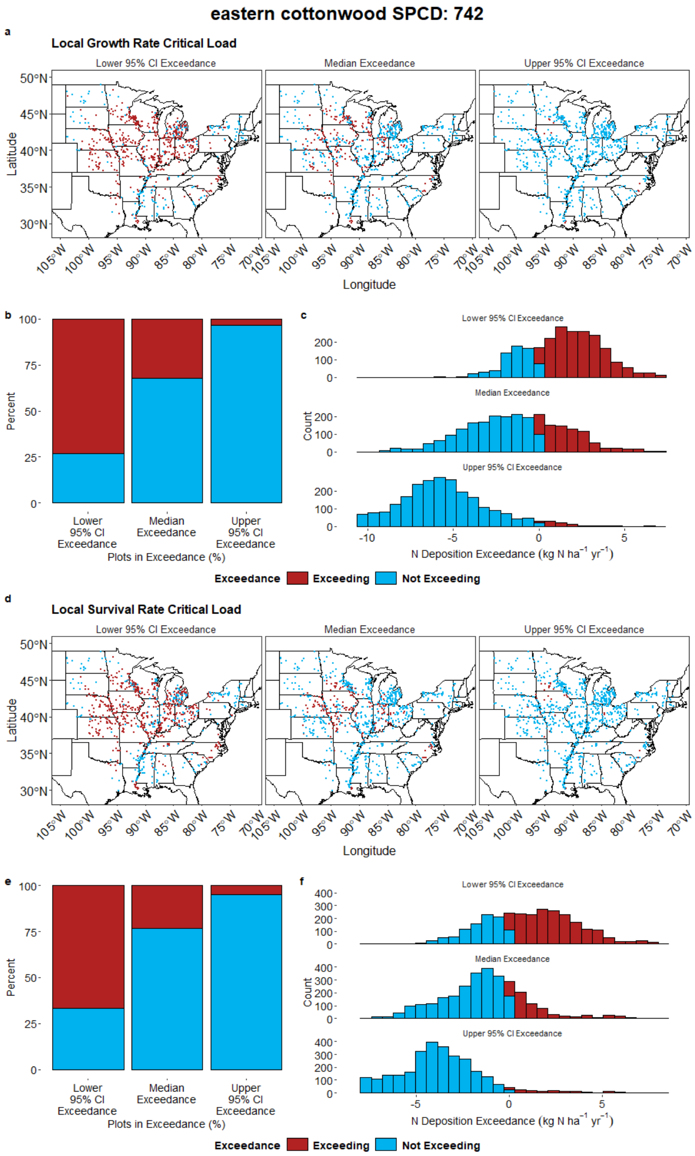


**Figure S49.** (a) Maps of 2019-2021 N CL exceedances for growth based on the median and lower and upper 95% CI for eastern cottonwood across its range, (b) the percent of eastern cottonwood FIA plots in exceedance in 2019-2021 on the three aggregations for growth, and (c) the magnitude of N deposition exceedance in relation to the N CL based on the three aggregations for growth. (d) Maps of 2019-2021 N CL exceedances for survival based on the median and lower and upper 95% CI for eastern cottonwood across its range, (e) the percent of eastern cottonwood FIA plots in exceedance in 2019-2021 on the three aggregations for survival, and (f) the magnitude of N deposition exceedance in relation to the N CL based on the three aggregations for survival. Percentages in red are plots that are in exceedance while percentages in blue are not exceeding. Percentages or plots in red are in exceedance while percentages or plots in blue are not exceeding.

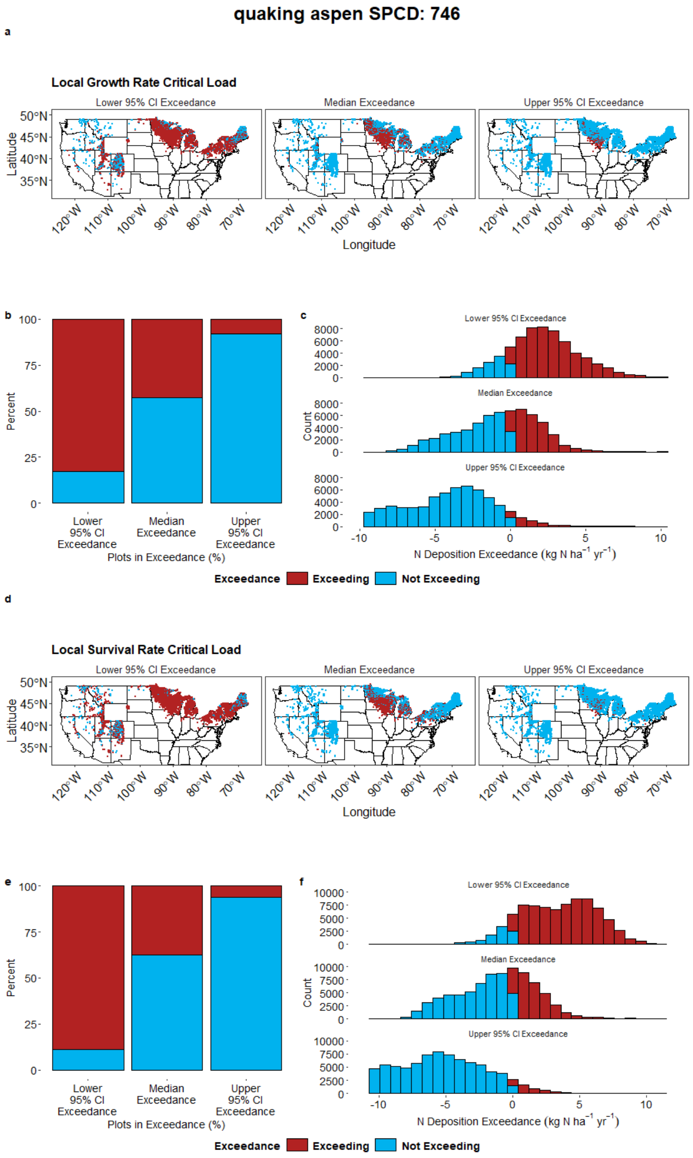


**Figure S50.** (a) Maps of 2019-2021 N CL exceedances for growth based on the median and lower and upper 95% CI for quaking aspen across its range, (b) the percent of quaking aspen FIA plots in exceedance in 2019-2021 on the three aggregations for growth, and (c) the magnitude of N deposition exceedance in relation to the N CL based on the three aggregations for growth. (d) Maps of 2019-2021 N CL exceedances for survival based on the median and lower and upper 95% CI for quaking aspen across its range, (e) the percent of quaking aspen FIA plots in exceedance in 2019-2021 on the three aggregations for survival, and (f) the magnitude of N deposition exceedance in relation to the N CL based on the three aggregations for survival. Percentages in red are plots that are in exceedance while percentages in blue are not exceeding. Percentages or plots in red are in exceedance while percentages or plots in blue are not exceeding.

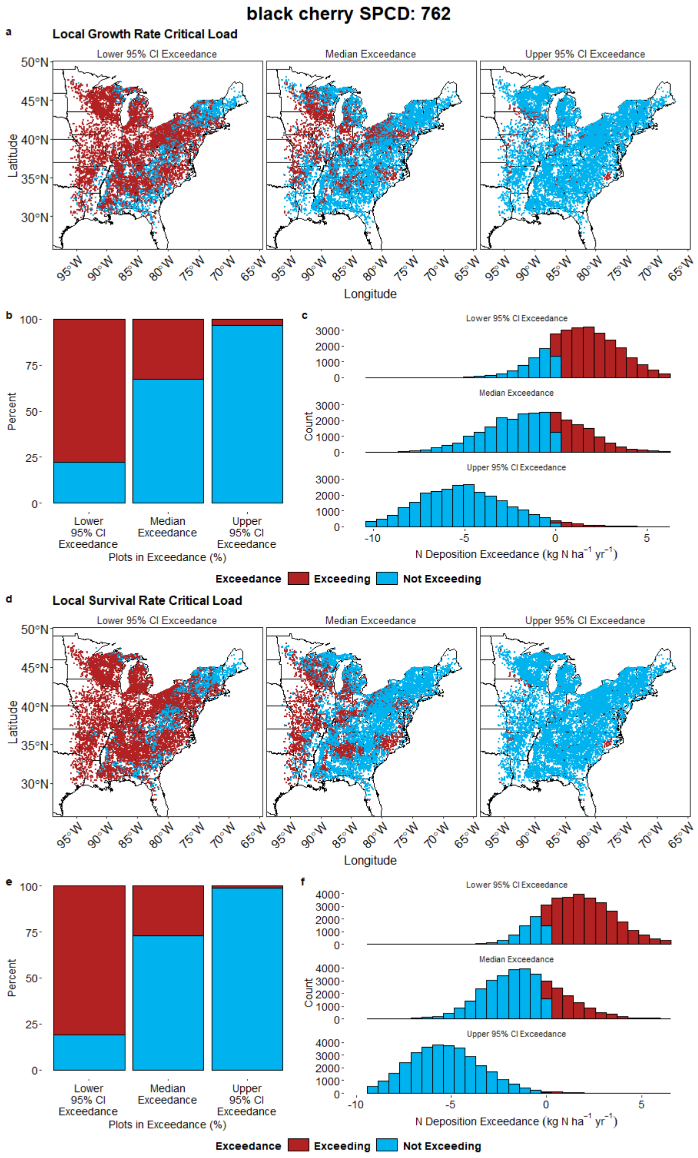


**Figure S51.** (a) Maps of 2019-2021 N CL exceedances for growth based on the median and lower and upper 95% CI for black cherry across its range, (b) the percent of black cherry FIA plots in exceedance in 2019-2021 on the three aggregations for growth, and (c) the magnitude of N deposition exceedance in relation to the N CL based on the three aggregations for growth. (d) Maps of 2019-2021 N CL exceedances for survival based on the median and lower and upper 95% CI for black cherry across its range, (e) the percent of black cherry FIA plots in exceedance in 2019-2021 on the three aggregations for survival, and (f) the magnitude of N deposition exceedance in relation to the N CL based on the three aggregations for survival. Percentages in red are plots that are in exceedance while percentages in blue are not exceeding. Percentages or plots in red are in exceedance while percentages or plots in blue are not exceeding.

## References

Horn, K. J., R. Quinn Thomas, C. M. Clark, L. H. Pardo, M. E. Fenn, G. B. Lawrence, S. S. Perakis, E. A. H. Smithwick, D. Baldwin, S. Braun, A. Nordin, C. H. Perry, J. N. Phelan, P. G. Schaberg, S. B. S. Clair, R. Warby, and S. Watmough. 2018. Growth and survival relationships of 71 tree species with nitrogen and sulfur deposition across the conterminous U.S. PLoS ONE 13:e0205296.

Pavlovic, N. R., S. Y. Chang, J. Huang, K. Craig, C. Clark, K. Horn, and C. T. Driscoll. 2023. Empirical nitrogen and sulfur critical loads of U.S. tree species and their uncertainties with machine learning. Science of the Total Environment 857:159252.
